# Supplementary material for: Global genetic diversity, introgression, and evolutionary adaptation of indicine cattle revealed by whole genome sequencing
Source: Nat Commun. 2023 Nov 28;14:7803. doi: 10.1038/s41467-023-43626-z (PMC10684552; doi:10.1038/s41467-023-43626-z)
Supplement: Supplementary file 1 — Supplementary Information [file 41467_2023_43626_MOESM1_ESM.pdf]

Global genetic diversity, introgression and evolutionary adaptation of indicine cattle revealed by whole genome sequencing

Ningbo Chen[1,#], Xiaoting Xia[1,#], Quratulain Hanif[2,3,#], Fengwei Zhang[1,#], Ruihua Dang[1,#], Bizhi Huang[4,#], Yang Lyu[1], Xiaoyu Luo[1], Hucai Zhang[5], Huixuan Yan[1], Shikang Wang[1], Fuwen Wang[1], Jialei Chen[1], Xiwen Guan[1], Yangkai Liu[1], Shuang Li[1], Liangliang Jin[1], Pengfei Wang[1], Luyang Sun[1], Jicai Zhang[4], Jianyong Liu[4], Kaixing Qu[6], Yanhong Cao[7], Junli Sun[7], Yuying Liao[8], Zhengzhong Xiao[7], Ming Cai[4], Lan Mu[9], AMAM Zonaed Siddiki[10], Muhammad Asif[2], Shahid Mansoor[2], Masroor Ellahi Babar[11], Tanveer Hussain[12], Gamamada Liyanage Lalanie Pradeepa Silva[13], Neena Amatya Gorkhali[14], Endashaw Terefe[15,16], Gurja Belay[17], Abdulfatai Tijjani[16,18], Tsadkan Zegeye[19], Mebrate Genet Gebre[20], Yun Ma[21], Yu Wang[1], Yongzhen Huang[1], Xianyong Lan[1], Hong Chen[1], Nicola Rambaldi Migliore[22], Giulia Colombo[22], Ornella Semino[22], Alessandro Achilli[22], Mikkel-Holger S. Sinding[23], Johannes A Lenstra[24], Haijian Cheng[1,25], Wenfa Lu [26], Olivier Hanotte[16,27*], Jianlin Han[3,28,29*], Yu Jiang[1,30*], Chuzhao Lei[1*]

1 Key Laboratory of Animal Genetics, Breeding and Reproduction of Shaanxi Province, College of Animal Science and Technology, Northwest A&F University, Yangling 712100, China.
2 National Institute for Biotechnology and Genetic Engineering, Faisalabad 38000, Pakistan.
3 CAAS-ILRI Joint Laboratory on Livestock and Forage Genetic Resources, Institute of Animal Science, Chinese Academy of Agricultural Sciences (CAAS), Beijing 100193, China.
4 Yunnan Academy of Grassland and Animal Science, Kunming 650212, China.
5 Institute for Ecological Research and Pollution Control of Plateau Lakes, School of Ecology and Environment Science, Yunnan University, Kunming 650500, China.
6 Academy of Science and Technology, Chuxiong Normal University, Chuxiong 675000, China.
7 Guangxi Vocational University of Agriculture, Nanning 530007, China.
8 Guangxi Veterinary Research Institute, Guangxi Key Laboratory of Veterinary Biotechnology, Nanning 530001, China.
9 College of Landscape and Horticulture, Southwest Forestry University, Kunming 650224, China.
10 Genomics Research Group, Department of Pathology and Parasitology, Faculty of Veterinary Medicine, Chattogram Veterinary and Animal Sciences University (CVASU), Chattogram-4225, Bangladesh.
11 The University of Agriculture, Dera Ismail Khan, Khyber Pakhtunkhwa 29050, Pakistan.
12 Department of Molecular Biology, Virtual University of Pakistan, Islamabad 44100, Pakistan.
13 Department of Animal Science, University of Peradeniya, Peradeniya 20400, Sri Lanka.
14 National Animal Breeding and Genetics Centre, National Animal Science Research Institute, Nepal Agriculture Research Council, Khumaltar, Lalitpur 45200, Nepal.
15 College of Agriculture and Environmental Science, Department of Animal Science, Arsi University, Asella, Ethiopia.
16 International Livestock Research Institute (ILRI), P.O. Box 5689, Addis Ababa 1000, Ethiopia.
17 College of Natural and Computational Sciences, the School of Graduate Studies, Addis Ababa University, Addis Ababa 1000, Ethiopia.
18 The Jackson Laboratory, Bar Harbor ME. 04609, USA.
19 Mekelle Agricultural Research Center, P.O. Box 258 Mekelle, Tigray 7000, Ethiopia.
20 School of Animal and Rangeland Science, College of Agriculture, Haramaya University, Haramaya, Oromia 2040, Ethiopia.
21 Key Laboratory of Ruminant Molecular and Cellular Breeding of Ningxia Hui Autonomous Region, School of Agriculture, Ningxia University, Yinchuan 750000, China.
22 Department of Biology and Biotechnology "Lazzaro Spallanzani", University of Pavia, Pavia 27100, Italy.
23 Section for Computational and RNA Biology, Department of Biology, University of Copenhagen, Copenhagen DK-1350, Denmark.

24 Faculty of Veterinary Medicine, Utrecht University, Utrecht 3584 CM, The Netherlands.
25 Institute of Animal Science and Veterinary Medicine, Shandong Academy of Agricultural Sciences, Shandong Key Lab of Animal Disease Control and Breeding, Jinan 250100, China.
26 College of Animal Science and Technology, Jilin Agricultural University, Changchun 130118, China.
27 School of Life Sciences, University of Nottingham, Nottingham NG7 2RD, United Kingdom.
28 Livestock Genetics Program, International Livestock Research Institute (ILRI), Nairobi 00100, Kenya.
29 Yazhouwan National Laboratory, Sanya 572024, China.
30 Key Laboratory of Livestock Biology, Northwest A&F University, Yangling 712100, China.
# These authors contributed equally
E-mail addresses: o.hanotte@cgiar.org, h.jianlin@cgiar.org, yu.jiang@nwafu.edu.cn, and leichuzhao1118@nwafu.edu.cn.

Supplementary Information

Supplementary Note 1
Whole genome sequencing
Variant discovery and genotyping
Supplementary Note 2
Genetic diversity
Principal component analysis (PCA) and admixture analysis
Neighbor-joining (NJ) and maximum likelihood (ML) phylogenetic trees
Supplementary Note 3
Detection of selection signatures shared by all indicine cattle
Detection of selection signatures in the SAI, EAI, and AFI cattle groups
Supplementary Note 4
Introgression analysis
Genes associated with adaptive introgression
Annotation of gene content in the introgressed segments
Supplementary Note 5
Paternal analysis
Estimation of the divergence time of paternal haplogroups
Whole mitogenome phylogeny
Estimation of the divergence time of maternal haplogroups
Estimation of effective population size and divergence time using autosomal SNPs

Supplementary Figures
Supplementary Fig. 1 Genome-wide nucleotide diversity in different cattle phylogeographic groups obtained by using VCFtools.
Supplementary Fig. 2 Distribution patterns of runs of homozygosity (ROH) in 331 individuals representing 11 taurine and indicine cattle populations.
Supplementary Fig. 3 Mean pairwise F_{ST} values between cattle breeds/populations represented by more than one animal.
Supplementary Fig. 4 Linkage disequilibrium (LD) decay in 29 autosomes of all 495 cattle.
Supplementary Fig. 5 Principal component analysis (PCA) of all 495 cattle, illustrated by PC1 against PC2 (a) and PC1 against PC3 (b).
Supplementary Fig. 6 Principal component analysis (PCA) of all 354 indicine cattle, illustrated by PC1 against PC2 (a) and PC1 against PC3 (b).
Supplementary Fig. 7 Results of admixture analysis of all 495 cattle using 2,996,368 LD-pruned SNPs for *K* from 2 to 8 (plotted in R).
Supplementary Fig. 8 TreeMix relationships among 74 cattle breeds/populations.
Supplementary Fig. 9 Colocalization of selection signatures among and within indicine cattle groups.
Supplementary Fig. 10 Selective sweep analysis comparing the genomes of taurine and indicine cattle.
Supplementary Fig. 11 Selective sweeps on BTA7 (50.52-51.19 Mb).
Supplementary Fig. 12 Selective sweeps on BTA19 (26.40-27.47 Mb), which encompasses the *KIF1C, GP1BA, SPAG7, ENO3, PFN1*, and *CHRNE* genes.
Supplementary Fig. 13 Selective sweeps on BTA1 (81.37-81.70 Mb), which encompasses the *LIPH* gene.
Supplementary Fig. 14 Inferences of population splits and admixtures using TreeMix (a) and OptM results (b).
Supplementary Fig. 15 Allele sharing between indicine cattle and banteng or gaur. **Supplementary Fig. 16** Topologies of introgressed segments of 80 EAI cattle and other bovine species.

Supplementary Fig. 17 Geographic contour map of banteng/gaur introgression proportions in East Asian indicine (EAI) breeds/populations.
Supplementary Fig. 18 Manhattan plots of introgressed segments from banteng (a) and gaur (b) into East Asian indicine (EAI) cattle based on the $U20_{SAI,\ EAI,\ banteng\ or\ gaur}$ (1%, 20%, and 100%) statistic.
Supplementary Fig. 19 Venn diagram of the number of introgressed genes from banteng or gaur into East Asian indicine cattle.
Supplementary Fig. 20 Phylogenetic trees constructed using the haplotype sequences from the BTA1:66690001-66800000, BTA6:69300001-66440000, BTA6:69500001-69600000, and BTA6:69800001-69900000 regions. Haplotypes of East Asian indicine (EAI) cattle that are clustered with banteng (*Bos javanicus*) and gaur (*Bos gaurus*) indicate banteng and gaur introgressions into EAI cattle.
Supplementary Fig. 21 Phylogenetic trees constructed using the haplotype sequences from the BTA6:70100001-70250000, BTA8:11490001-11690000, BTA8:96290001-96400000, and BTA13:62920001-63200000 regions. Haplotypes of East Asian indicine (EAI) cattle that are clustered with banteng (*Bos javanicus*) and gaur (*Bos gaurus*) indicate banteng and gaur introgressions into EAI cattle.
Supplementary Fig. 22 Phylogenetic trees constructed using the haplotype sequences from the BTA13:62710001-62840000, BTA13:63320001-63370000, BTA13:63420001-63590000, and BTA13:63590001-63970000 regions. Haplotypes of East Asian indicine (EAI) cattle that are clustered with banteng (*Bos javanicus*) and gaur (*Bos gaurus*) indicate banteng and gaur introgressions into EAI cattle.
Supplementary Fig. 23 Phylogenetic trees constructed using the haplotype sequences from the BTA13:64090004-64170000, BTA18:60240001-60340000, BTA18:60450001-60500000, and BTA13:60650001-60740000 regions. Haplotypes of East Asian indicine (EAI) cattle that are clustered with banteng (*Bos javanicus*) and gaur (*Bos gaurus*) indicate banteng and gaur introgressions into EAI cattle.
Supplementary Fig. 24 Phylogenetic trees constructed using the haplotype sequences from the BTA19:52090001-52150000, BTA20:540001-600000, BTA24:53920001-54000000, and BTA24:54070001-54170000 regions. Haplotypes of East Asian indicine (EAI) cattle that are clustered with banteng (*Bos javanicus*) and gaur (*Bos gaurus*) indicate banteng and gaur introgressions into EAI cattle.
Supplementary Fig. 25 Phylogenetic trees constructed based on the haplotype sequences from the BTA25:70001-170000, BTA25:190001-300000, and BTA26:11020001-11090000 regions. Haplotypes of East Asian indicine (EAI) cattle that are clustered with banteng (*Bos javanicus*) and gaur (*Bos gaurus*) indicate banteng and gaur introgressions into EAI cattle.
Supplementary Fig. 26 Genetic evidence of introgression of the region including the *ILDR1* gene from banteng and gaur into East Asian indicine (EAI) cattle.
Supplementary Fig. 27 Phylogenetic tree of 309 Y chromosomes based on 1,389 SNPs in the male-specific region of the bovine Y chromosome.
Supplementary Fig. 28 Median-joining (MJ) network of Y chromosomal haplotypes based on 1,389 SNPs in the male-specific region of bovine Y chromosome.
Supplementary Fig. 29 Distribution of Y chromosomal haplogroups in indicine cattle in Africa, South Asia, South China, and North-Central China.
Supplementary Fig. 30 Bayesian tree inferred from 1,389 SNPs in the male-specific regions of the bovine Y chromosome and Bayesian skyline plots.
Supplementary Fig. 31 Phylogenetic tree of complete mitogenomes from indicine cattle.
Supplementary Fig. 32 Bayesian tree inferred from complete mitogenomes of indicine cattle.
Supplementary Fig. 33 Phylogeny of complete indicine cattle mitogenomes using network.
Supplementary Fig. 34 The geographic distribution of maternal haplogroups of indicine cattle in Africa, South Asia, South China, and North-Central China.

Supplementary Fig. 35 Bayesian skyline plots (BSPs) based on mitogenome coding regions.
Supplementary Fig. 36 Coalescence-based inference of the demographic history of indicine cattle based on MSMC2.
Supplementary Fig. 37 Neighbor-joining tree (a) and geographic position of indicine breeds across Southwest China (b).

Supplementary Tables
Supplementary Table 1 Distribution of SNPs in different genomic regions and their types.
Supplementary Table 2 Samples and SNPs information for different analyses.
Supplementary Table 3 ADMIXTURE cross-validation errors from $K = 2$ to $K = 8$.
Supplementary Table 4 Common candidate genomic regions identified in indicine cattle based on the F_{ST}, θ_{π}, and XP-EHH analyses.
Supplementary Table 5 Results from the enrichment analysis of genes under selection in South Asian indicine cattle.
Supplementary Table 6 Common candidate genomic regions identified in East Asian indicine cattle based on the CLR, F_{ST}, θ_{π}, and PBS analyses.
Supplementary Table 7 Results of f_3 statistics performed to detect admixtures among 4 banteng, 2 gaur, 15 South Asian indicine (SAI), and 15 taurine cattle.
Supplementary Table 8 Results from the enrichment analysis of genes introgressed from banteng and gaur into East Asian indicine (EAI) cattle based on the *U20* statistic.
Supplementary Table 9 Top candidate genes associated with adaptive introgression from banteng into East Asian indicine cattle.
Supplementary Table 10 Top candidate genes associated with adaptive introgression from gaur into East Asian indicine cattle.

Supplementary Information

Supplementary Note 1

Whole genome sequencing

We collected and extracted 297 DNA samples from 287 indigenous indicine cattle representing 42 breeds/populations and 10 taurine cattle representing three breeds. We classified the samples according to their geographic origins as follows: four African taurine (AFT), six Eurasian taurine (EUAT), seven Tibetan indicine (TBI), 26 Southeast Asian indicine (SEAI), 57 East Asian indicine (EAI), 85 African indicine (AFI), and 112 South Asian indicine (SAI) cattle. Paired-end libraries were generated for each sample using standard procedures. The average insert size was 500 bp, and the read length was 150 bp. All libraries were sequenced on the Illumina HiSeq X platform to an average raw read sequencing depth of 10×. The average sequencing depth was 11.72×, ranging from 8.20× to 34.00×, per genome. Additional detailed information on the mapping rate and sequencing depth is provided in Supplementary Data 1.

We combined our new data with those from 198 publicly available whole genomes of 39 breeds/populations: six SAI, 26 AFI, six American indicine (AMI), two SEAI, 23 EAI, four Southwest Chinese indicine (SWCI), 15 AFT, eight Tibetan taurine (TBT), 24 Northeast Asia taurine (NEAT), 62 European taurine (EUT), and 22 EUAT cattle. The average sequencing depth was 11.92×, ranging from 8.10× to 34.73×, per genome.

A total of 495 samples from 74 breeds/populations were classified according to their geographic origins as follows: AFT (n = 19), EUT (n = 62), EUAT (n = 28), TBT (n = 8), NEAT (n = 24), AFI (n = 111), SAI (n = 118), SEAI (n = 28), TBI (n = 7), SWCI (n = 4), EAI (n = 80), and AMI (n = 6) cattle (Fig. 1 and Supplementary Data 1). Among them, 317 males had Y-chromosomal variants.

We also used sequencing data of 22 whole genomes from six other bovine species, including two bison, two wisent, five gaur, eight banteng, three yak, and two swamp buffaloes, as outgroups or for introgression analysis. The average sequencing depth was 25.22×, ranging from 7.87× to 37.02×, per genome (Supplementary Data 1).

Variant discovery and genotyping

A total of 495 cattle samples were used for variant discovery. We generated genotype data following the 1000 Bull Genomes Project Run 8 guidelines (http://www.1000bullgenomes.com/) (Supplementary Note 1). We removed low-quality bases and artifact sequences using Trimmommatic v0.39 [1], and all clean reads were mapped to the taurine reference assembly (ARS-UCD1.2) and Btau_5.0.1 Y using BWA-MEM (v0.7.13-r1126) with default parameters [2]. We

then used SAMtools v1.9 [3] to sort bam files. For the mapped reads, potential PCR duplicates were identified using 'MarkDuplicates' of Picard v2.20.2 (http://broadinstitute.github.io/picard). 'BaseRecalibrator' and 'PrintReads' of Genome Analysis Toolkit (GATK, v3.8-1-0-gf15c1c3ef) [4] were used to perform base quality score recalibration (BQSR) with the known variant file (ARS1.2PlusY_BQSR_v3.vcf.gz) provided by the 1000 Bull Genomes Project.

For SNP calling, we created GVCF files using 'HaplotypeCaller' in GATK with the '-ERC GVCF' option. We called SNPs from combined GVCF files using 'GenotypeGVCFs' and 'SelectVariants'. To avoid possible false-positive calls, we used VariantFiltration as recommended, with filtering based on the following criteria: (1) SNP clusters with the '-clusterSize 3' and '-clusterWindowSize 10' options; (2) SNPs with a mean depth (for all samples) < 1/3× and > 3× (×, overall mean sequencing depth across all samples); (3) quality by depth, QD < 2; (4) phred-scaled variant quality score, QUAL < 30; (5) strand odds ratio, SOR > 3; (6) Fisher strand, FS > 60; (7) mapping quality, MQ < 40; (8) mapping quality rank sum test, MQRankSum< -12.5; and (9) read position rank sum test, ReadPosRankSum < -8. We then filtered out nonbiallelic SNPs and SNPs with missing genotype rates > 0.1. A total of autosomal 67,162,108 SNPs were identified (Supplementary Table 1). The whole-genome sequencing data from six other bovine species were processed in the same way. We genotyped the combined set of 495 cattle samples and 22 samples of six other bovine species, and then extracted the 67,162,108 SNPs. After filtering out the non-biallelic SNPs, 67,145,163 autosomal SNPs were obtained. The two final SNP genotyping datasets were phased and imputed using BEAGLE v4.0 [5] with default parameters and filtered by DR2 < 0.9 (Supplementary Table 2). The remaining SNPs were annotated according to their positions using SnpEff v4.3 [6]. We also summarize the samples and SNPs used for different analyses in Supplementary Table 2.

Supplementary Note 2

Genetic diversity

The genome-wide nucleotide diversity of different cattle geographic groups was estimated with VCFtools v0.1.16 [7] (Supplementary Fig. 1). Genetic distances between breeds/populations were calculated with the F_{ST} estimates and runs of homozygosity (ROH) were analyzed using PLINK v1.9 [8, 9] (Supplementary Figs. 2 and 3). The VCF file containing 67,162,108 SNPs was converted into PLINK format with VCFtools v0.1.16 [7]. We filtered samples with a mapping depth < 10 × or 3× genome coverage < 90% and used 331 individuals for ROH analysis. We used phased and imputed SNPs to detect ROH using PLINK v1.9 [6]. The final parameters were set to a minimum

length of 100 kb, a scanning window size of 100 SNPs, a minimum density threshold of 200 SNPs, a large gap of 1,000 kb, a maximum number of heterozygous SNPs in the scanning window of 1, and a scanning window threshold level of 0.05. These settings yielded expected number (maximum number was 3,259) and total length (maximum length was 1,138,710 Mb) of ROH (Supplementary Fig. 2).

Principal component analysis (PCA) and admixture analysis

For PCA and admixture analyses, we first filtered out SNPs with a minor allele frequency (MAF) < 0.01 and performed linkage disequilibrium (LD)-based pruning for the genotype data using the --indep-pairwise 50 10 0.1 option of PLINK v1.9 [6] according to the results LD of linkage disequilibrium (LD) decay analysis (Supplementary Fig. 4). PCA was performed with LD-pruned SNPs for all 495 cattle and 354 indicine cattle using EIGENSOFT v4.2 [10]. The Tracy–Widom test was used to determine the significance level of eigenvectors. The results were plotted with *ggplot2* in R v4.1.0 [11] (Supplementary Figs. 5 and 6). We used ADMIXTURE v1.3.0 [12] to quantify the genome-wide admixture among modern cattle populations. ADMIXTURE was run for each possible ancestry number (K = 2 to 8), which was used to determine the optimal ancestry number (K) (Supplementary Table 3 and Supplementary Fig. 7).

Neighbor-joining (NJ) and maximum likelihood (ML) phylogenetic trees

To identify relationships among cattle, the 67,162,108 autosomal SNPs were used to construct an NJ tree with PLINK v1.9 based on the matrix of pairwise genetic distances [6] (Fig. 1). FigTree v1.4.3 (http://tree.bio.ed.ac.uk/software/figtree/) was used to visualize the NJ tree. Then, we inferred a population-level phylogeny using the ML approach implemented in TreeMix [13]. We performed LD-based pruning for the genotype data of 495 cattle and three yak using the --indep-pairwise 50 10 0.1 option in PLINK v1.9 [6]. A total of 15,390,936 LD-pruned SNPs and the "-global -root yak" parameter were used to generate the ML tree (Supplementary Fig. 8).

Supplementary Note 3

Detection of selection signatures shared by all indicine cattle

To reveal the genetic changes that may be affected by selection, we combined SAI, EAI, and AFI cattle into a single indicine gene pool. We screened for genomic regions with genetic diversity (θ_π ratio), F_{ST}, and cross-population extended haplotype homozygosity (XP-EHH) outliers between taurine (EUT, EUAT, NEAT, TBT, and AFT, n = 141) and all indicine cattle (SAI, EAI, and AFI, n =

309) using VCFtools v0.1.16 [7]. For the XP-EHH selection scan, our test statistic was the average normalized XP-EHH score calculated using selscan v1.1 with default settings [14]. The θ_π ratio, F_{ST}, and average normalized XP-EHH score were estimated for 50 kb windows with 20 kb steps. After performing all tests, windows with P values less than 0.005 (Z test) were considered to show significant signals. P values were estimated based on Z-transformed values using the standard normal distribution and were further corrected for multiple testing by using the Benjamin–Hochberg false discovery rate (FDR) method. The candidate genes selected in all indicine cattle were defined as the genes with overlapping signals for any two of these three selection methods (θ_π ratio, F_{ST}, and XP-EHH).

We obtained 156 windows from the three methods and these windows harbored 117 candidate genes (Table 1, Supplementary Table 4, Supplementary Figs. 9 and 10). Some significant selection signatures identified by these three methods were plotted along with the haplotype structure based on the SNPs in the selective regions using a small sliding window (10 kb) to visualize the top signals (Supplementary Figs. 11-13).

Detection of selection signatures in the SAI, EAI, and AFI cattle groups

CLR and *iHS* were employed to detect the selection signatures in the SAI (n = 118), EAI (n = 80), and AFI (n = 111) genomes. The CLR was calculated for 50 kb windows with 20 kb steps using SweepFinder2 [15]. The command used to perform this scan was "SweepFinder2 -lu GridFile FreqFile SpectFile OutFile". We combined the F_{ST} outliners between the target group and the other two indicine groups. P values (Z test) were calculated for the CLR and F_{ST} windows and those less than 0.005 were considered as candidate regions (Fig. 2, Supplementary Fig. 9, Supplementary Tables 5 and 6, and Supplementary Data 2 and 3). The *iHS* was implemented in selscan v1.1 [14], and the proportion of SNPs with $|iHS| \geq 2$ was calculated in windows of 50 kb with steps of 20 kb. To perform *iHS* and CLR computation, information on the ancestral and derived allele state is needed for each SNP. In our analysis, the ancestral allele was defined as the allele fixed in the swamp buffalo that was included in the genotype call set, and the ambiguous SNPs were discarded. To capture potential genes that were specifically selected in each indicine group, we also calculated the F_{ST} between the target group and the two other indicine groups. P values were calculated for the CLR, $|iHS|$, and F_{ST} windows, and the overlapping windows with $P < 0.005$ (Z test) for each method were considered as candidate signatures of selection.

Considering that the EAI genomes were affected by banteng/gaur introgression, we used the population branch statistic (PBS) [16] in 50 kb windows with 20 kb steps to scan for genomic regions highly differentiated in EAI relative to SAI, AFI, and banteng (n = 4) genomes. Significant genomic

regions were identified by $P < 0.005$. In addition, F_{ST} and θ_π methods were used to generate a line chart of the top signals (Supplementary Table 6 and Fig. 3).

Gene set enrichment analyses were performed by determining the enriched Gene Ontology (GO) categories and Kyoto Encyclopedia of Genes and Genomes (KEGG) pathways with KOBAS v3.0 [17]. To provide an initial overview of the overrepresented groups of genes and to test their reliability, we performed GO category and KEGG pathway enrichment analyses with KOBAS v3.0 [17] using different lists of selected genes. Only pathways or annotations with a Bonferroni-corrected $P < 0.05$ were retained (Supplementary Table 5).

Supplementary Note 4

Introgression analysis

To assess the direction of gene flow, the *D* statistics were calculated in ADMIXTOOLS v6.0 [18]. If there was no gene flow, we would expect the *D*-statistic to be zero (null hypothesis). The *D* statistic method considers the tree topology [[[PopA(W), PopB(X)], PopC(Y)], buffalo(Z)], where the buffalo represents the outgroup, Y is the admixed population, and W and X are the test populations. The *D* statistic method counts the "ABBA" sites, where W and Z share the outgroup allele (A) while X and Y share the derived allele (B), as well as the "BABA" sites, where W and Y share the derived allele while X and Z share the outgroup allele. Admixture between Y and either of the test populations creates a significant difference between the ABBA and BABA counts, with a *Z* score > 3.0 for gene flow between W and Y or ≤ -3.0 for gene flow between X and Y. Standard error is obtained by a block JackKnife approach and *Z* scores at statistical significance. The *D* statistic was used to select pure SAI cattle, taurine cattle, banteng, and gaur for introgression analysis (Supplementary Data 4 and 5). For SAI cattle, we used the three tree topologies of *D* (SAI individual, SAI individual; taurine cattle, buffalo), *D* (SAI individual, SAI individual; banteng individual, buffalo), and *D* (SAI individual, SAI individual, gaur, buffalo) to select the SAI samples without any gene flow from taurine cattle, banteng or gaur. For taurine cattle, we used three tree topologies of *D* (taurine individual, taurine individual; SAI, buffalo), *D* (taurine individual, taurine individual; banteng individual, buffalo), and *D* (taurine individual, taurine individual; gaur individual, buffalo) to select taurine samples without any gene flow from SAI cattle, banteng or gaur. For banteng, we used two tree topologies of *D* (banteng individual, banteng individual; SAI individual, buffalo) and *D* (banteng individual, banteng individual; taurine individual, buffalo) to select banteng samples without any gene flow from taurine or SAI cattle. For gaur, we used two tree topologies of *D* (gaur individual, gaur individual; SAI individual, buffalo) and *D* (gaur individual, gaur individual; taurine individual, buffalo) to select gaur samples without any gene flow from

taurine or SAI cattle. We finally selected a panel of 15 pure SAI cattle, 15 taurine cattle, 4 banteng, and 2 gaur samples with a $|Z \text{ score}| < 3$ for RFMix analysis, *D* statistic, *U*20, and *U*50 statistical calculations.

To detect gene flow between the populations of the panel (4 banteng, 2 gaur, 15 taurine, and 15 SAI cattle), we also used the three-population test (f_3 statistics) and calculated their corresponding normalized value (*Z* scores) at the population level in the "qp3Pop" program implemented in ADMIXTOOLS v6.0 [18] (Supplementary Table 7). The f_3 statistic considers the population triplet (A, B, and C), where C is the test (target) population and A and B are reference (source) populations. If the *Z* score ($Z \leq -3.0$) is significantly negative, the test population C has admixture from both reference populations of A and B.

TreeMix [13], the *D* statistic [19], and RFMix v2.02 [20] were used to test the introgression hypothesis (Supplementary Figs. 14-17). We used RFMix to further validate banteng or gaur introgression into individual EAI cattle. The same panel of 15 SAI cattle, 15 taurine cattle, 4 banteng, and 2 gaur samples were included as references.

Local ancestry was inferred using RFMix v2.02 [20] in the phased data with the parameters recommended in the documentation and the four populations in the panel were set as references for different ancestries: taurine ancestry, indicine ancestry, banteng ancestry, and gaur ancestry. The introgressed fragments were defined by the following criteria: (1) fragments that shared ≥ 2 haplotypes and ≥ 2 samples and (2) ≥ 30 SNPs of introgressed fragments.

We calculated the probability of banteng/gaur introgressed tracts in EAI cattle due to incomplete lineage sorting (ILS) [21]. We let *r* be the recombination rate per generation per base pair in indicine cattle, *m* be the length of the introgressed tracts, and *t* be the homologous tracts that are shared by banteng/gaur and cattle branches since their divergence [22]. The expected length of a shared ancestral sequence was $L = 1/(r \times t) = 206.52$ bp. The probability of a length of at least *m* was 1-GammaCDF (m, $shape = 2$, $r = 1/L$), in which GammaCDF was the gamma distribution function. We calculated the length of the introgressed tracts (m) and filtered for those that were too short (fragments $< L$) to be confidently introgressed. We applied the probability of ILS < 0.05 to filter short introgressed segments in the RFMix results (Supplementary Data 6 and 7). We estimated the proportion of an EAI genome that was introgressed from banteng/gaur using the total introgressed length divided by the taurine cattle reference genome (ARS_UCD1.2) length. We used IQ-TREE v1.6.6 [23] to construct phylogenetic trees for the introgressed regions from banteng/gaur. A total of 80 tree topologies were constructed by the ML method. Each tree was constructed using the merged sequences of introgressed segments of each EAI cattle according to the RFMix results and homologous sequences of eight other bovine species. The ML phylogeny of EAI (red samples)

cattle supported the introgression of banteng/gaur in 80 EAI genomes. DensiTree was used to merge and visualize the trees [24] (Supplementary Fig. 16).

Genes associated with adaptive introgression

We used the statistic $U20_{SAI,\ EAI,\ banteng\ or\ gaur}$ (1%, 20%, and 100%) [25] to detect genomic regions associated with adaptive introgression, which was equal to the number of SNPs within a genomic window where a particular allele was fixed (frequency of 100%) in banteng/gaur but at a frequency less than 1% in SAI cattle or greater than 20% in EAI cattle. We denoted SAI, EAI, and banteng/gaur as the "outgroup", "target", and "source" panels, respectively (Supplementary Table 8 and Supplementary Figs. 18 and 19). We also used a higher cutoff for the frequency of the banteng/gaur allele in EAI cattle ($U50_{SAI,\ EAI,\ banteng\ or\ gaur}$ (1%, 50%, 100%)) to detect uniquely shared high-frequency banteng alleles (Supplementary Tables 9 and 10 and Supplementary Figs. 20-26). Following this treatment, 70 adaptive genes in 32 candidate regions were shortlisted (Fig. 4) and 23 regions were then validated by phylogenetic analysis using 5 SAI cattle, 5 taurine cattle, 2 wisent, 2 bison, 3 yak, 4 banteng, 2 gaur, 2 swamp buffaloes, and 80 EAI samples (Supplementary Figs. 20-25).

For the analysis of the introgressed region of BTA25 (0.21-0.26 Mb), we also used a gayal sample and an ancient kouprey sample to detect its origin. The coverages of gayal and kouprey were 17.32× and 1.40×, respectively. Due to the hybrid origin of gayal and low coverage of the kouprey genome, we did not include them in the analysis of general introgression of gayal and kouprey to East Asian indicine cattle. The publicly available sequences were downloaded from China National GeneBank (CNGB) with the following project accession numbers: CRX165997 (gayal, YD4) and PRJNA764746 (kouprey).

Annotation of gene content in the introgressed segments

To provide an initial overview of the overrepresented groups of genes and to test their reliability, we performed GO and KEGG pathway enrichment analyses with KOBAS v3.0 [17] using different introgressed gene lists as detected by $U20_{SAI,\ EAI,\ banteng\ and\ gaur}$ (1%, 20%, and 100%) and $U50_{SAI,\ EAI,\ banteng\ or\ gaur}$ (1%, 50%, and 100%). Only pathways or annotations with a Bonferroni-corrected $P <$ 0.01 were retained (Supplementary Table 8).

Supplementary Note 5

Paternal analysis

We selected the Xd regions of the bovine male-specific region (MSY) (from 2.5 to 3.9 Mb (Xd1) and from 42.2 to 43.3 Mb (Xd2)) (GCF_000003205.7) [26] for all analyses in this section.

For 316 male samples, we obtained sequencing depths of 4.08-19.40× for Y chromosomes with an average of 4.73×. We called genotypes as described in Supplementary Note 1. Only the SNPs called in the MSY region that met the following criteria were retained: (1) present in at least two males but not in females and (2) no heterozygous site. We also removed SNPs with missing genotypes in 10% of all male samples. Final SNPs were filtered out based on an allele count > 4. After performing quality control and filtering, we extracted 309 samples and 1,389 SNPs to construct a haplogroup tree. Phylogenetic trees were then inferred using both ML and Bayesian methods. An ML analysis was conducted with MEGA v7 [27](Supplementary Fig. 27). A Bayesian phylogenetic tree was constructed using BEAST v2.6.0 [28] (Supplementary Fig. 27).

We also constructed a median-joining (MJ) network using NETWORK v5.0.1.1 [29] (Supplementary Fig. 28). To further explore the migration of Y3A haplotypes in China, we extracted the indicine Y haplotypes carried by 26 individuals of 10 taurindicine breeds from the North-Central region of China reported in previous studies. We genotyped these 26 individuals based on the 1,389 SNPs. The results showed that the Y3A haplotypes migrated from the southern to the northern regions of China (Supplementary Data 8 and Supplementary Fig. 29).

Estimation of the divergence time of paternal haplogroups

Molecular dating of haplogroup splitting for 309 sequences was implemented using BEAST v2.6.0 [28]. A maximum clade credibility tree was generated using a 10% burn-in with TreeAnnotator as part of the BEAST set of programs and drawn with FigTree v1.4.3 (http://tree.bio.ed.ac.uk/software/figtree/). The BSP of the indicine haplogroup Y3 and its sub-haplogroup Y3A3 were generated using the following parameters: HKY substitution model with gamma-distributed rates, a log-normal relaxed clock, coalescent Bayesian skyline analysis, a mutation rate per generation of 1.26×10^{-8}, and a generation time of 6 years [30]. We ran 100,000,000 iterations for Y3 and 50,000,000 iterations for Y3A3, with samples collected every 5,000 steps, and visualized the resulting BSP with Tracer v1.7.1 [31]. BSPs were created using the *ggplot2* R package in R v4.1.0 [11]. The node age of Y3A3 (5.57 ky) was used as the only a priori parameter (Supplementary Fig. 30).

Whole mitogenome phylogeny

We extracted mitochondrial bam files of 354 indicine cattle. BAM alignments were converted to FASTQ format, and mitogenomes were assembled using Mapping Iterative Assembler v1.0 (MIA) [32]. We first selected all indicine cattle in our dataset for mitogenome analysis, and then we selected only mitogenomes that were successfully assembled by MIA software and filtered mitogenomes

with a gap length > 1 bp, which resulted in 329 complete indicine cattle mitogenomes (Supplementary Data 1).

These 329 mitogenomes were aligned to 18 bovine reference mitogenomes, including the P, Q, T1-T5, and I1-I2 haplogroups. The best substitution models were determined using ModelGenerator v0.85 [33]. Phylogenetic relationships were inferred using RAxML v8.2.9 [34] with the following parameters: -f a -x 123 -p 23 -# 100 -k -m GTRGAMMA. The final tree topology was visualized using FigTree v1.4.3 (Supplementary Fig. 31). A Bayesian phylogenetic tree was constructed using BEAST v2.6.0 [28] (Supplementary Fig. 32). The MJ network was constructed using NETWORK v5.0.1.1 [29] (Supplementary Fig. 33). To further explore the migration of the I1a haplogroup in East Asia, we extracted 74 complete mitogenomes of 13 taurindicine breeds from the north-central region of China reported in previous studies (Supplementary Data 8 and Supplementary Fig. 34).

Estimation of the divergence times of maternal haplogroups

The divergence times between the major haplogroups of indicine cattle mtDNA were inferred with BEAST v2.6.0 [28]. A Bayesian tree was constructed using the mtDNA coding regions in the 329 indicine cattle mitogenomes and 18 references (V00654, AY676856, EU177859, EU177839, AB074964, DQ124372, NC_006853, EU177863, EU177862, EU177841, DQ124389, GU985279, EU177867, EU177866, FJ971080, EU177842, AF492350, and EU177868).

Bayesian age estimates of haplogroups and BSPs were generated for four different datasets with mtDNA coding regions: a complete dataset containing all 347 mitogenomes; a dataset encompassing the 223 indicine cattle mitogenomes, two indicine references, and two sequences belonging to clade P; a dataset encompassing the 86 I1a mitogenomes; and a dataset including all 106 taurine mitogenomes and 18 mitogenomes belonging to the reference haplogroups of clades P, Q, and T. We used the HKY substitution model (with gamma-distributed rates) with the log-normal relaxed clock. We applied an evolutionary rate of $2.043 \pm 0.099 \times 10^{-8}$ base substitutions per nucleotide per year [35]. We ran 10 independent BEAST runs with the chain length established at 20,000,000 iterations, samples collected every 5,000 MCMC steps and a 10% burn-in. The runs were then combined using the LogCombiner utility within the BEAST package [36] by applying another 10% burn-in. A maximum clade credibility tree was drawn with FigTree v1.4.3 (http://tree.bio.ed.ac.uk/software/figtree/). BSP data were obtained with Tracer v1.7.1 using default parameters [31] and then converted to a graph using a generation time of six years [37]. The BSP was created using the *ggplot2* R package in R v4.1.0 (Supplementary Fig. 35).

Estimation of effective population size and divergence time using autosomal SNPs

The multiple sequential coalescent Markovian model 2 (MSMC2) method [38] was used to model the population history of the three core indicine groups (EAI, SAI and AFI) and to infer historical changes in their effective population size and population separation. We applied this method to all groups with two deep-coverage (>14 ×) individuals per group. All sample sets of filtered variant calls were used for phasing and imputation in Beagle v4.1 [5] with default parameters. The DR2 value in the INFO column of the "phase.vcf" file was used to filter SNP sites, and SNPs with DR2 > 0.9 were retained. We also applied genome masking as recommended in the documentation of the software. For the calculation of effective population size, the parameter of MSMC2 was set to "msmc2 -t 10 -p 1*2+25*1+1*2 -I 0, 1, 2, 3" and "msmc2 -t 10 -p 1*2+25*1+1*2 -I 4, 5, 6, 7". For the calculation of population separation, the parameter of MSMC2 was set to "msmc2 -t 8 -P 0, 0, 0, 0, 1, 1, 1, 1 -s -p 1*2+25*1+1*2". For effective population size inference, two individuals (4 phased haplotypes) from each group were used. A time scale in generation time of $g = 6$ and a mutation rate per generation of $\mu_g = 1.26 \times 10^{-8}$ were used.

The samples (coverage) used in this analysis were as follows: EAI, WZ28A (14.23) and WZ23A (14.04); SAI, Har03 (34.73) and Sha3b (20.96); AFI, RAY26 (17.49) and RAY06 (17.01); and Tibetan taurine cattle, Xizang22 (26.28) and Xizang7 (24.56). For both taurine and indicine cattle, a common substantial decrease in *N*e was detected at 20-30 kya, which likely reflected the major climatic change at the end of the Last Glacial Maximum, predating cattle domestication. We defined the estimated divergence time between a pair of groups as the first time point at which the cross-coalescence rate was equal to or greater than 0.5. For the range of divergence times, we used the first time point at which the cross-coalescence rate was equal to or greater than 0.25 or 0.75. The relative cross-coalescence analysis suggested a decrease to 0.5 between EAI and SAI cattle at ~10.3 kya (0.25 to 0.75 range = 6.6 to 15.1 kya), a decrease to 0.5 between AFI and SAI cattle at ~11.8 kya (0.25 to 0.75 range = 5.1 to 16.7 kya), and a decrease to 0.5 between EAI and AFI cattle at ~20.1 kya (0.25 to 0.75 range = 9.7 to 38.0 kya). We calculated a decrease in the cross-coalescence rate between taurine and indicine (SAI and EAI) cattle to 0.5 at ~251.5-301.2 kya (Supplementary Fig. 36), consistent with the results of our previous study (201 to 213 kya) [39].

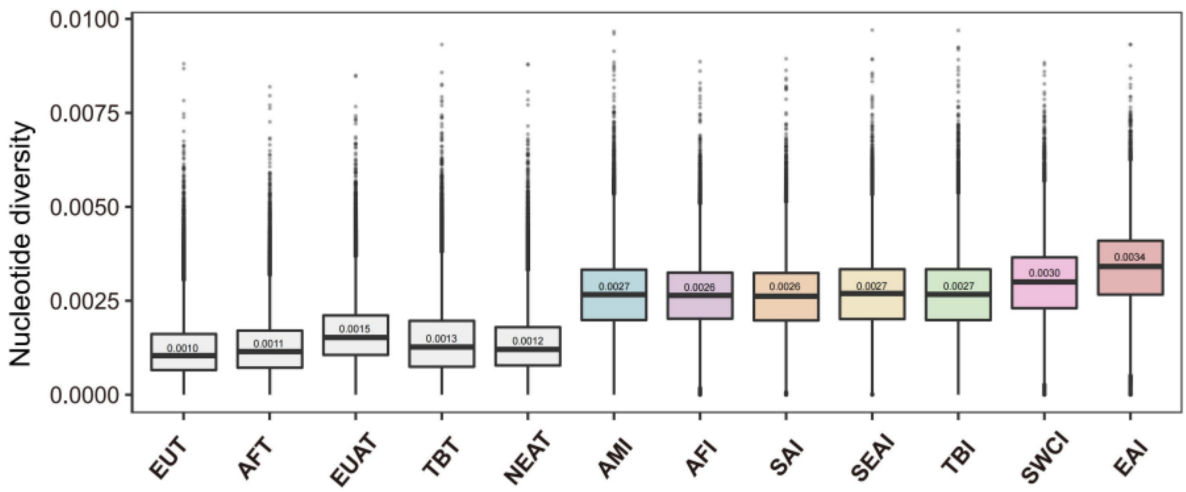

Supplementary Fig. 1 Genome-wide nucleotide diversity in different cattle phylogeographic groups obtained by using VCFtools. The horizontal line inside the box corresponds to the median of the distribution, and the upper and lower parts of the box are the first and third quartiles, respectively. Data points outside the whiskers can be considered as outliers. (EUT, European taurine; AFT, African taurine; EUAT, Eurasian taurine; TBT, Tibetan taurine; NEAT, Northeast Asian taurine; AMI, American indicine; AFI, African indicine; SAI, South Asian indicine; SEAI, Southeast Asian indicine; TBI, Tibetan indicine; SWCI, Southwest Chinese indicine; and EAI, East Asian indicine cattle).

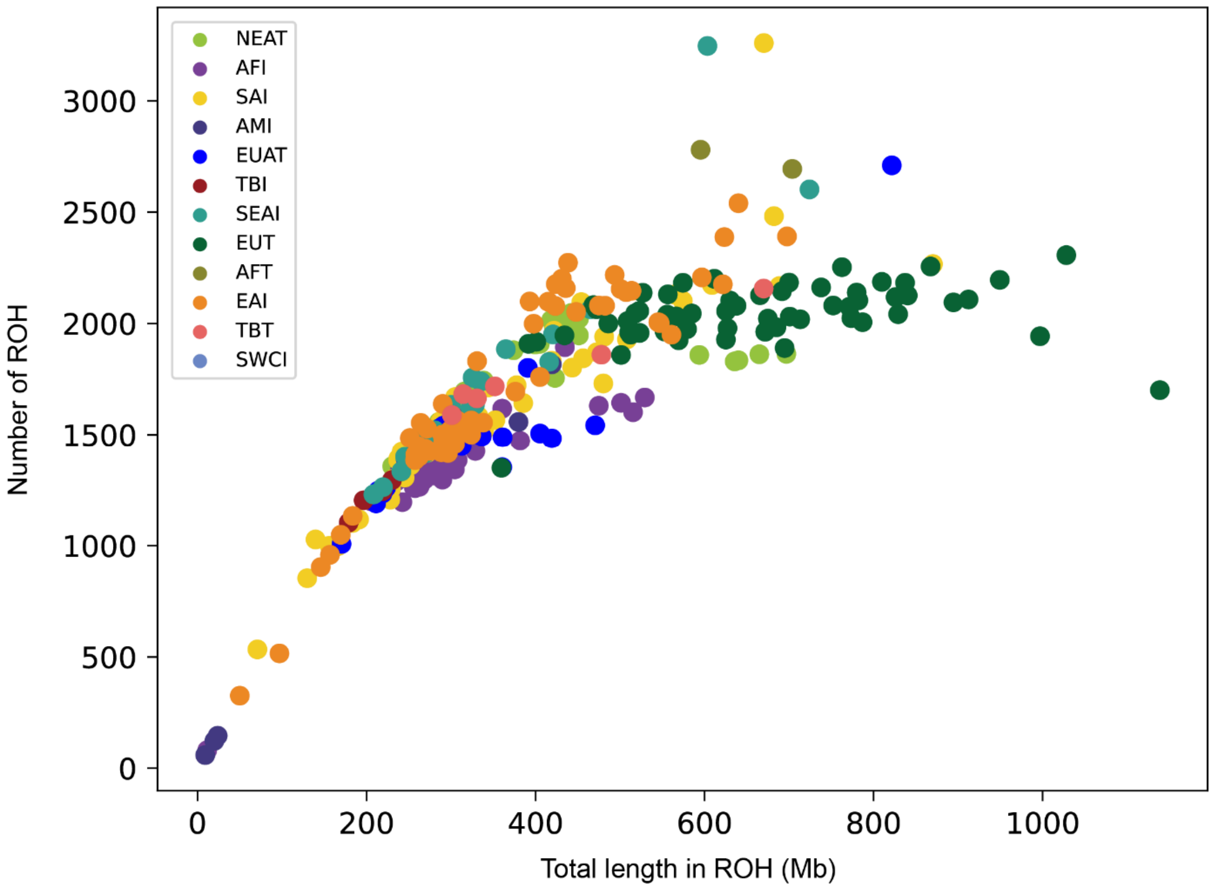

Supplementary Fig. 2 Distribution pattern of runs of homozygosity (ROH) in 331 individuals representing 11 taurine and indicine populations. A total of 65,336,403 SNPs were used for ROH analysis using PLINK. The final parameters were set to a minimum length of 100 kb, a scanning window size of 100 SNPs, a minimum density threshold of 200 SNPs, a large gap of 1000 kb, a maximum number of heterozygous SNPs in the scanning window of 1, and a scanning window threshold level of 0.05. The results show that these settings yield the expected number (maximum number was 3,259) and total length (maximum length is 1,138,710 Mb) of ROH. (AFT, African taurine; EUT, European taurine; EUAT, Eurasian taurine; TBT, Tibetan taurine; NEAT, Northeast Asian taurine; AFI, African indicine; SAI, South Asian indicine; SEAI, Southeast Asian indicine; TBI, Tibetan indicine; SWCI, Southwest Chinese indicine; EAI, East Asian indicine; and AMI, American indicine cattle).

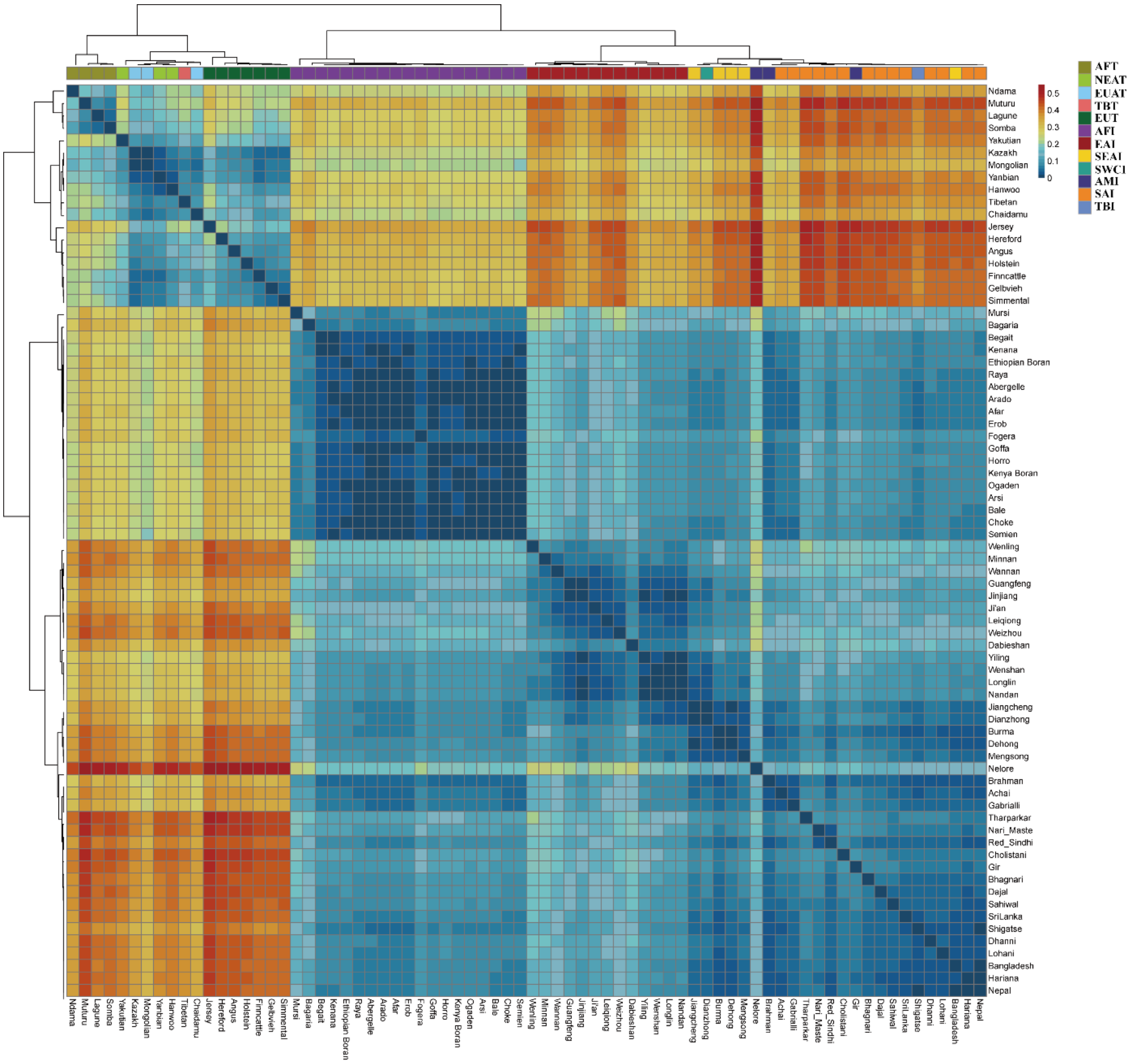

Supplementary Fig. 3 Mean pairwise F_{ST} values between cattle breeds/populations represented by more than one animal. A total of 484 samples and 65,160,804 SNPs were used. (AFT, African taurine; EUT, European taurine; EUAT, Eurasian taurine; TBT, Tibetan taurine; NEAT, Northeast Asian taurine; AFI, African indicine; SAI, South Asian indicine; SEAI, Southeast Asian indicine; TBI, Tibetan indicine; SWCI, Southwest Chinese indicine; EAI, East Asian indicine; and AMI, American indicine cattle).

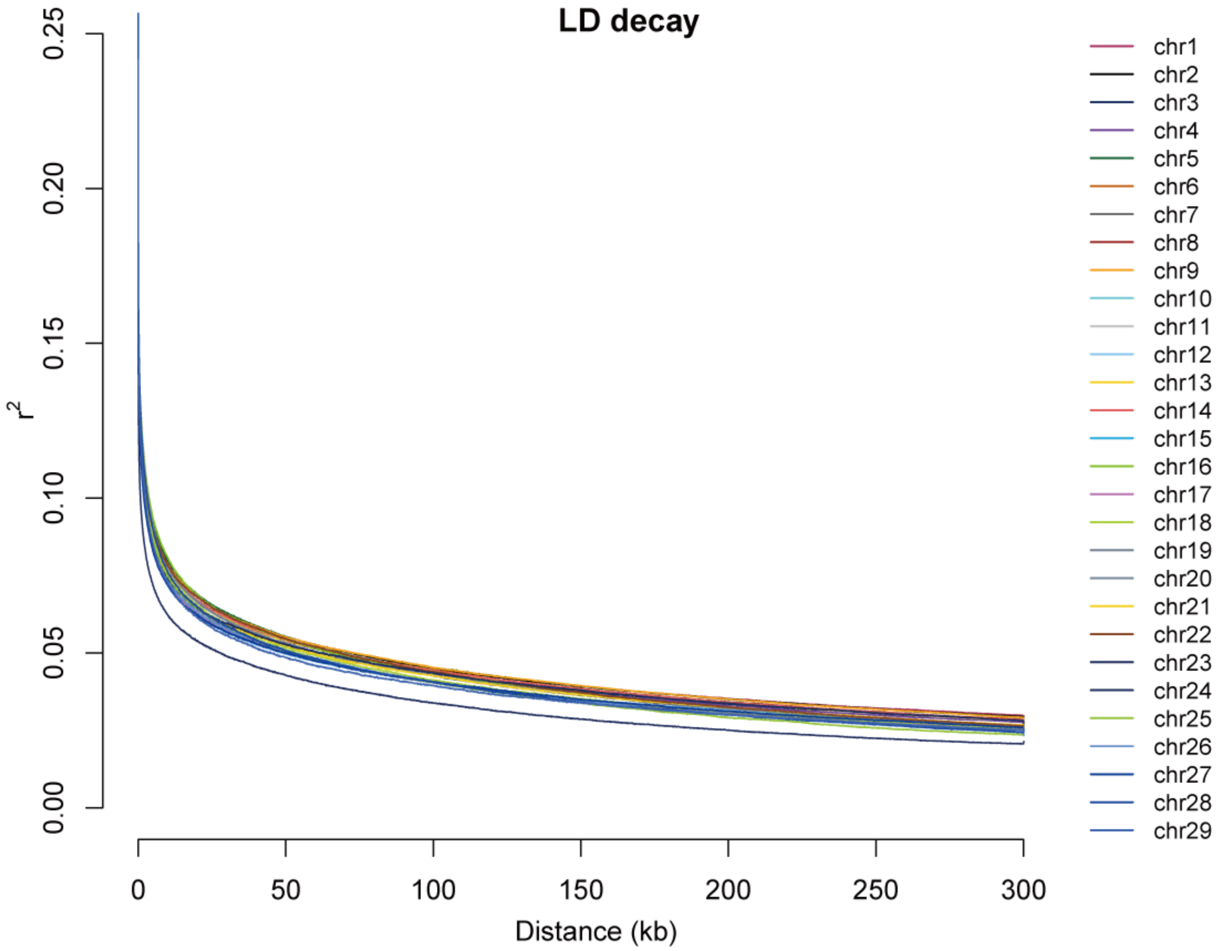

Supplementary Fig. 4 Linkage disequilibrium (LD) decay in 29 autosomes of all 495 cattle. The half LD decay distance is 0.13.

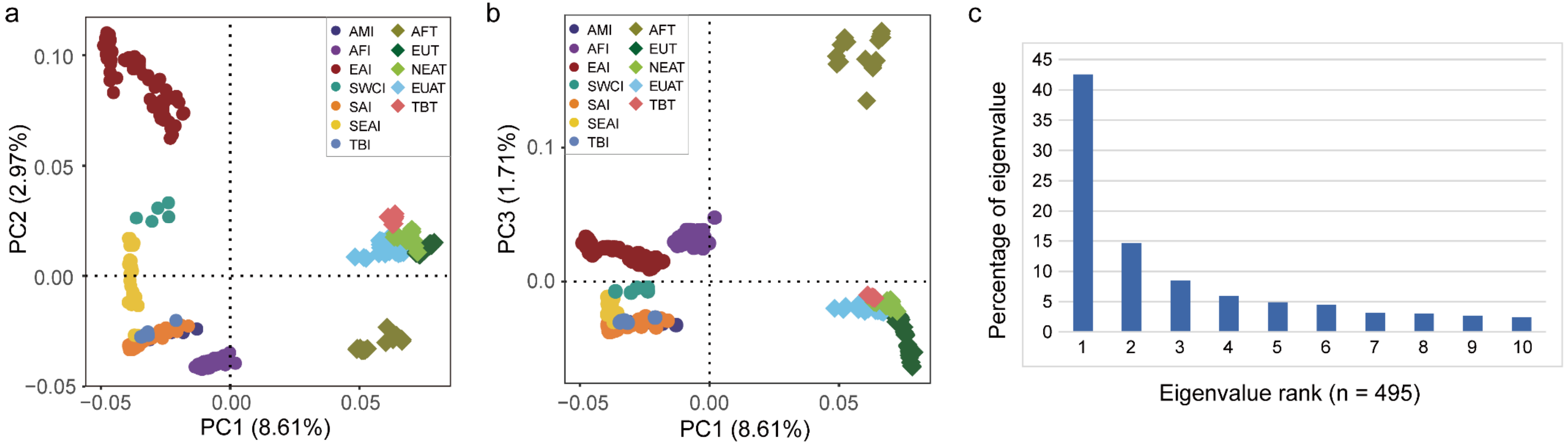

Supplementary Fig. 5 Principal component analysis (PCA) of all 495 cattle, illustrated by PC1 against PC2 (a) and PC1 against PC3 (b). Colors reflect the geographic regions of sampling. PCA percentage of eigenvalues of all 495 cattle (c). A total of 2,996,368 LD-pruned SNPs were used for PCA. (AFT, African taurine; EUT, European taurine; EUAT, Eurasian taurine; TBT, Tibetan taurine; NEAT, Northeast Asian taurine; AFI, African indicine; SAI, South Asian indicine; SEAI, Southeast Asian indicine; TBI, Tibetan indicine; SWCI, Southwest Chinese indicine; EAI, East Asian indicine; and AMI, American indicine cattle).

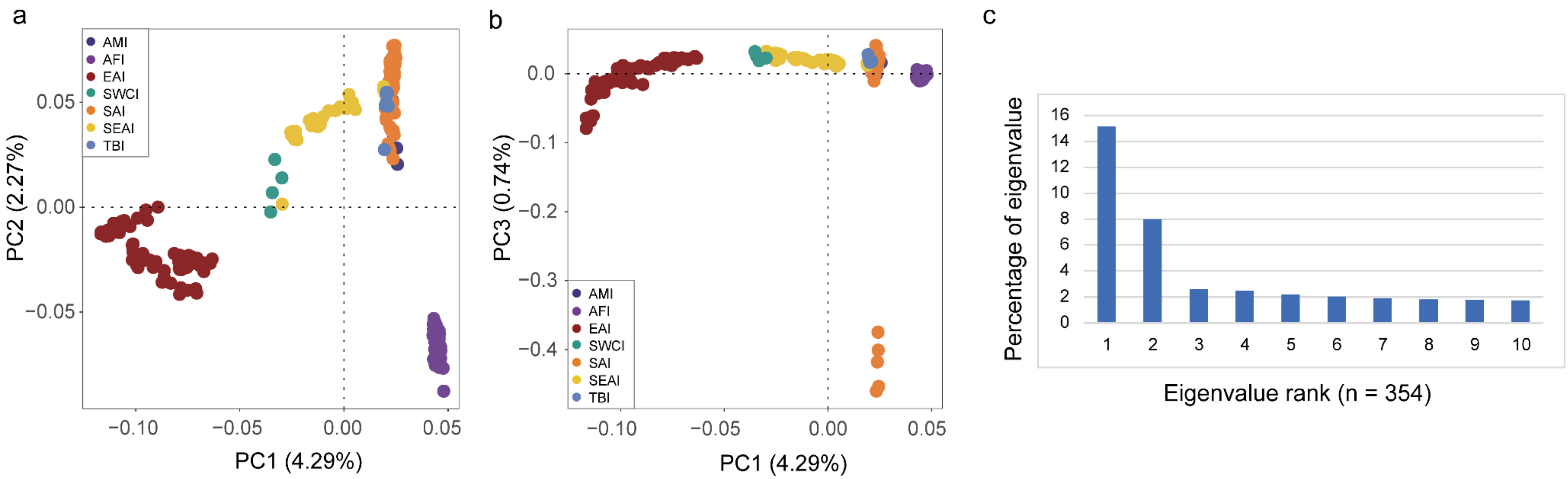

Supplementary Fig. 6 Principal component analysis (PCA) of all 354 indicine cattle, illustrated by PC1 against PC2 (a) and PC1 against PC3 (b). Colors reflect the geographic regions of sampling. PCA percentage of eigenvalues of all 354 indicine cattle (c). A total of 2,565,770 LD-pruned SNPs were used for PCA. (AFI, African indicine; SAI, South Asian indicine; SEAI, Southeast Asian indicine; TBI, Tibetan indicine; SWCI, Southwest Chinese indicine; EAI, East Asian indicine; and AMI, American indicine cattle).

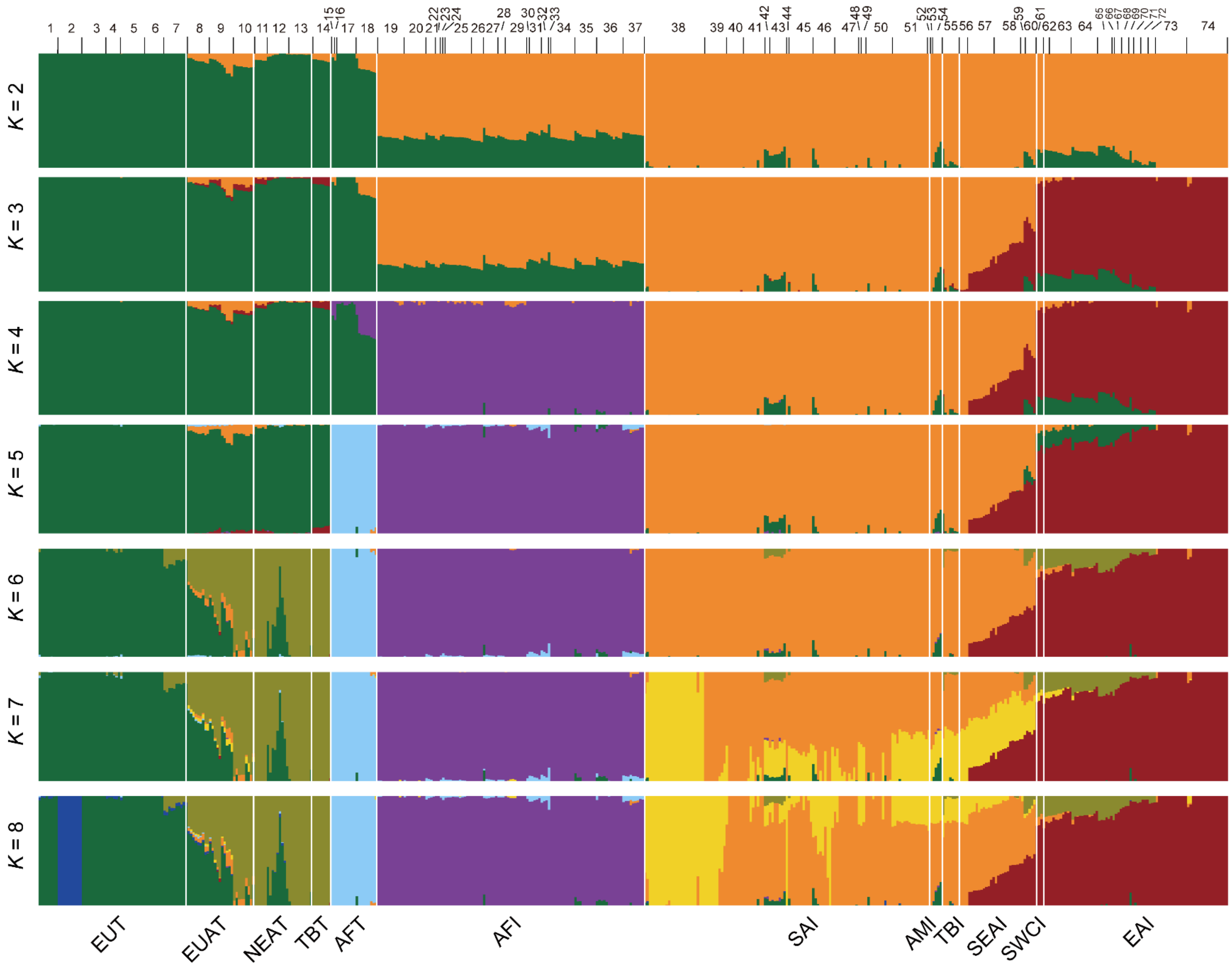
K = 2
K = 3
K = 4
K = 5
K = 6
K = 7
K = 8
EUT
EUAT
NEAT
TBT
AFT
AFI
SAI
AMI
TBI
SEAI
SWCI
EAI

Supplementary Fig. 7 Results of admixture analysis of all 495 cattle using 2,996,368 LD-pruned SNPs for *K* from 2 to 8 (plotted in R). The 74 cattle breeds/populations are listed from left to right as follows: (1) Simmental, (2) Jersey, (3) Angus, (4) Gelbvieh, (5) Hereford, (6) Holstein, (7) Finncattle, (8) Kazakh, (9) Mongolian, (10) Chaidamu, (11) Yakutian, (12) Yanbian, (13) Hanwoo, (14) Tibetan taurine cattle, (15) Somba, (16) Lagune, (17) Muturu, (18) Ndama, (19) Abergelle, (20) Arado, (21) Arsi, (22) Afar, (23) Bale, (24) Bagaria, (25) Begait, (26) Ethiopian, (27) Semien, (28) Choke, (29) Erob, (30) Fogera, (31) Goffa, (32) Horro, (33) Mursi, (34) Raya, (35) Ogaden, (36) Kenya Boran, (37) Ethiopian Boran, (38) Kenana, (39) SriLanka, (40) Cholistani, (41) Tharparkar, (42) Bhagnari, (43) Gabrialli, (44) Achai, (45) Nari Master, (46) Dhanni, (47) Red Sindhi, (48) Dajal, (49) Hariana, (50) Lohani, (51) Sahiwal, (52) Nepal, (53) Nelore, (54) Gir, (55) Brahman, (56) Shigatse, (57) Bangladesh, (58) Burma, (59) Dehong, (60) Jiangcheng, (61) Dianzhong, (62) Wenshan, (63) Longlin, (64) Nandan, (65) Yiling, (66) Dabieshan, (67) Jinjiang, (68) Guangfeng, (69) Wenling, (70) Minnan, (71) Ji'an, (72) Wannan, (73) Leiqiong, and (74) Weizhou. There is strong support for the divergence of taurine from indicine ancestries at *K* = 2 first. The population subdivision at *K* = 3 then separates East Asian indicine (EAI) cattle from South Asian indicine (SAI) cattle. Southeast Asian indicine (SEAI) and Southwest Chinese indicine (SWCI) cattle are composed of crosses with SAI and EAI genotypes. African indicine (AFI) cattle is composed of crosses with SAI and AFT (African taurine) genotypes. At *K* = 4, AFI cattle is further separated from SAI cattle. Population subdivision at *K* = 6 produces three different taurine cattle groups: European taurine (EUT), East Asian taurine (EAT), and AFT ancestries. (EUAT, Eurasian taurine; TBT, Tibetan taurine; NEAT, Northeast Asian taurine; TBI, Tibetan indicine; and AMI, American indicine cattle).

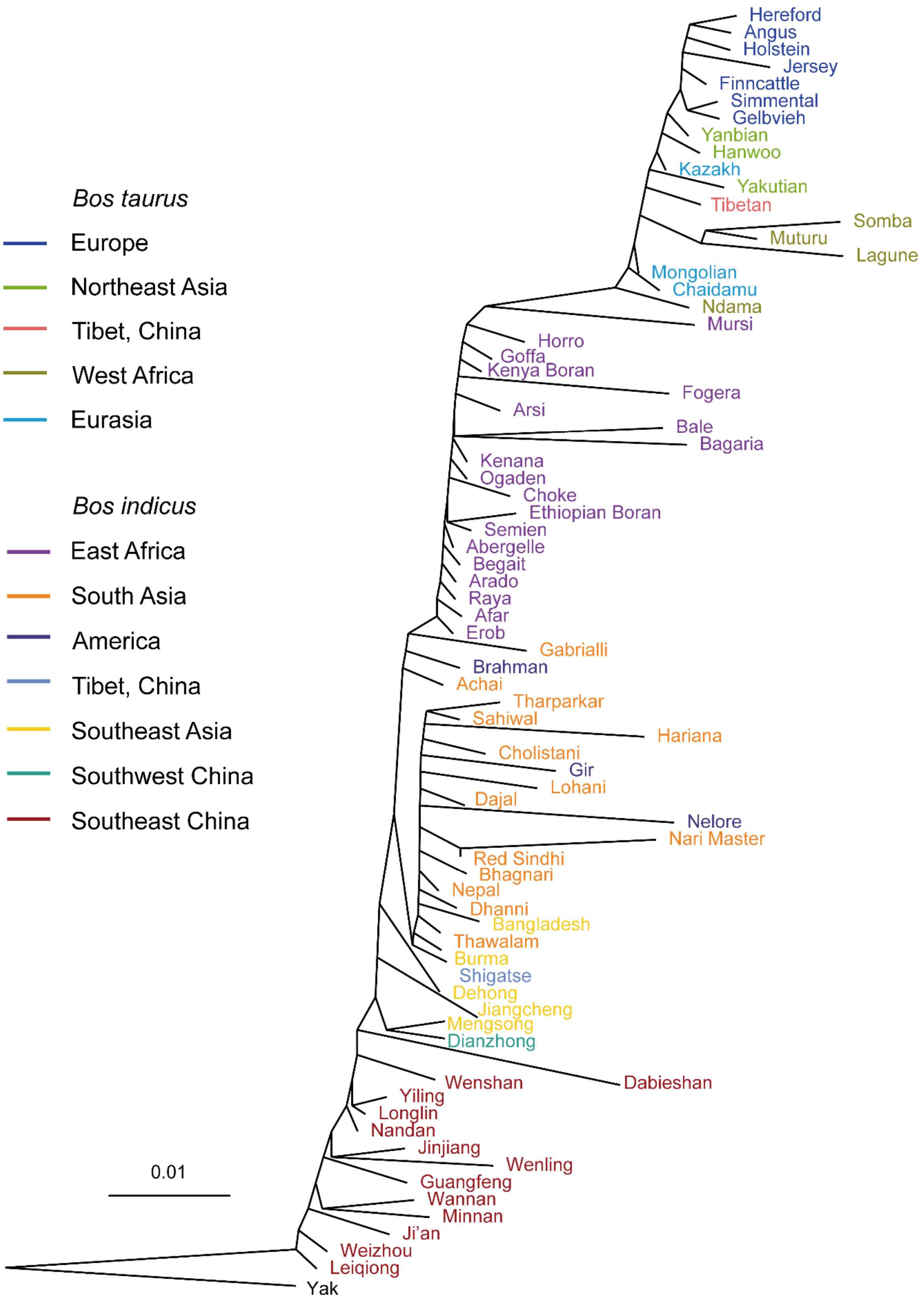

Supplementary Fig. 8 TreeMix relationships among 74 cattle breeds/populations. A total of 15,228,801 SNPs and the "-global -root yak" parameter were used to generate the maximum likelihood phylogenetic tree.

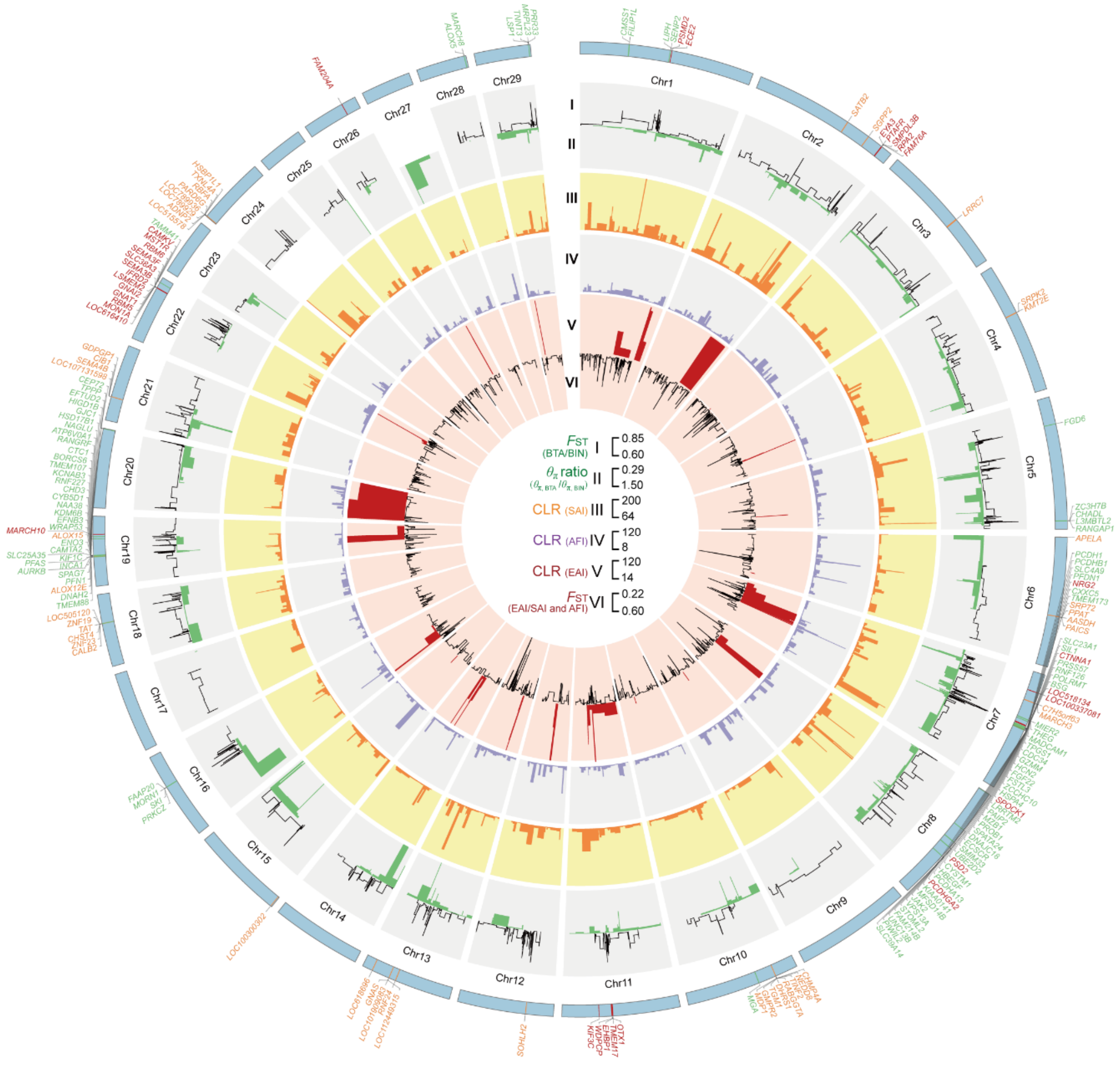

Supplementary Fig. 9 Colocalization of selection signatures among and within indicine cattle groups. From outer to inner circles, the signatures of selection (corresponding to Z test, $P < 0.005$) from each statistic are shown in the following order: I, green outer circle: F_{ST} between indicine and taurine cattle; II, green inner circle: θ_π ratio of taurine to indicine cattle; III, yellow circle: composite likelihood ratio (CLR) in the South Asian indicine (SAI) group; IV, purple circle: CLR in the African indicine (AFI) group; V, outer orange circle: CLR in the East Asian indicine (EAI) group; VI, inner orange circle: F_{ST} between the EAI group and both SAI and AFI groups. Statistical significance ($P < 0.005$) of signals is based on the Z test. The ranges of the plots are indicated in the middle of the circles. Candidate genes identified for all indicine cattle are indicated in green, for the SAI group in orange, and for the EAI group in red. The θ_π ratio and CLR scores are truncated at 1.50 and 200, respectively. (BTA, *Bos taurus*; BIN, *Bos indicus*). P values were estimated based on Z-transformed values using the standard normal distribution, and were further corrected by multiple testing using the Benjamini–Hochberg false discovery rate (FDR) method.

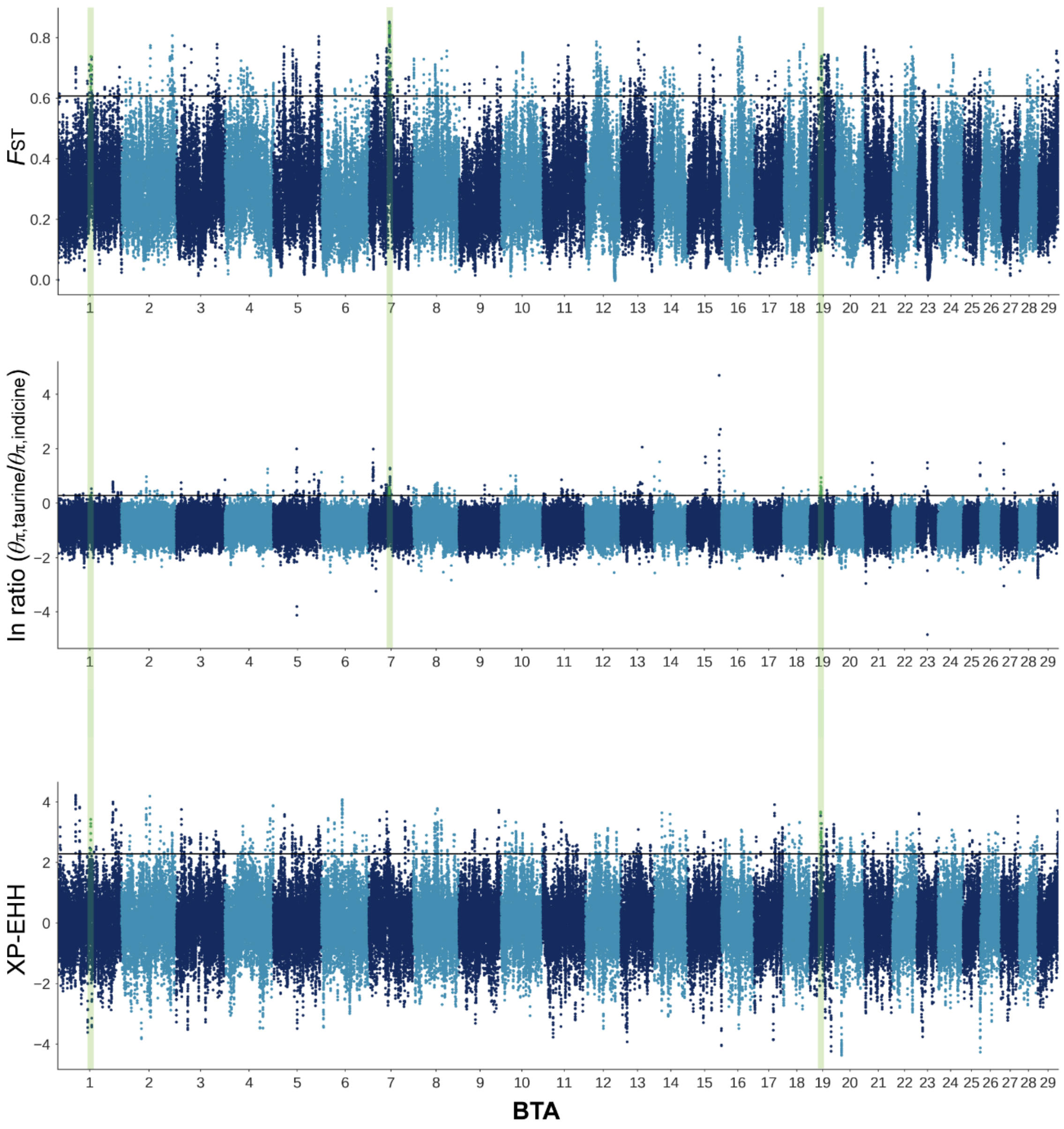

Supplementary Fig. 10 Selective sweep analysis comparing the genomes of taurine and indicine cattle. Pairwise fixation index (F_{ST}) (top panel), π ln ratio (middle panel), and normalized XP-EHH scores (bottom panel) calculated between taurine and indicine cattle in 50 kb windows with 20 kb steps across all autosomes. The black horizontal lines indicate the significance thresholds (corresponding to Z test $P < 0.005$, where $F_{ST} > 0.608$, θ_π ratio > 0.288, and XP-EHH > 2.29) used for extracting outliers. The three loci with the highest F_{ST} values are highlighted by a shaded green column on BTA1, 7, and 19. *P* values were estimated based on *Z*-transformed values using the standard normal distribution, and were further corrected by multiple testing using the Benjamini–Hochberg false discovery rate (FDR) method.

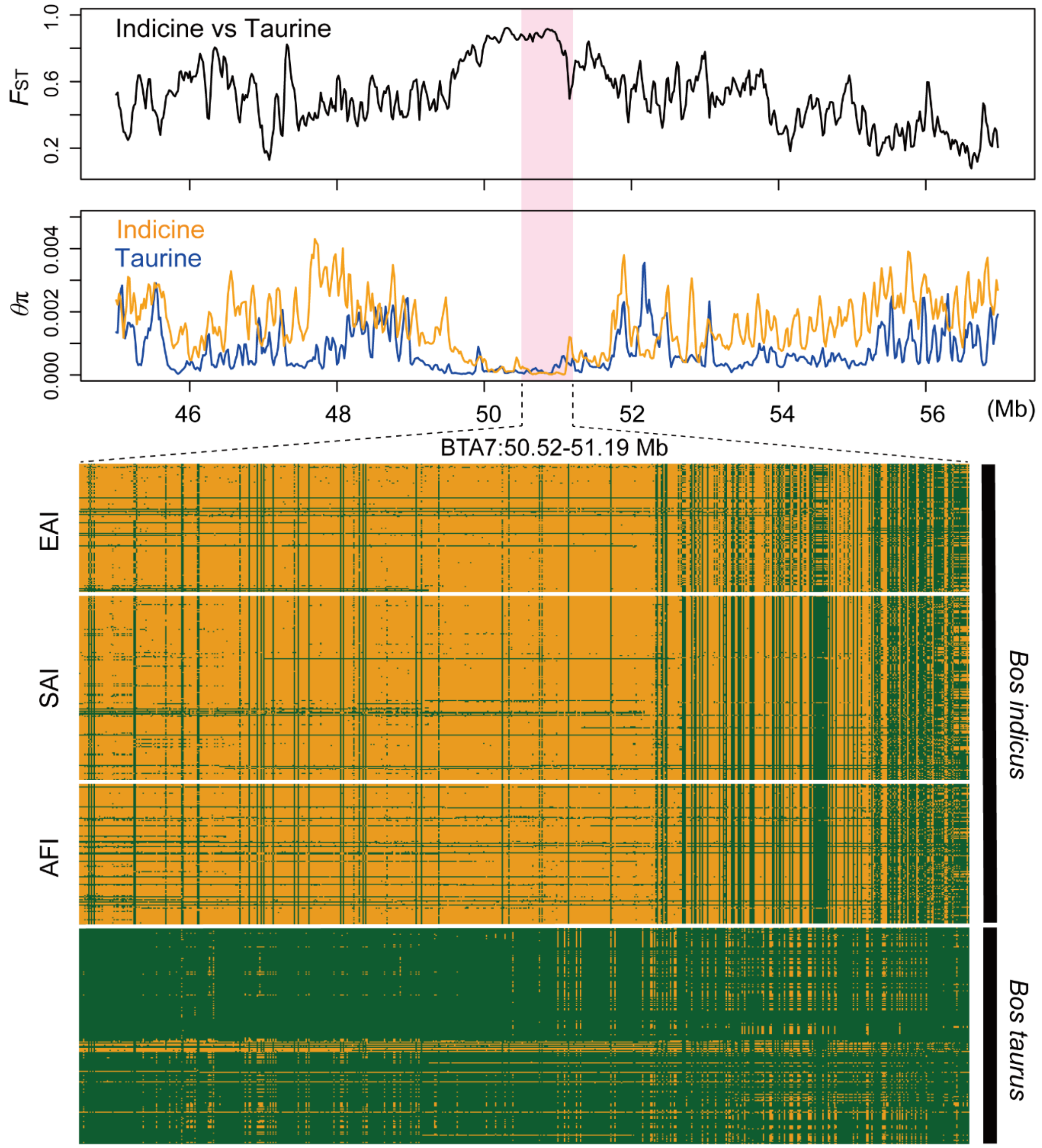

Supplementary Fig. 11 Selective sweeps on BTA7 (50.52-51.19 Mb). F_{ST} and nucleotide diversity (θ_{π}) values are plotted using a 10 kb sliding window. Plot of haplotype structure of SNPs in the selected regions in East Asian indicine (EAI), South Asian indicine (SAI), African indicine (AFI), and taurine (*Bos taurus*) (bottom) cattle, in which alleles with yellow represent reference alleles and those with green represent alternate alleles, while green columns represent the reference alleles, and yellow columns represent the alternative alleles.

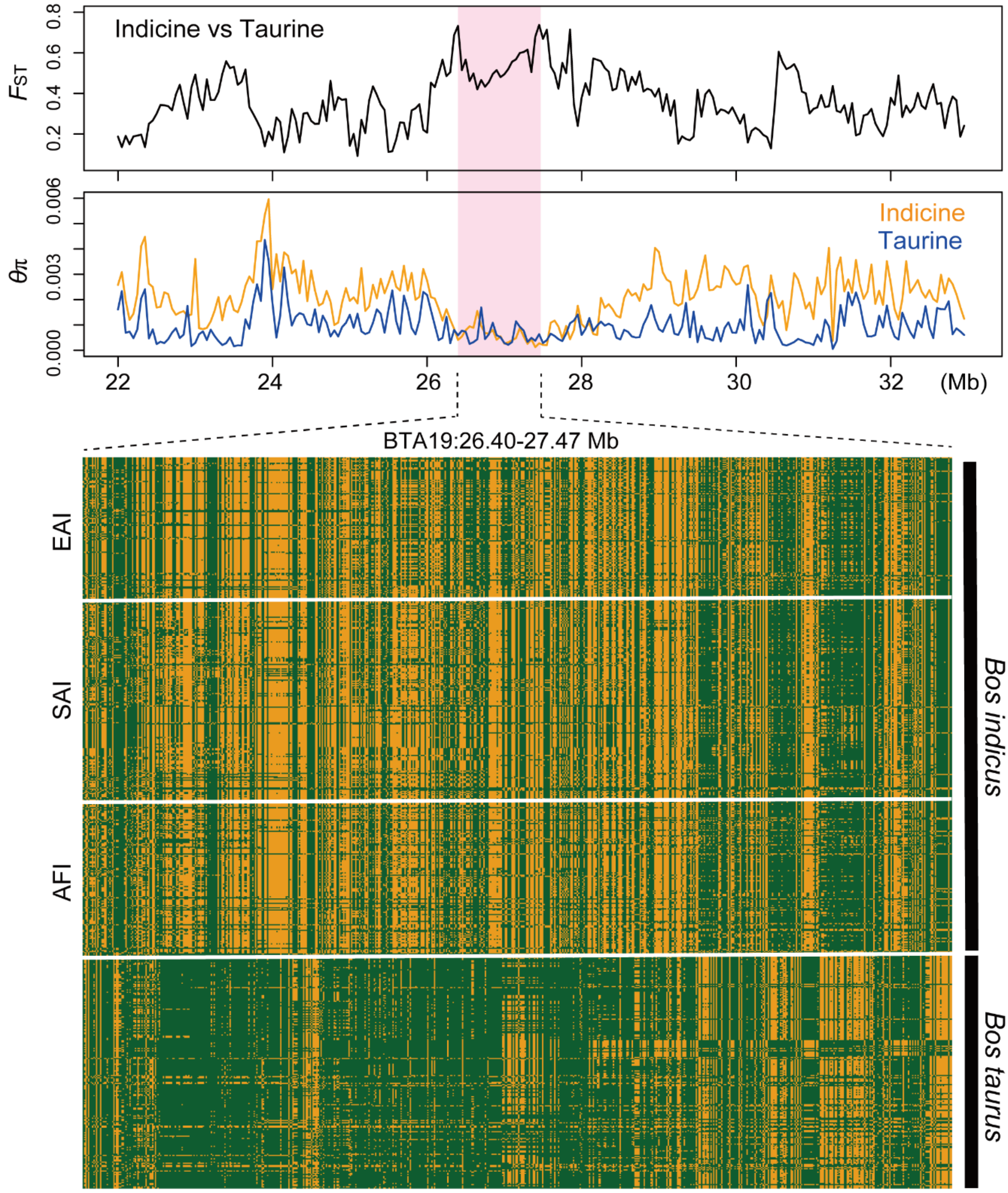

Supplementary Fig. 12 Selective sweeps on BTA19 (26.40-27.47 Mb), which encompasses the *KIF1C, GP1BA, SPAG7, ENO3, PFN1*, and *CHRNE* genes. F_{ST} and nucleotide diversity (θ_π) values are plotted using a 10 kb sliding window. Plot of haplotype structure of SNPs in the selected regions in East Asian indicine (EAI), South Asian indicine (SAI), African indicine (AFI), and taurine (*Bos taurus*) (bottom) cattle, in which green columns represent the reference alleles and yellow columns represent the alternative alleles.

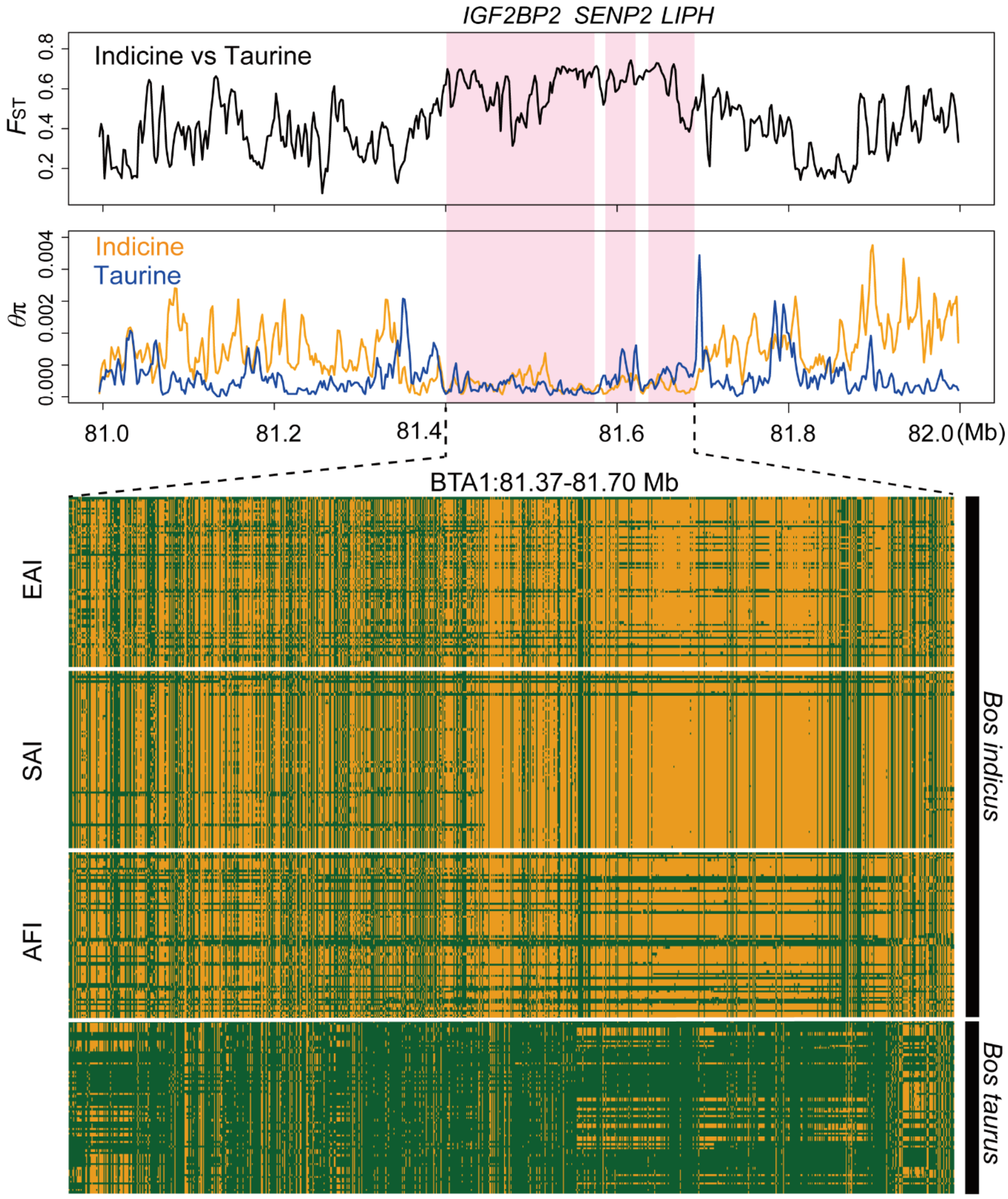

Supplementary Fig. 13 Selective sweeps on BTA1 (81.37-81.70 Mb), which encompasses the *LIPH* gene. F_{ST} and nucleotide diversity (θ_π) values are plotted using a 10-kb sliding window. Plot of haplotype structure of SNPs around the *LIPH* gene in East Asian indicine (EAI), South Asian indicine (SAI), African indicine (AFI), and taurine (*Bos taurus*) (bottom) cattle, in which green columns represent the reference alleles and yellow columns represent the alternative alleles.

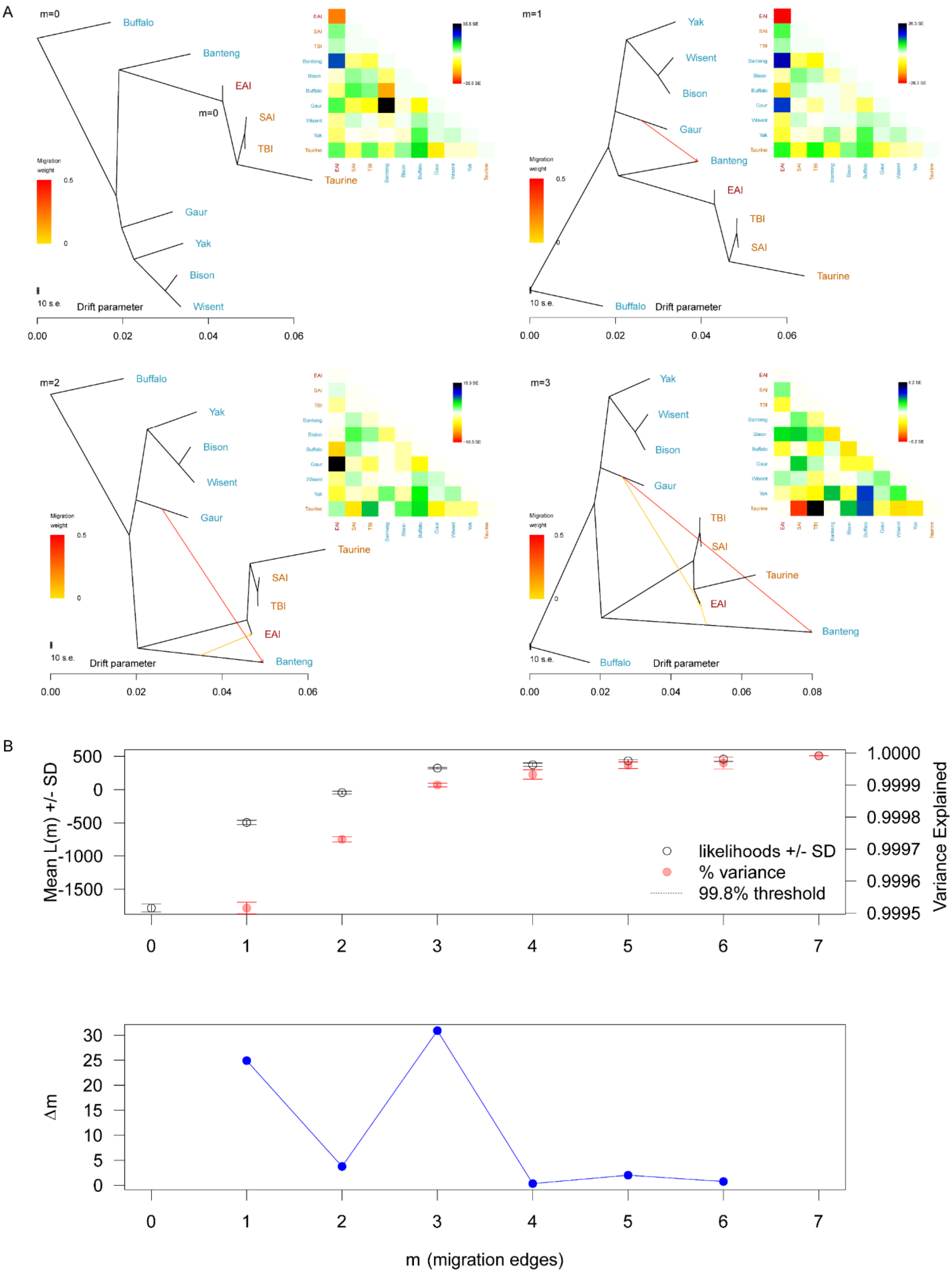

Supplementary Fig. 14 Inferences of population splits and admixture using TreeMix (a) and OptM results (b). The output produced by OptM for an empirical dataset of East Asian indicine (EAI) and six other bovine species/three taurine/indicine cattle groups. The second-order rate of change (Δm) across values of m. The peak in Δm at 3 edges. (TBI, Tibetan indicine; SAI, South Asian indicine; and AFI, African indicine cattle).

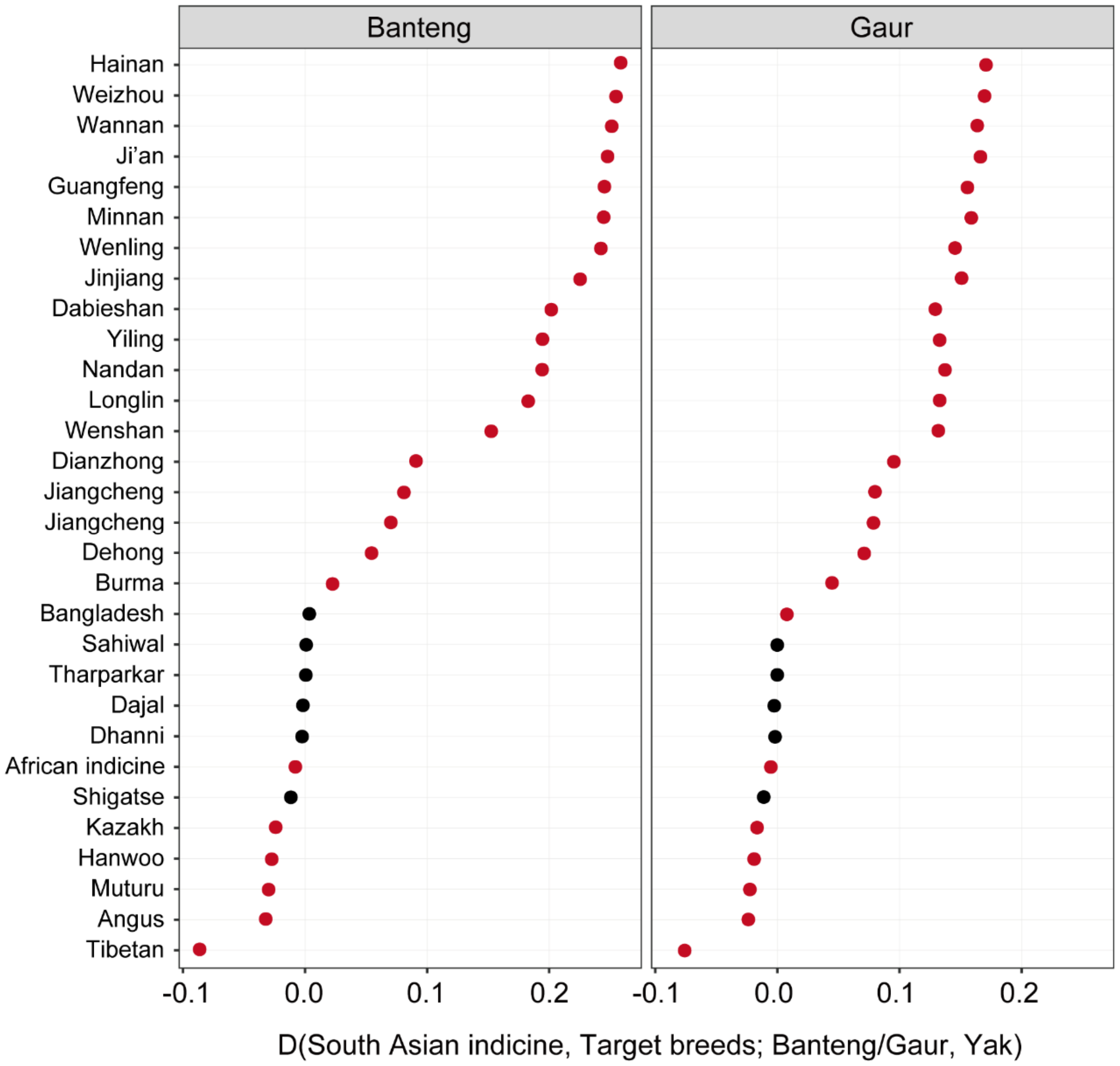

Supplementary Fig. 15 Allele sharing between indicine cattle and banteng or gaur. Statistically significant results, defined as $|Z \text{ scores}| \geq 3$, are marked with a red dot. Negative values are obtained if banteng and gaur more closely related to South Asian indicine cattle, while positive values are obtained if they are more closely related to target breeds.

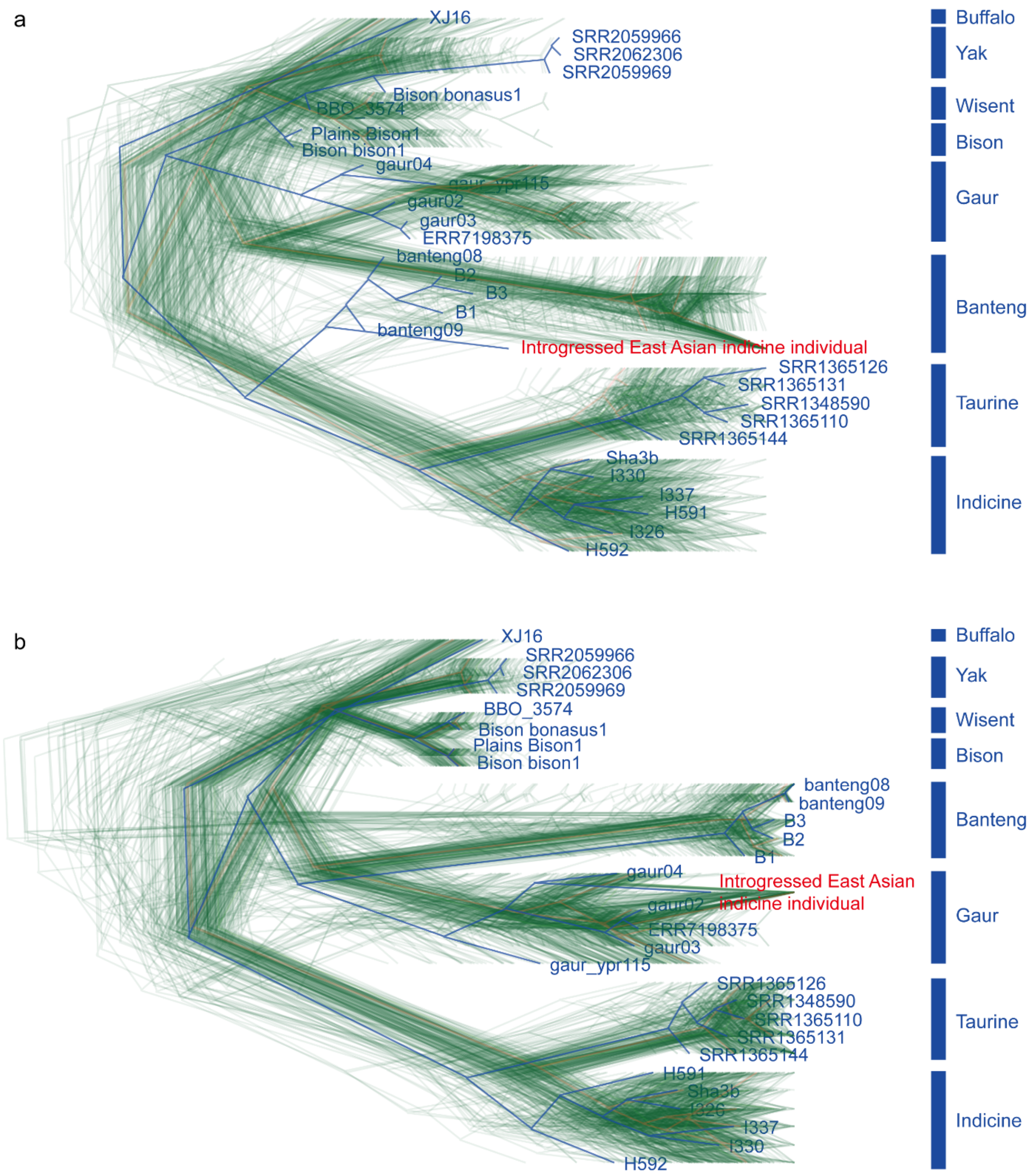

Supplementary Fig. 16 Topologies of introgressed segments of 80 East Asian indicine (EAI) cattle and other bovine species. A total of 80 topologies were constructed by maximum likelihood (ML) method. Each tree was constructed using the merged sequences of the introgressed segments of each EAI cattle according to the RFmix results and homologous sequences of the other eight bovine species. ML phylogeny of EAI cattle (red samples) supported the introgression from banteng (a) or gaur (b) into 80 EAI cattle.

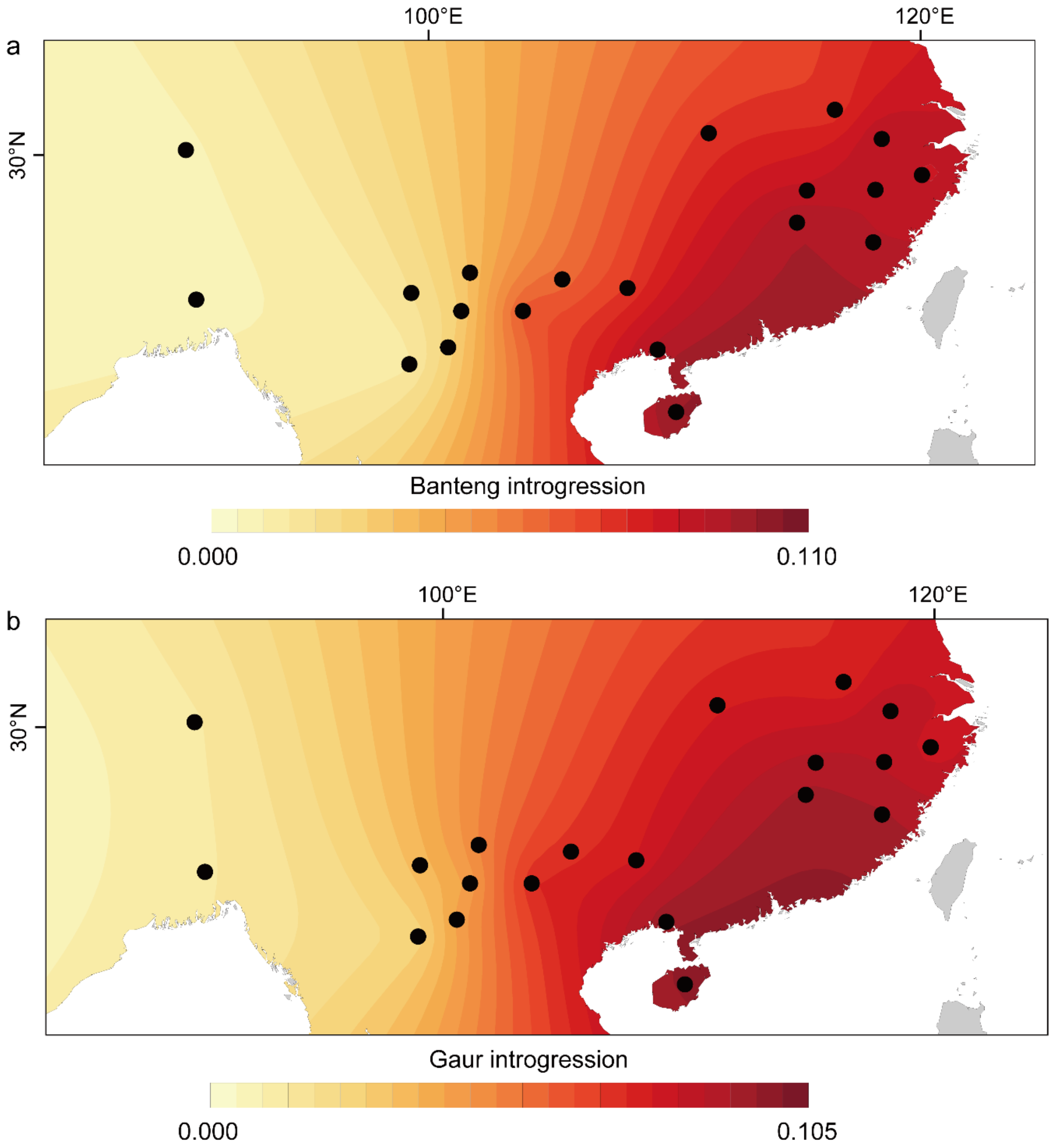

Supplementary Fig. 17 Geographic contour map of banteng/gaur introgression proportions in East Asian indicine (EAI) breeds/populations. The proportions of banteng (a) or gaur (b) introgressions were calculated by RFMix. These results provide compelling evidence supporting the hypothesis of a significant genetic contribution from banteng and gaur to modern EAI cattle. EAI cattle in the southeastern coast of China show the highest level of banteng and gaur ancestries. The map was drawn using the R package v4.1.0.

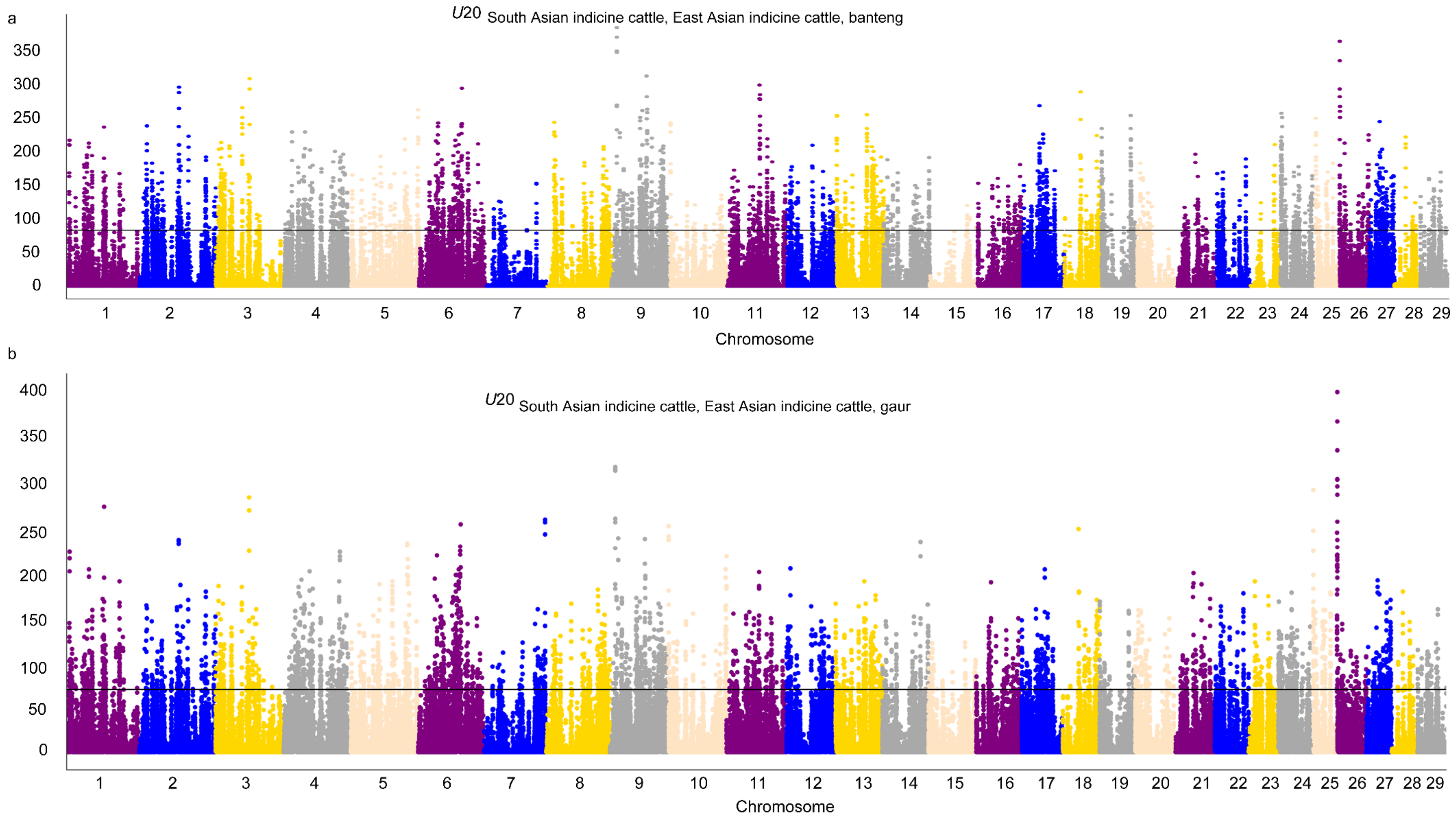

Supplementary Fig. 18 Manhattan plots of introgressed segments from banteng (a) or gaur (b) into East Asian indicine (EAI) cattle based on the $U20_{SAI,\ EAI,\ banteng\ or\ gaur}$ (1%, 20%, and 100%) statistic. The dashed line represents $P < 0.005$. South Asian indicine (SAI) cattle were used as references. P values were estimated based on Z-transformed values using the standard normal distribution, and were further corrected by multiple testing using the Benjamini–Hochberg false discovery rate (FDR) method.

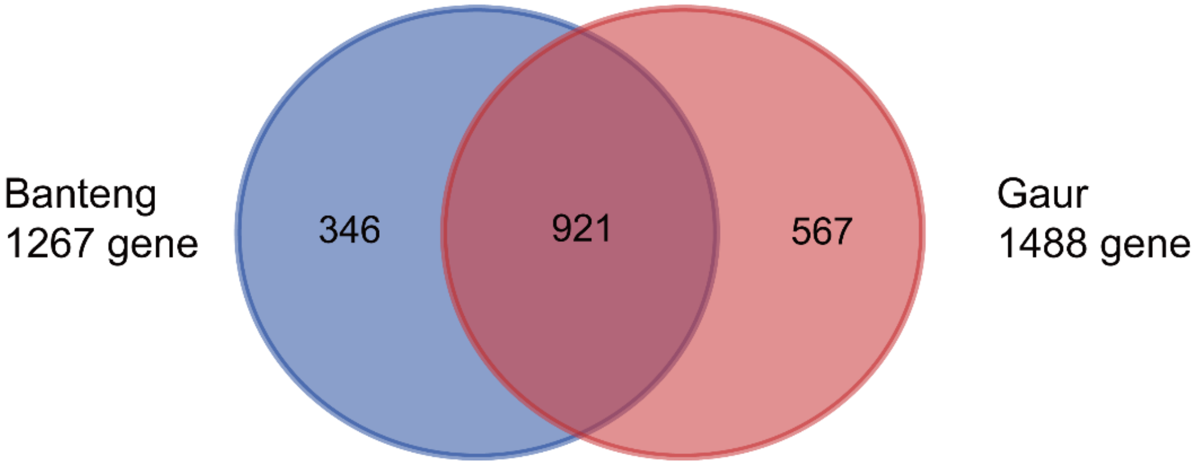

Supplementary Fig. 19 Venn diagram of the number of introgressed genes from banteng or gaur into East Asian indicine cattle. Introgressed genes were identified on the basis of the *U*20 statistic.

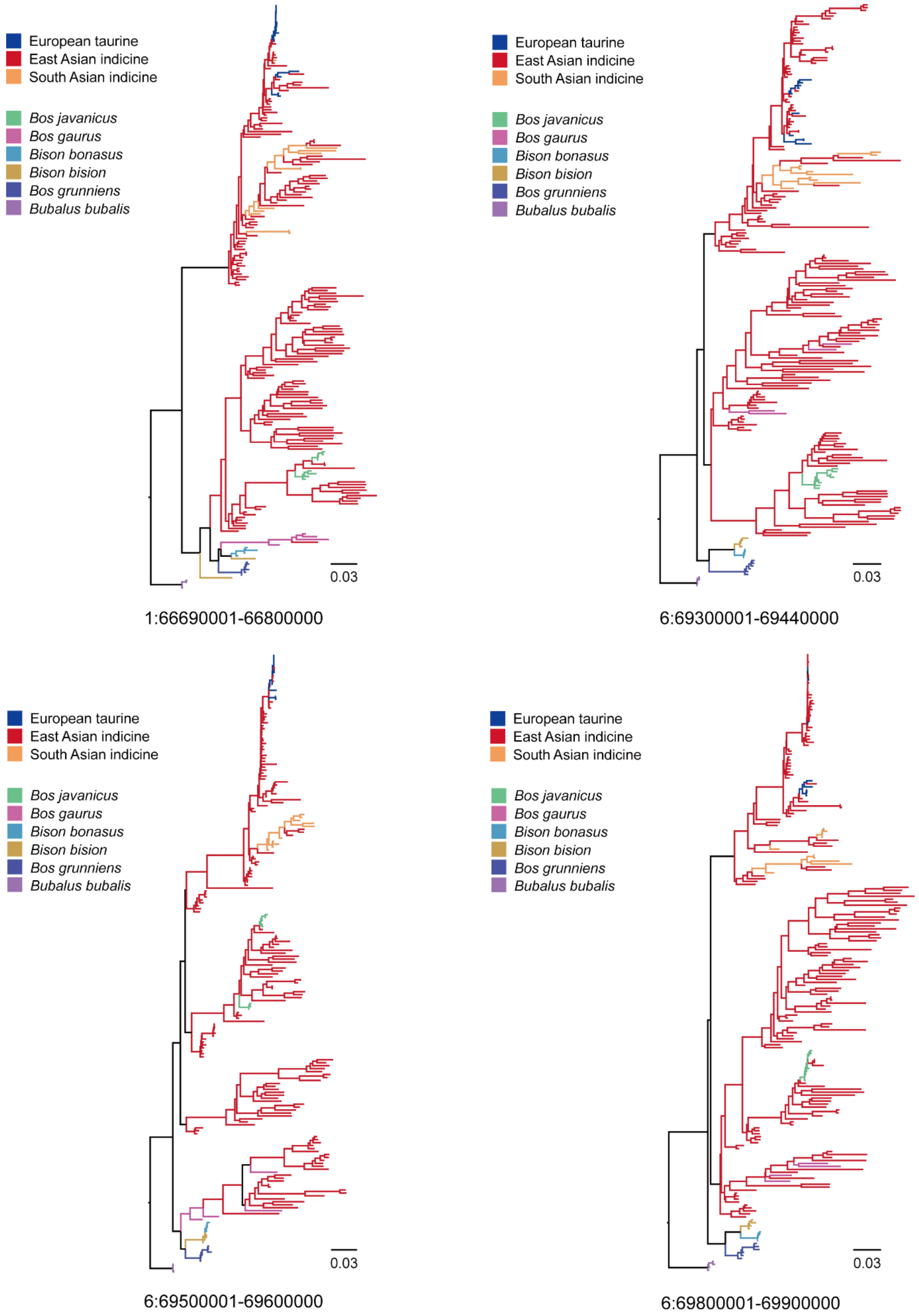

Supplementary Fig. 20 Phylogenetic trees constructed using the haplotype sequences from the BTA1:66690001-66800000, BTA6:69300001-66440000, BTA6:69500001-69600000, and BTA6:69800001-69900000 regions. Haplotypes of East Asian indicine (EAI) cattle that are clustered with banteng (*Bos javanicus*) and gaur (*Bos gaurus*) indicate banteng and gaur introgressions into EAI cattle.

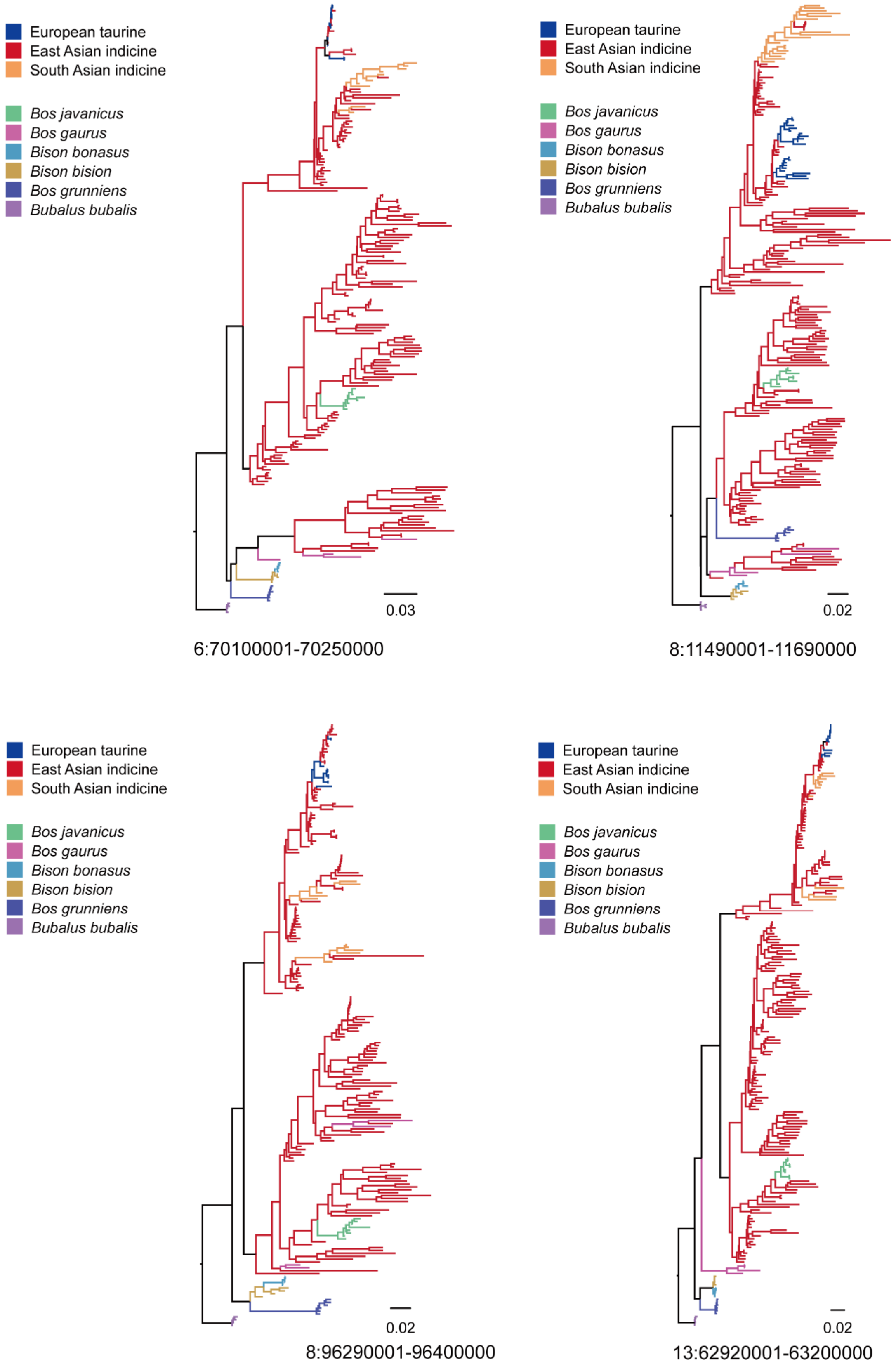

Supplementary Fig. 21 Phylogenetic trees constructed using the haplotype sequences from the BTA6:70100001-70250000, BTA8:11490001-11690000, BTA8:96290001-96400000, and BTA13:62920001-63200000 regions. Haplotypes of East Asian indicine (EAI) cattle that are clustered with banteng (*Bos javanicus*) and gaur (*Bos gaurus*) indicate banteng and gaur introgressions into EAI cattle.

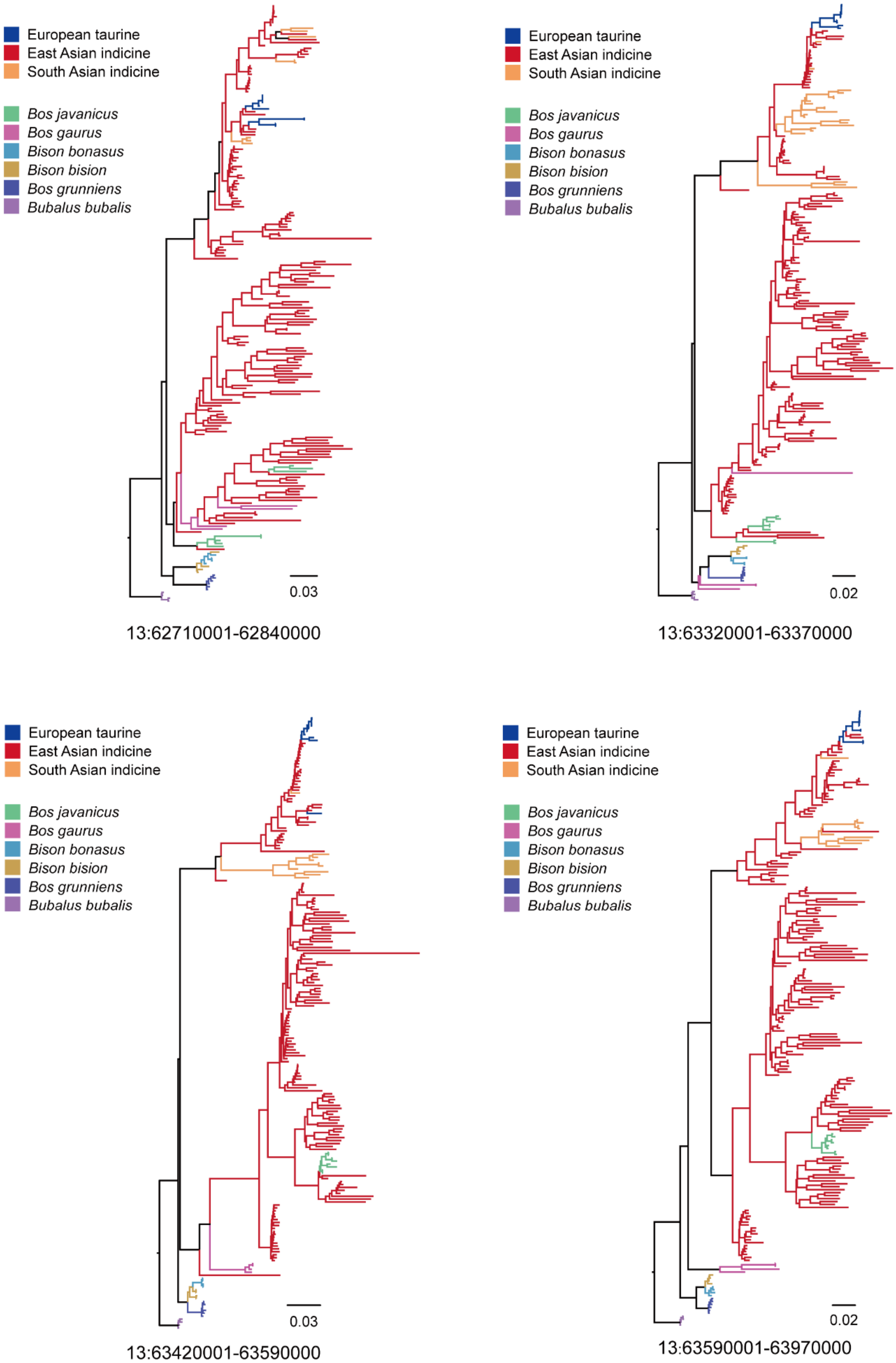

Supplementary Fig. 22 Phylogenetic trees constructed using the haplotype sequences from the BTA13:62710001-62840000, BTA13:63320001-63370000, BTA13:63420001-63590000, and BTA13:63590001-63970000 regions. Haplotypes of East Asian indicine (EAI) cattle that are clustered with banteng (*Bos javanicus*) and gaur (*Bos gaurus*) indicate banteng and gaur introgressions into EAI cattle.

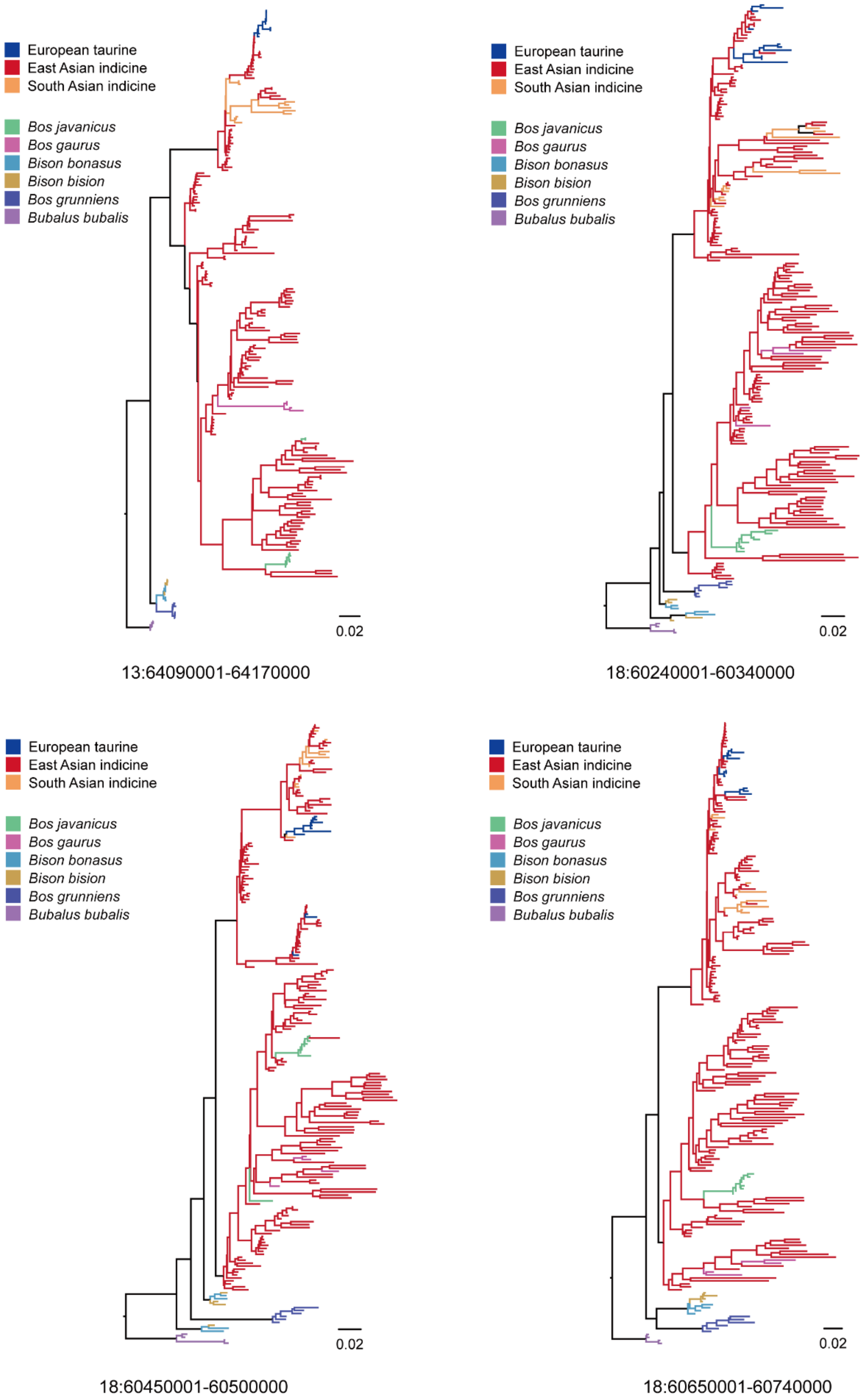

Supplementary Fig. 23 Phylogenetic trees constructed using the haplotype sequences from the BTA13:64090004-64170000, BTA18:60240001-60340000, BTA18:60450001-60500000, and BTA13:60650001-60740000 regions. Haplotypes of East Asian indicine (EAI) cattle that are clustered with banteng (*Bos javanicus*) and gaur (*Bos gaurus*) indicate banteng and gaur introgressions into EAI cattle. Dachs *et al.* (2023) [40] reports the deletion of a structural variant in BTA18:59123315-61313922, therefore all three sequences of this structural variant are removed from this figure.

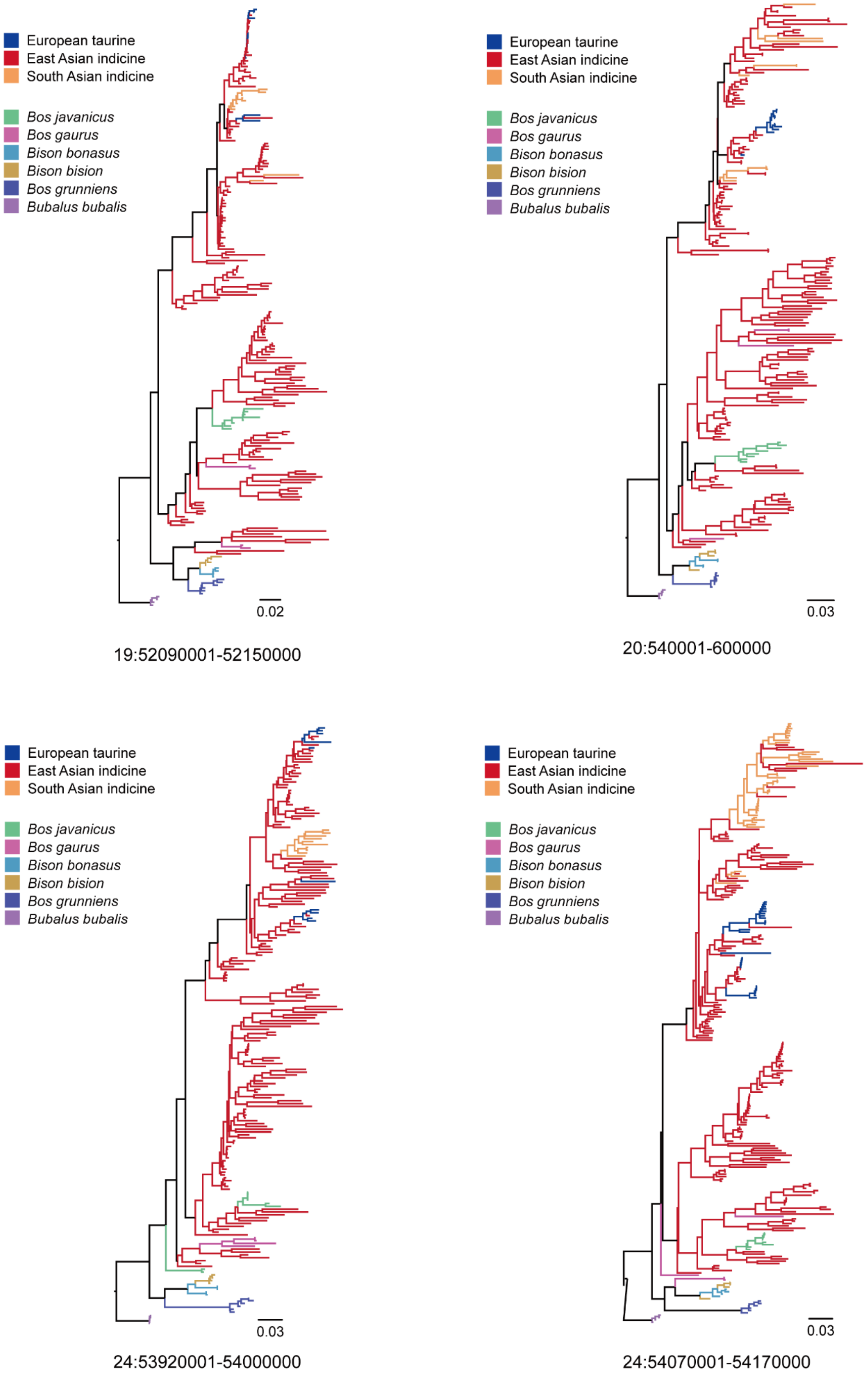

Supplementary Fig. 24 Phylogenetic trees constructed using the haplotype sequences from the BTA19:52090001-52150000, BTA20:540001-600000, BTA24:53920001-54000000, and BTA24:54070001-54170000 regions. Haplotypes of East Asian indicine (EAI) cattle that are clustered with banteng (*Bos javanicus*) and gaur (*Bos gaurus*) indicate banteng and gaur introgressions into EAI cattle.

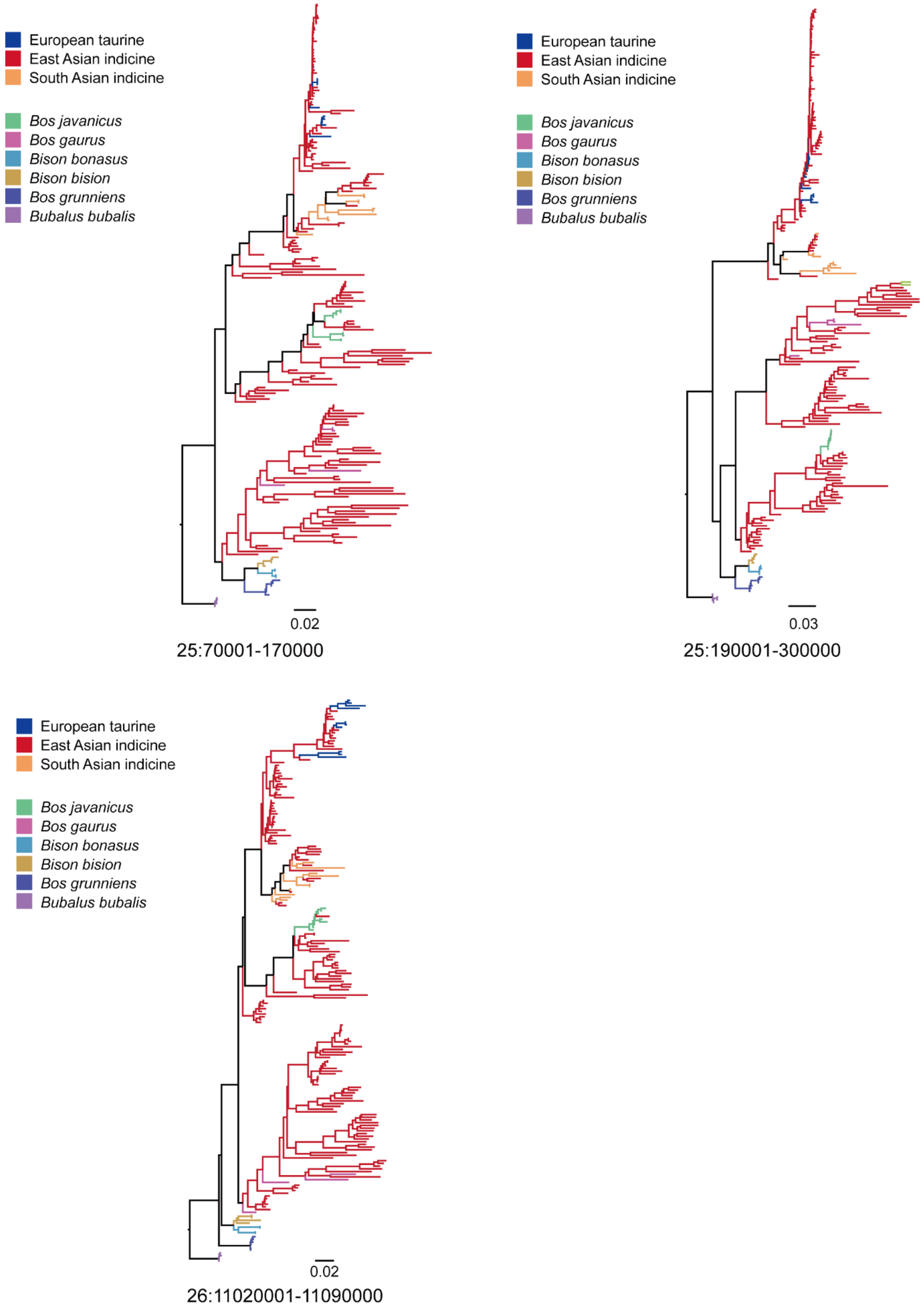

Supplementary Fig. 25 Phylogenetic trees constructed based on the haplotype sequences from the BTA25:70001-170000, BTA25:190001-300000, and BTA26:11020001-11090000 regions. Haplotypes of East Asian indicine (EAI) cattle that are clustered with banteng (*Bos javanicus*) and gaur (*Bos gaurus*) indicate banteng and gaur introgressions into EAI cattle.

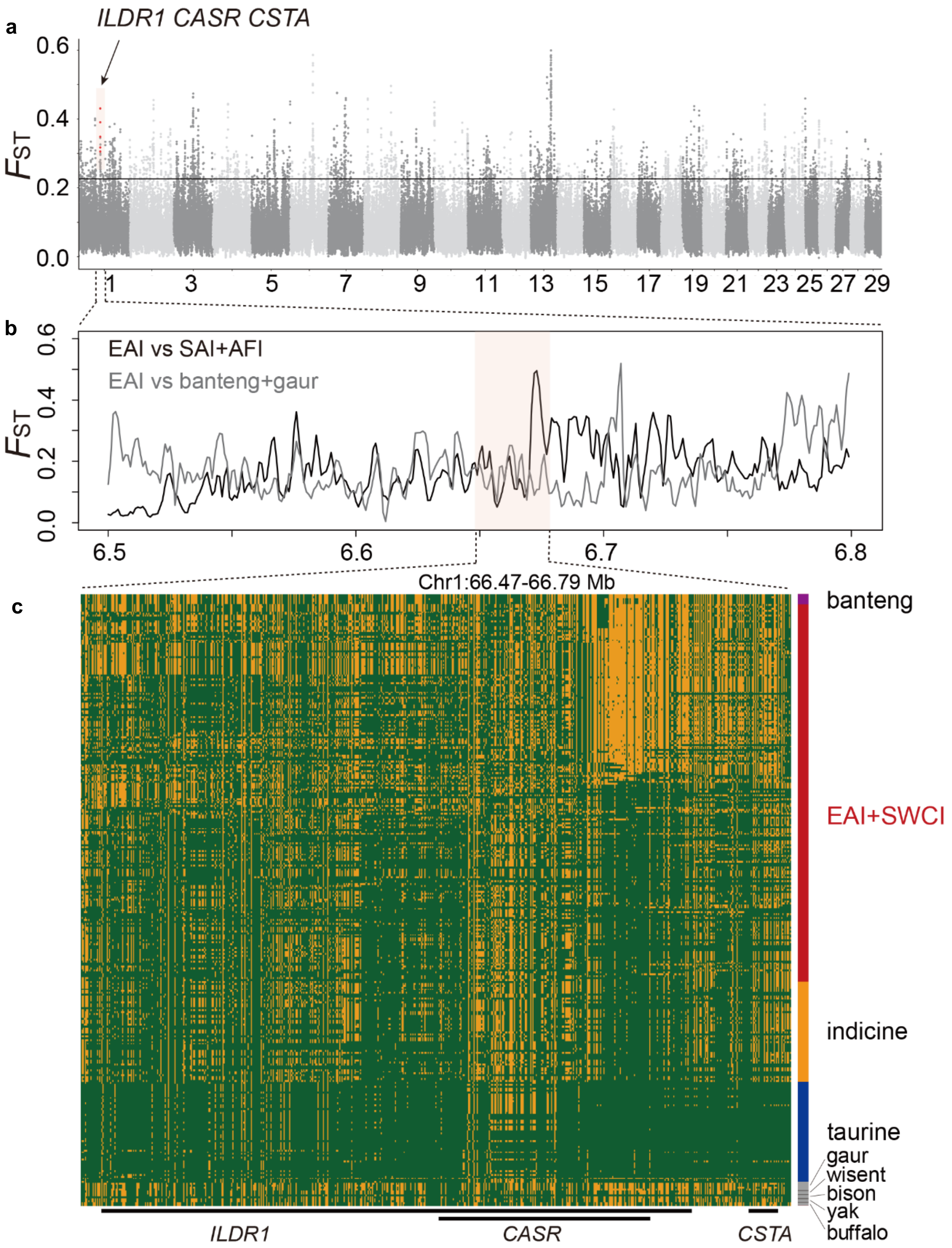

Supplementary Fig. 26 Genetic evidence of introgression of the region including the *ILDR1* gene from banteng and gaur into East Asian indicine (EAI) cattle. (a) Selective signals around the *ILDR1* gene: population branch statistic (PBS); (b) F_{ST} (EAI *vs*. South Asian (SAI) and African (AFI) indicine cattle; EAI cattle *vs*. banteng and gaur); (c) Distribution of *ILDR1* haplotypes, in which green columns represent the reference alleles and yellow columns represent the alternative alleles.

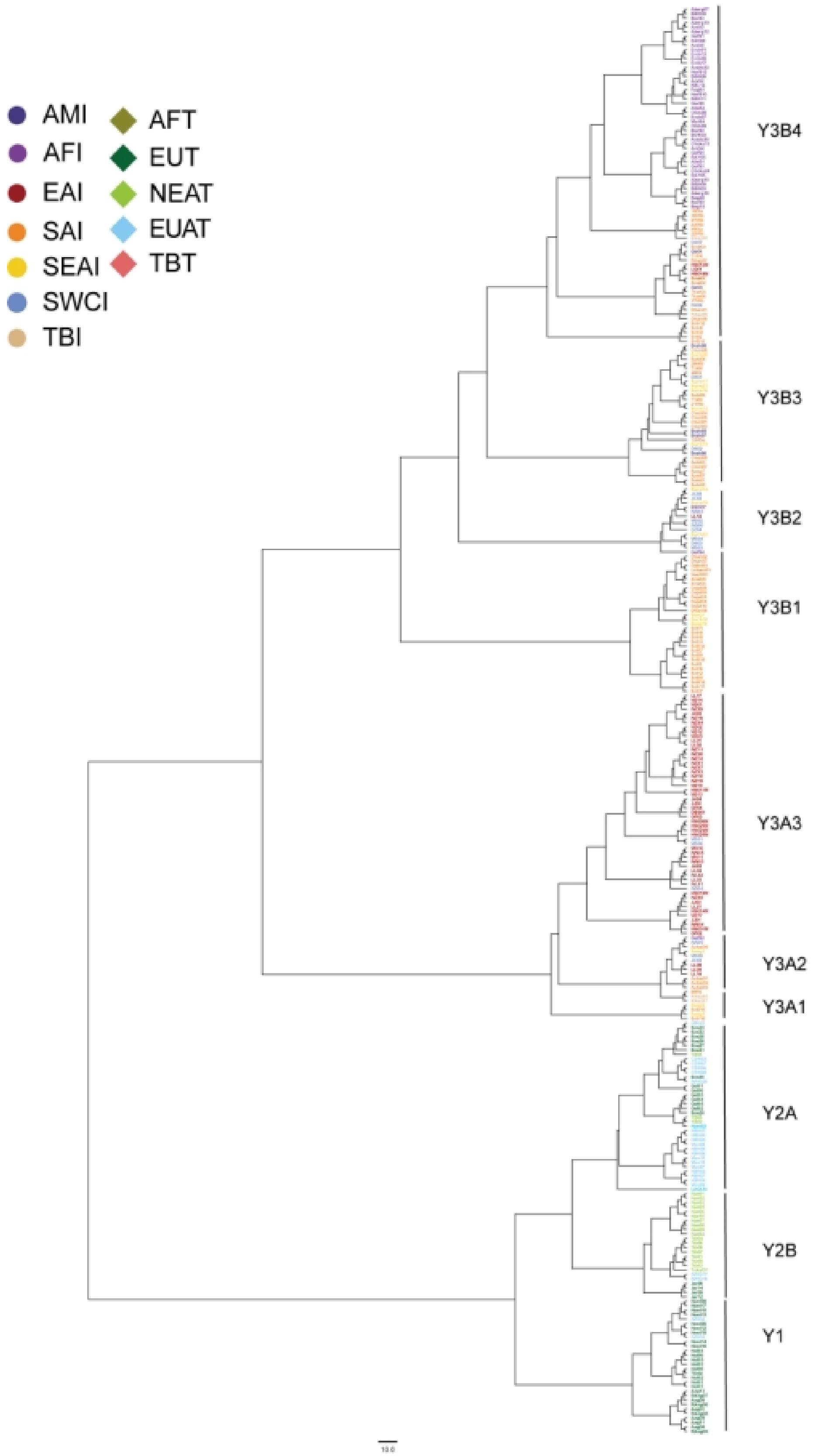

Supplementary Fig. 27 Phylogenetic tree of 309 Y chromosomal haplotypes based on 1,389 SNPs in the male-specific region of the bovine Y chromosome. Colors reflect sampling locations. (AFT, African taurine; EUT, European taurine; EUAT, Eurasian taurine; TBT, Tibetan taurine; NEAT, Northeast Asian taurine; AFI, African indicine; SAI, South Asian indicine; SEAI, Southeast Asian indicine; TBI, Tibetan indicine; SWCI, Southwest Chinese indicine; EAI, East Asian indicine; and AMI, American indicine cattle).

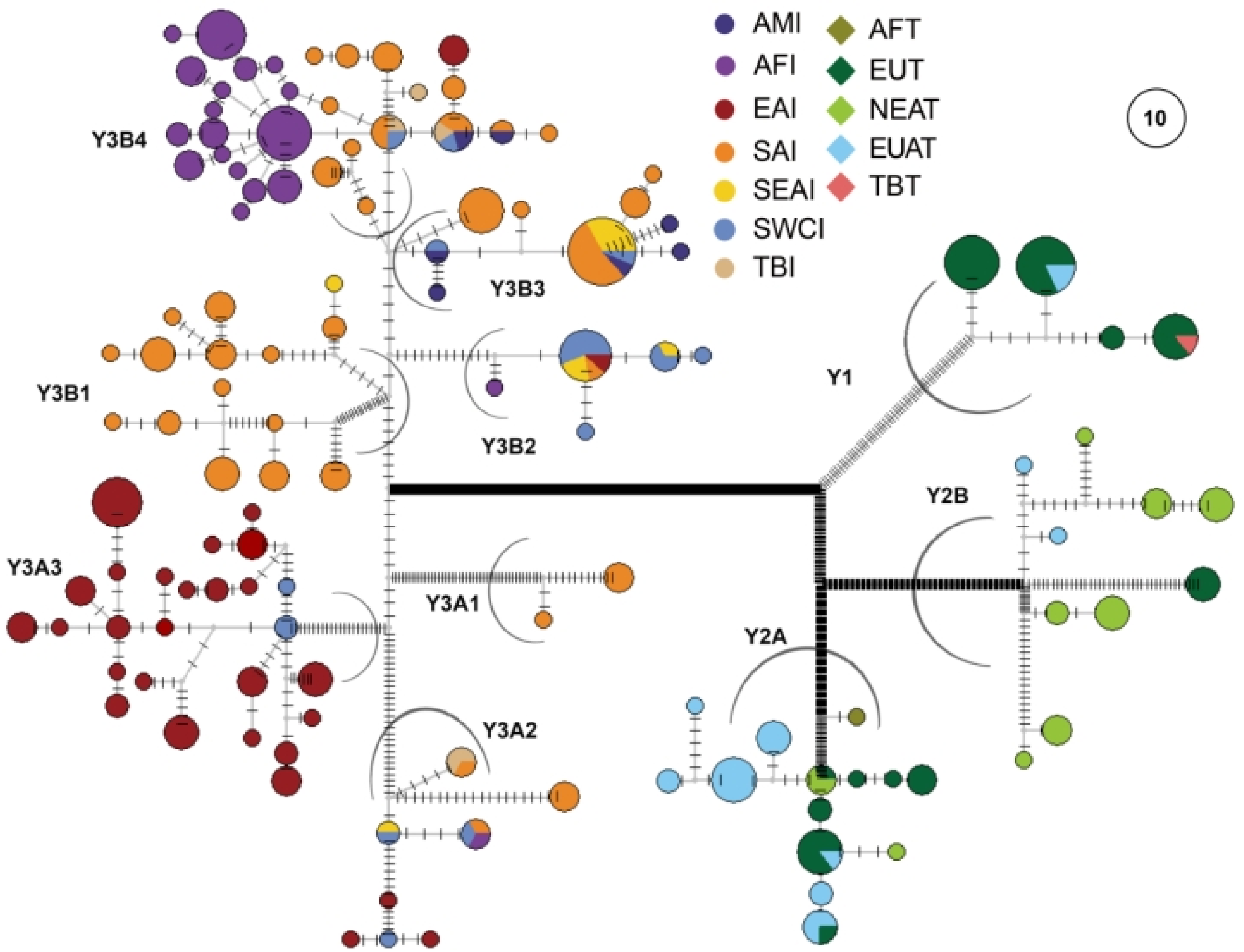

Supplementary Fig. 28 Median-joining (MJ) network of Y-chromosomal haplotypes based on 1,389 SNPs in the male-specific regions of the bovine Y chromosome. Colors reflect sampling locations. (AFT, African taurine; EUT, European taurine; EUAT, Eurasian taurine; TBT, Tibetan taurine; NEAT, Northeast Asian taurine; AFI, African indicine; SAI, South Asian indicine; SEAI, Southeast Asian indicine; TBI, Tibetan indicine; SWCI, Southwest Chinese indicine; EAI, East Asian indicine; and AMI, American indicine cattle).

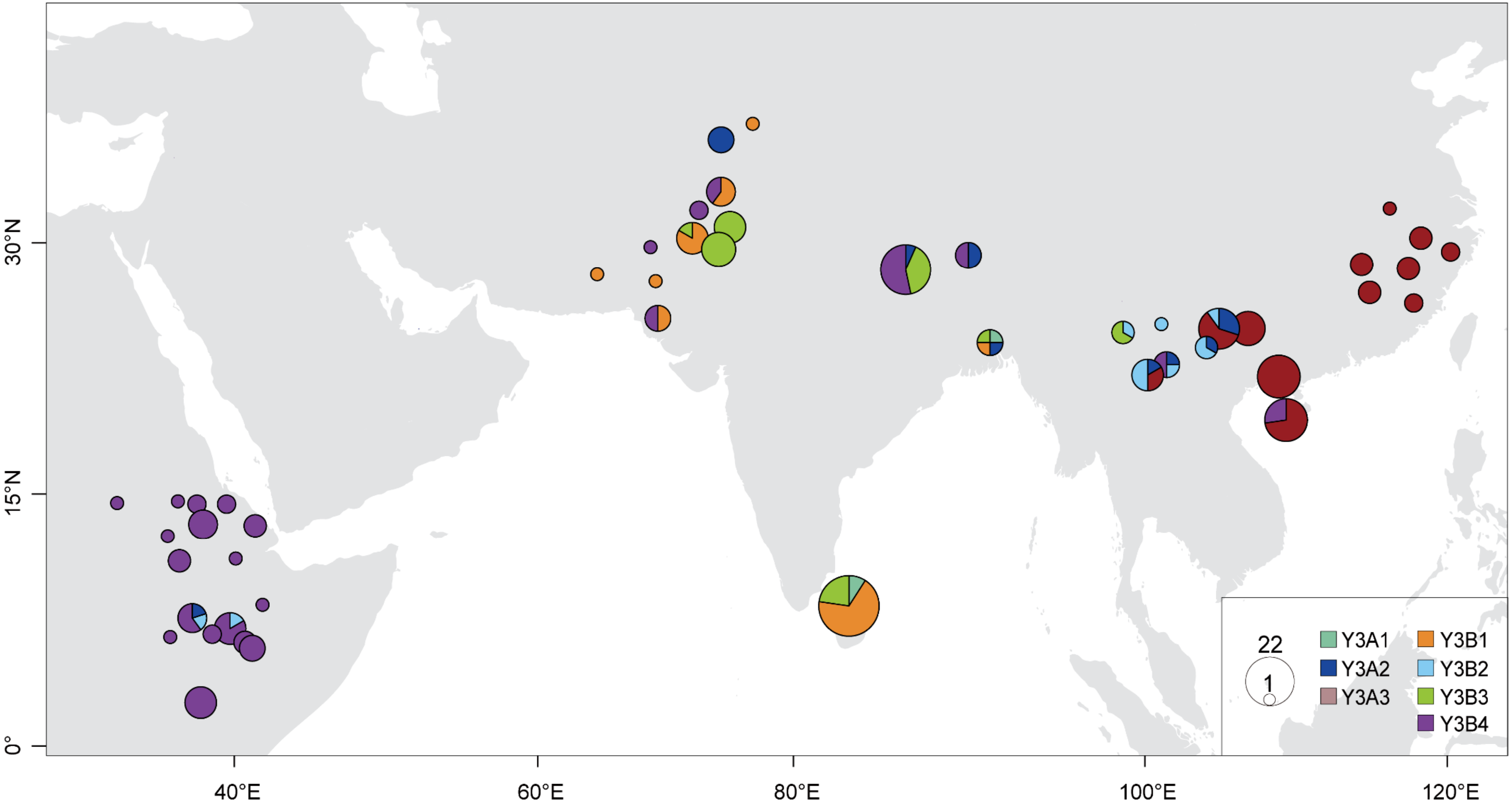

Supplementary Fig. 29 Distribution of Y chromosomal haplogroups in indicine cattle in Africa, South Asia, South China, and North-Central China. The map was drawn using the R package v4.1.0.

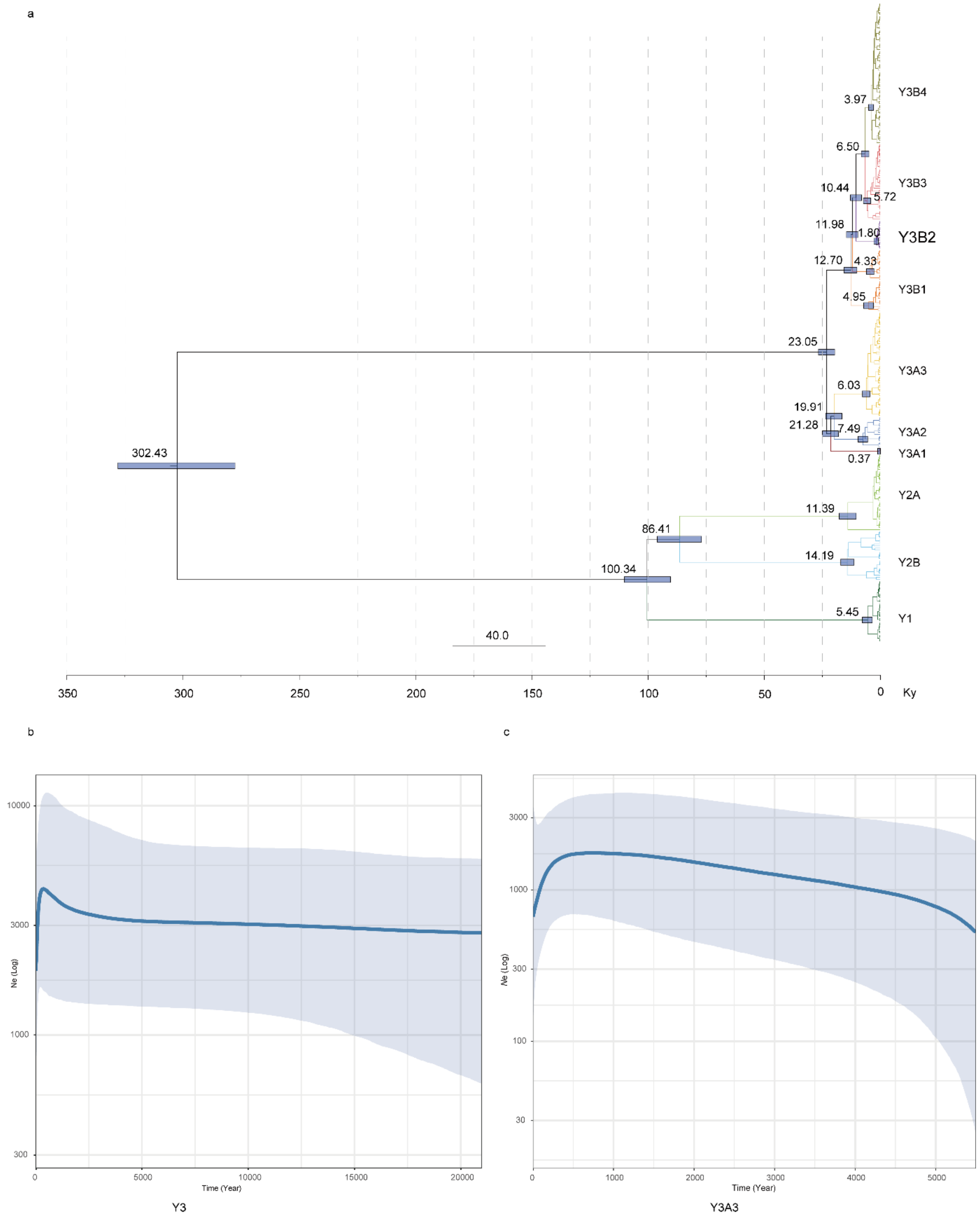

Supplementary Fig. 30 Bayesian tree inferred from 1,389 SNPs in the male-specific regions of the bovine Y chromosome and Bayesian skyline plots. (a) Bayesian tree inferred from the 1,389 SNPs. The BSPs show the trends of the effective (male) population size (*N*e on the Y axis, on a logarithmic scale) over time (X axis, in thousands of years) for the indicine Y chromosomes belonging to haplogroup Y3 (n = 218) (b) and the indicine Y chromosomes belonging to sub-haplogroup Y3A3 (n = 54) (c). The solid lines represent the median estimates of *N*e, and the shadings show the 95% highest posterior density intervals.

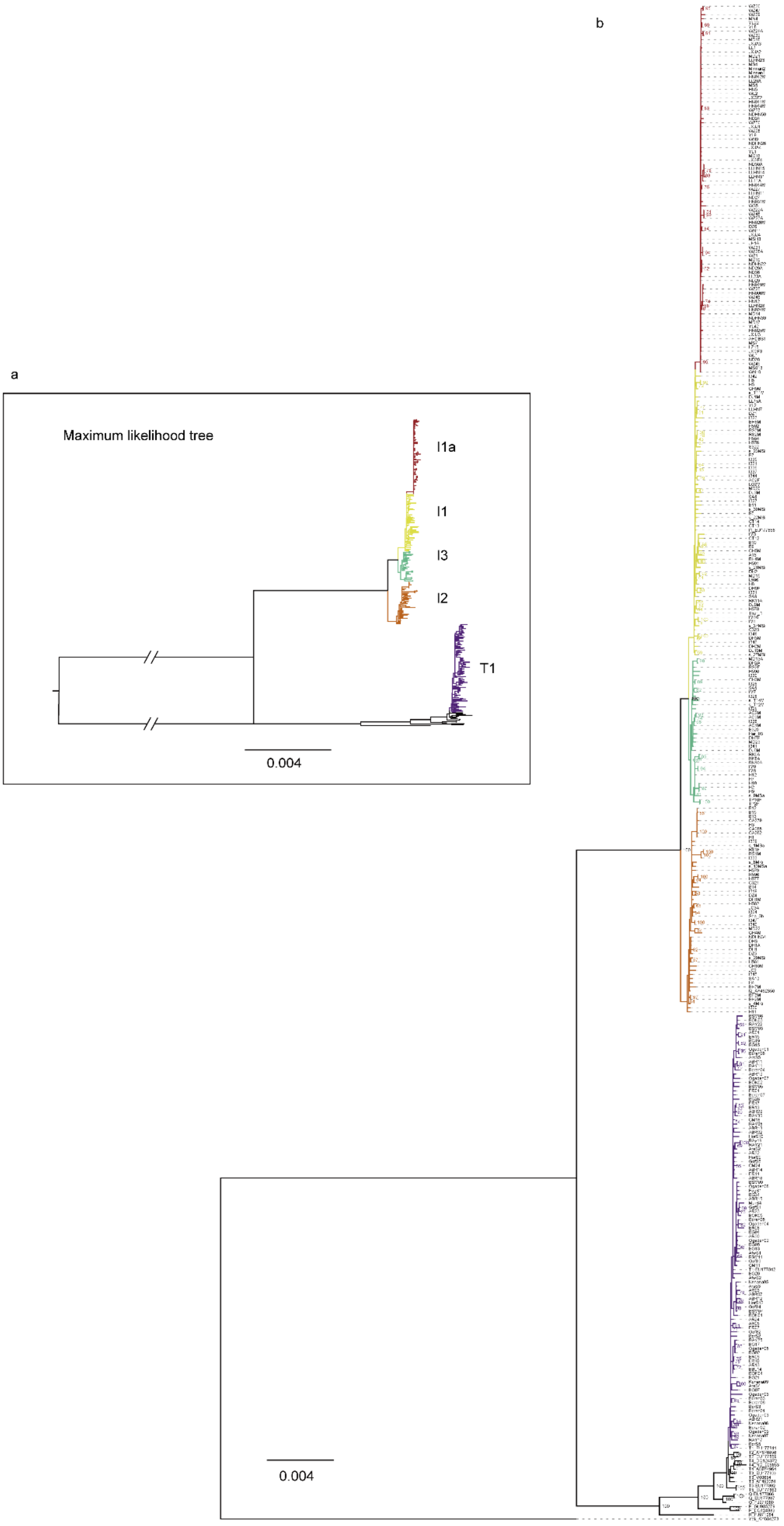

Supplementary Fig. 31 Phylogenetic tree of complete mitogenomes from indicine cattle. (a) A maximum likelihood tree of 347 mitogenomes (b) was generated using RAxML. Scale bars are based on substitutions per SNP.

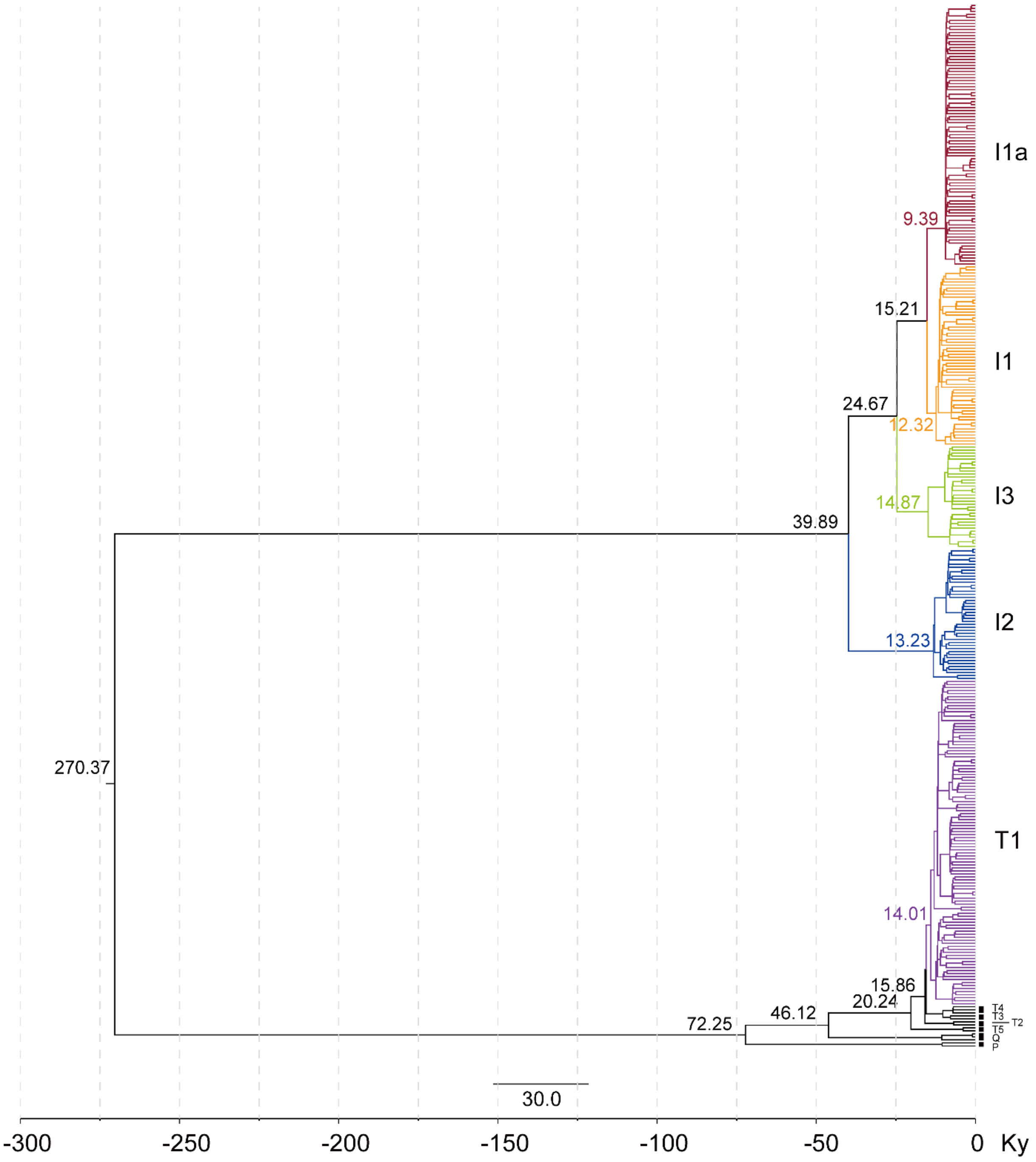

Supplementary Fig. 32 Bayesian tree inferred from complete mitogenomes of indicine cattle. X-axis, in thousands of years (Ky).

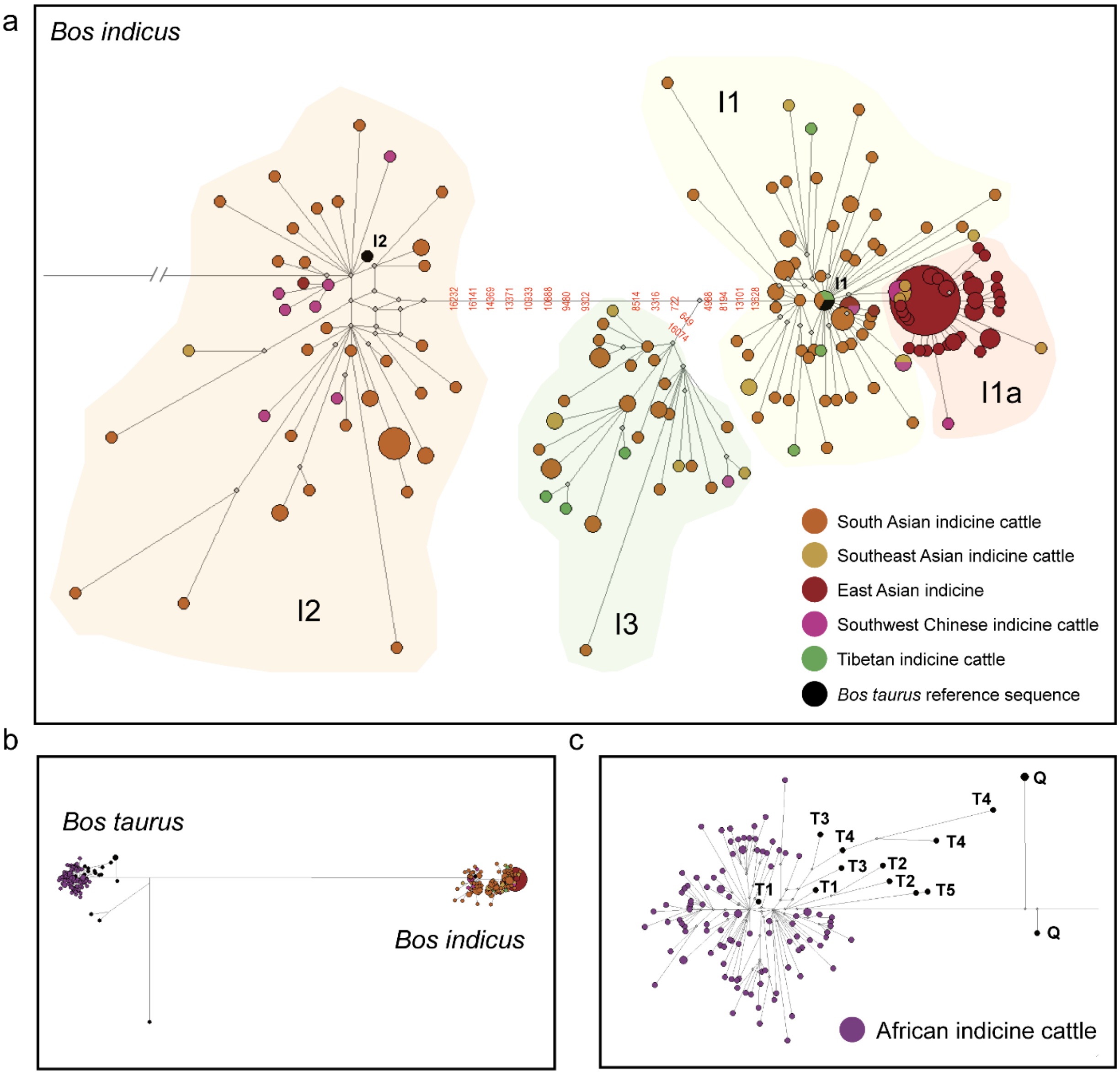

Supplementary Fig. 33 Phylogeny of complete indicine mitogenomes generated using network. (a) Median-joining (MJ) network of indicine mitogenomes. (b) MJ network of mitogenomes from taurine and indicine cattle. (c) MJ network of mitogenomes from African indicine cattle and all of them are of taurine cattle maternal origin.

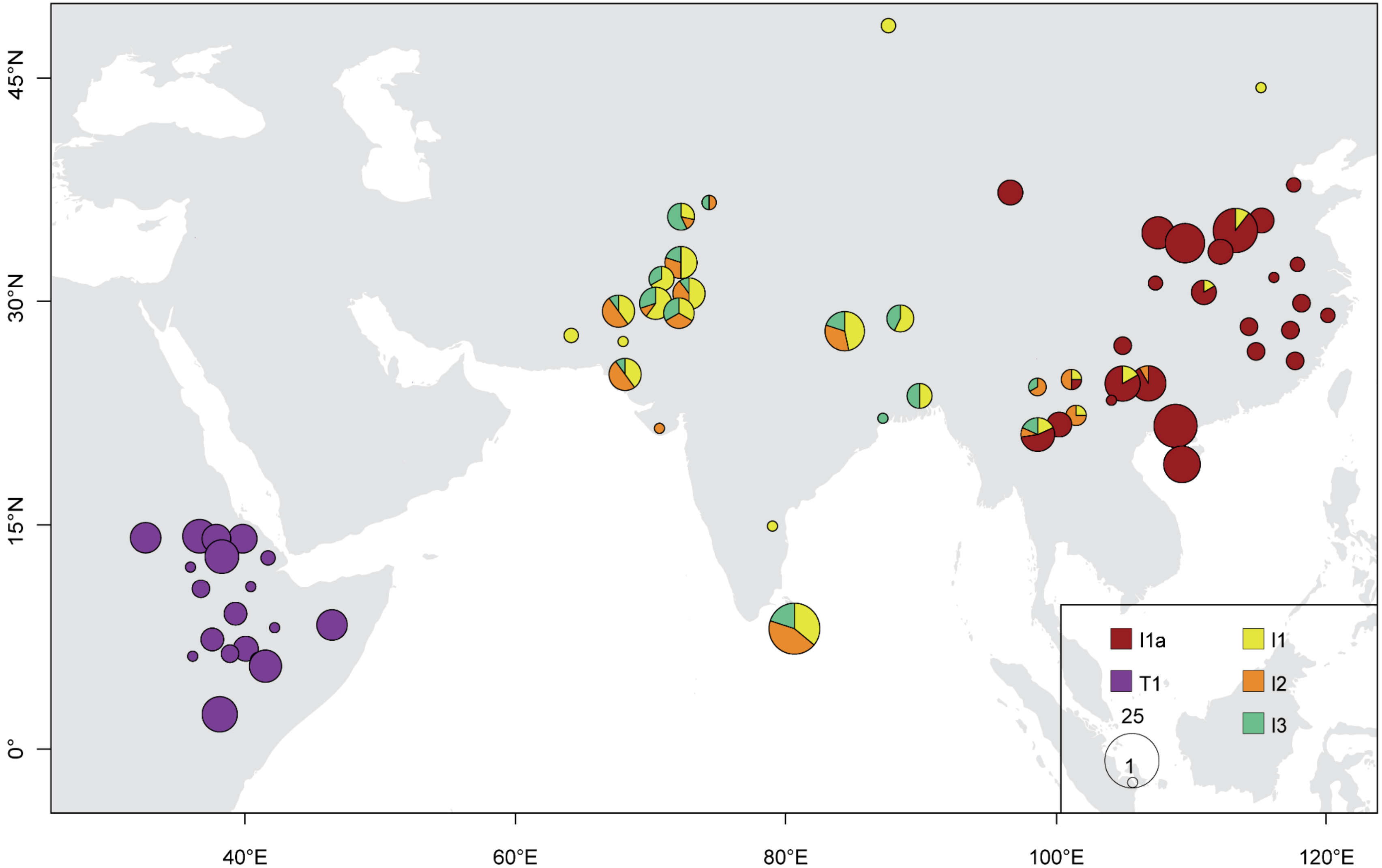

Supplementary Fig. 34 The geographic distribution of maternal haplogroups of indicine cattle in Africa, South Asia, South China, and North-Central China. The map was drawn using the R package v4.1.0.

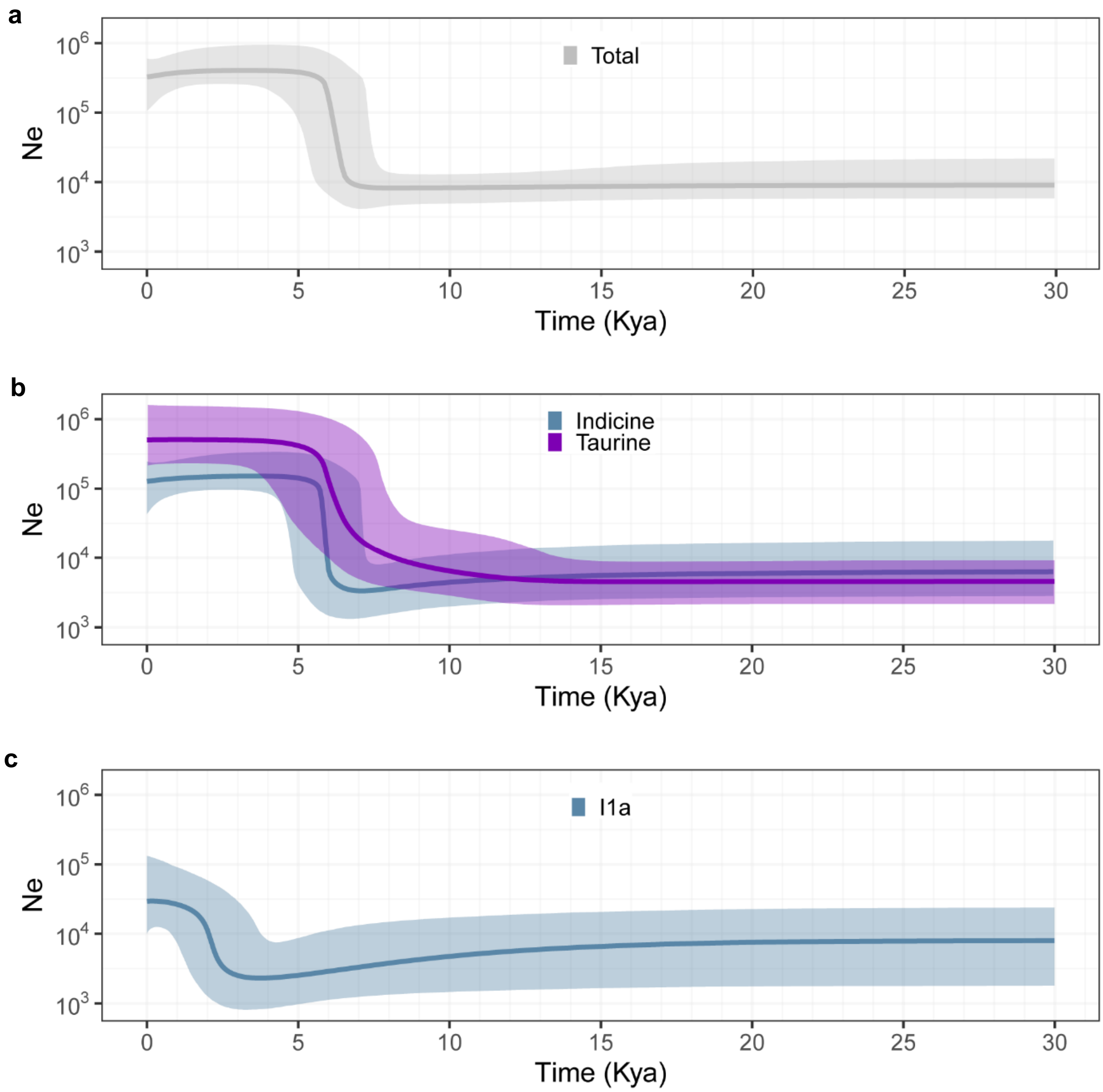

Supplementary Fig. 35 Bayesian skyline plots (BSPs) based on mitogenome coding regions. The BSPs show the trends of effective (female) population size (*N*e on the Y axis, on a logarithmic scale) over time (X axis, in thousands of years) for the total cattle samples (n = 347; in gray) (a), the indicine sequences (n = 223, in blue) (b) and the taurine samples (n = 122, in purple), and (c) the indicine mitogenomes belonging to sub-haplogroup I1a (n = 86) (c). A generation time of six years was considered. The solid lines represent the median estimates of Ne, and the shadings show the 95% highest posterior density (HPD) intervals.

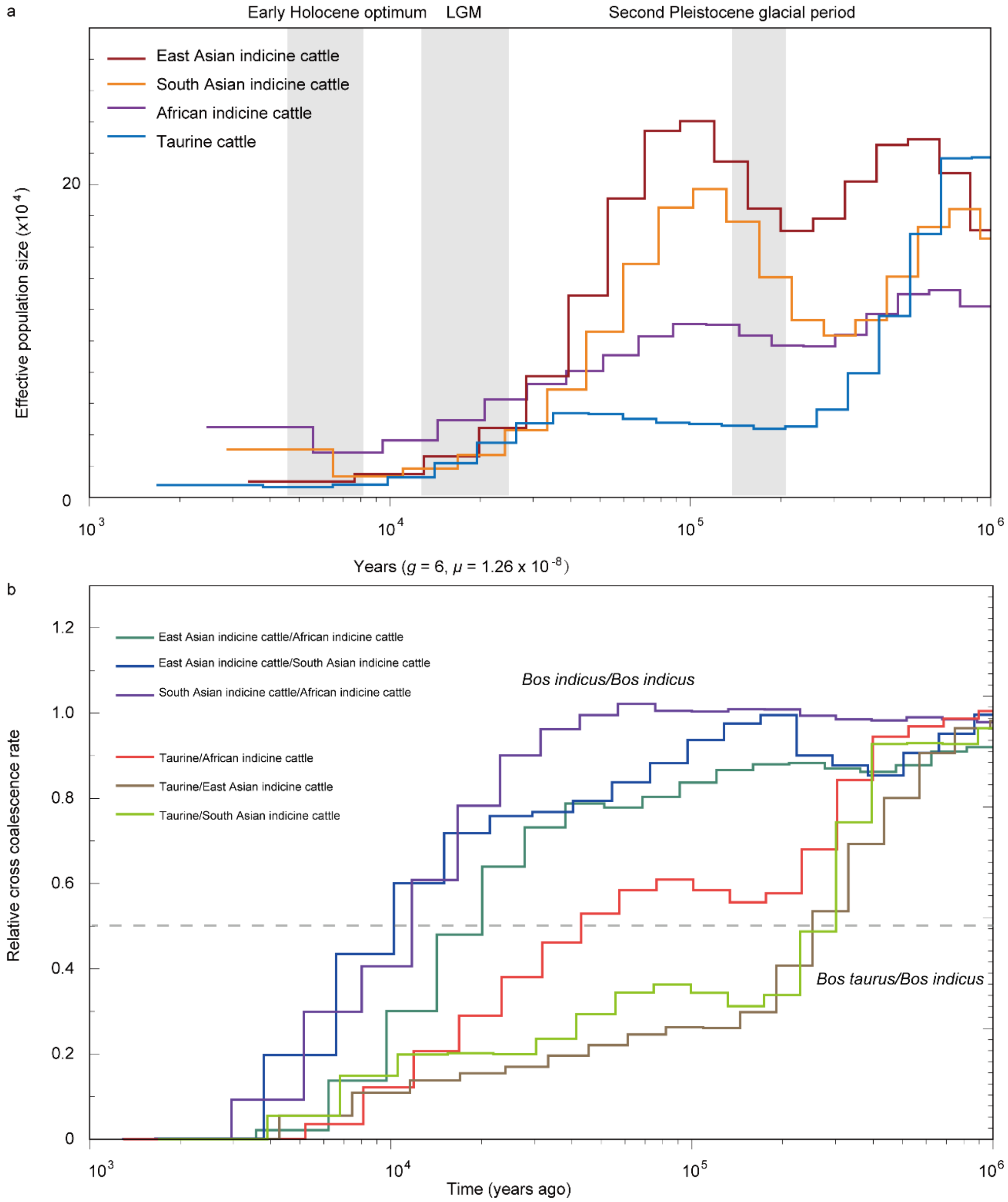

Supplementary Fig. 36 Coalescence-based inference of the demographic history of indicine cattle based on MSMC2. (a) Population size history inference of *Bos taurus* and three *Bos indicus* groups based on four haplotypes each from high-coverage individuals. The large gray-shaded boxes illustrate the Early Holocene Optimum, the Last Glacial Maximum (LGM), and the second Pleistocene Glacial Period. (b) Inferred relative cross-coalescence rates between pairs of groups over time based on four haplotypes each from South Asian indicine, East Asian indicine, African indicine, and taurine cattle. The x-axis shows time, and the y-axis shows a measure of similarity for each pair of compared groups.

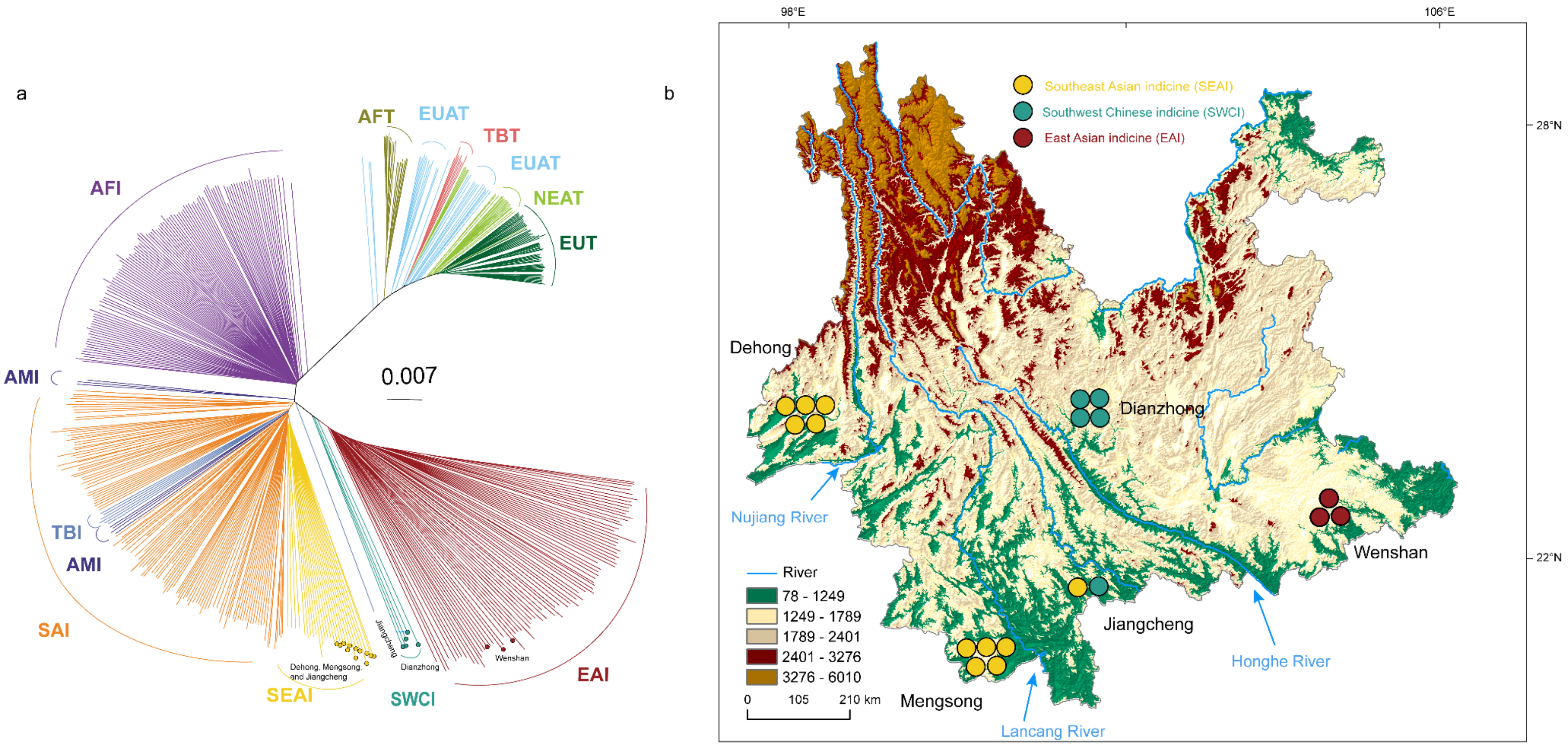

Supplementary Fig. 37 The neighbor-joining tree (a) and geographic location of indicine cattle breeds across Southwest China (b). The neighbor-joining tree was constructed using 67,162,108 autosomal SNPs. The mountainous landscape of Southwest China is traversed by three major rivers flowing from north to south (Nujiang River, Honghe River, and Lancang River). These rivers together with the mountains are likely to impede the east-to-west gene flow between Southeast Asian indicine (SEAI: 5 Dehong, 5 Mengsong, and 1 Jiangcheng cattle), Southwest Chinese indicine (SWCI: 4 Dianzhong and 1 Jiangcheng cattle), and East Asian indicine cattle (EAI: 3 Wenshan cattle). The map was drawn using the ArcGIS v10.7.0.

Supplementary Table 1 Distribution of SNPs in different genomic regions and their types.

Type of SNPs	SNP counts
Total number	67,162,108
Intergenic	37,256,470
Intronic	117,086,451
Downstream	9,814,470
Upstream	9,413,509
Exonic	3,464,029
UTR	2,105,126
Synonymous	1,913,581
Nonsynonymous	898,396
Splicing	252,870
Stop gain	8,239
Stop loss	613
Others	22,424

Supplementary Table 2 Samples and SNPs information used for different analyses.

Analysis	No. of samples	Methods	No. of SNPs	Description
Genetic diversity and population history	All 495 cattle	Neighbor-joining tree	67,162,108	A total of 67,162,108 autosomal SNPs from all 495 cattle identified with maximum missing rate < 0.1.
	All 495 cattle	Phased and imputed SNPs	65,336,403	Phased and imputed SNPs data of 65,336,403 autosomal SNPs with DR2 ≥ 0.9.
	All 495 cattle and 22 individuals of other bovine species		64,475,272	A total of 64,475,272 autosomal SNPs called from all 495 cattle and 22 individuals from other six bovine species.
	All 495 cattle and 22 individuals of other bovine species	Phased and imputed SNPs	61,532,539	Phased and imputed SNPs data of 61,532,539 autosomal SNPs using Beagle with default parameters and filtering with DR2 < 0.9.
	All 495 cattle	PCA and Admixture	2,996,368	LD-pruned SNPs of the 67,162,108 autosomal SNPs with the “MAF 0.01” and “-indep-pairwise 50 10 0.1” options.
	354 indicine cattle	PCA	2,565,770	LD-pruned SNPs of 36,838,260 autosomal SNPs
	331 cattle	Runs of homozygosity	65,336,403	We filtered samples with a mapping depth < 10 × or 3× genome coverage < 90% and used 331 individuals for ROH analysis. We used phased and imputed SNPs to detect ROH using PLINK. The final parameters were set to a minimum length of 100 kb, a scanning window size of 100 SNPs, a minimum density threshold of 200 SNPs, a large gap of 1000 kb, a maximum number of heterozygous SNPs in the scanning window of 1 and a scanning window threshold level of 0.05.
	484 cattle	Mean pairwise F_{ST} values between cattle breeds/populations	65,160,804	Mean pairwise F_{ST} values between cattle breeds/populations represented by more than one animal.
	All 495 cattle and 3 yak	Population level maximum likelihood phylogeny	15,228,801	A total of 15,228,801 SNPs were retained for ML phylogenetic analysis in TreeMix using the “--maf 0.001 --geno 0.1” and “-indep-pairwise 50 10 0.1” parameters in PLINK. The parameters for TreeMix were “treemix -i -root yak”.
MSMC2	Eight cattle	Phased and imputed SNPs	34,458,512	Phased and imputed SNPs data of 34,915,905 SNPs with DR2 > 0.9.
Selective sweeps	All 495 cattle	F_{ST} and θ_π	67,162,108	F_{ST} values were calculated in 50 kb windows with 20 kb steps using VCFtools, with the parameters “--max-missing 0.9 --maf 0.05”. θ_π was calculated using VCFtools. Top 1% windows were identified as significant genomic regions.
	All 495 cattle	XPEHH	65,336,403	XP-EHH statistics based on the extended haplotype were calculated for each population pair using selscan v1.1. For the XP-EHH selection scan, our test statistic was the average normalized XP-EHH score in each 50 kb window. We performed sliding-window analyses to calculate average XP-EHH values with a window size of 50 kb and a step size of 20 kb.
	All 495 cattle	*iHS*	65,336,403	We calculated the standardized *iHS* across the genome using selscan v1.1 with default settings. norm software was used to normalize the *iHS* scores within 50 kb windows. We performed sliding-window analyses to calculate *iHS* values with a window size of 50 kb and a step size of 20 kb.
	All 495 cattle	CLR	65,336,403	We performed the CLR test in 50 kb windows with 20 kb steps using SweepFinder2 with the default parameter settings.
Introgression analysis	All 495 cattle and 22 individuals of six other bovine species	TreeMix	10,558,724	A total of 10,558,724 SNPs were retained for TreeMix using the "--maf 0.001 --geno 0.1" parameters in PLINK.
		D statistic	64,475,272	We calculated the *D* statistics among the selected group (4 banteng, 2 gaur, 15 taurine cattle, and 15 SAI cattle) using ADMIXTOOLS with qpDstat module and Dsuite with the Dtrios module (Dsuite Dtrios). Standard error was obtained by a block JackKnife approach and *Z* scores was statistical significance at $\|Z\| > 3$ typically.
		f_3	64,475,272	To detect gene flow among the selected groups (4 banteng, 2 gaur, 15 taurine cattle, and 15 SAI cattle), we also used the three-population test (f_3 statistics) and calculated their corresponding normalized value (*Z* scores) at the group level in the “qp3Pop” program implemented in ADMIXTOOLS v.6.0.
		Introgression RFMix	61,532,539	Local ancestry was inferred using RFMix based on the phased data with the parameters recommended in the documentation and setting four groups of populations as references for four different ancestries: taurine ancestry, indicine ancestry, banteng ancestry and gaur ancestry.
		Introgression *U*20 and *U*50	61,532,539	50 kb windows and 20 kb steps were used to calculate the statistic $U_{A,B,C}$. Banteng/gaur was set as the reference populations.

Supplementary Table 3 ADMIXTURE cross-validation errors from $K = 2$ to $K = 8$.

***K* value**	**CV error**
$K = 2$	0.28461
$K = 3$	0.27155
$K = 4$	0.26690
$K = 5$	0.26442
$K = 6$	0.26332
$K = 7$	0.26495
$K = 8$	0.26402

Supplementary Table 4 Common candidate genomic regions identified in indicine cattle based on the F_{ST}, θ_π, and XP-EHH analyses.

BTA	Regions (Mb)	F_{ST}	θ_π	XP-EHH	Genes identified	Association	References
1	44.08-44.19	0.70	0.31	4.18	*CMSS1, FILIP1L*	Adiposity, neoplastic	
1	81.58-81.69	0.74	0.23	3.42	*SENP2, LIPH*	Adiposity, hair development	[41]
5	24.84-24.89	0.63	0.17	0.72	*FGD6*	Growth and feed efficiency	
5	112.38-112.45	0.79	0.33	3.07	*L3MBTL2, CHADL, RANGAP1, ZC3H7B*		
7	43.04-43.09	0.63	0.16	1.18	*MIER2, THEG*		
7	43.16-43.21	0.72	0.24	2.62	*MADCAM1, TPGS1*		
7	43.18-43.29	0.69	0.49	2.62	*CDC34, FGF22, GZMM, BSG, HCN2, POLRMT, RNF126, FSTL3, PRSS57*	Hair development	[41]
7	44.58-44.67	0.68	0.50	3.44	*ZCCHC10, HSPA4*	DNA damage repair, heat stress	
7	50.14-50.31	0.83	0.57	0.78	*LRRTM2, CTNNA1, SIL1, MZB1, PROB1, PAIP2, SLC23A1*	Brain development, muscle development, antiviral immunity, reproduction, vitamin C transporters	[12]
7	50.64-51.15	0.84	0.58	2.26	*SPATA24, DNAJC18, TMEM173, UBE2D2, ECSCR, CXXC5, PSD2, NRG2*	Fertility and reproduction, heat stress	[12]
7	51.40-51.47	0.77	0.35	1.67	*PFDN1, CYSTM1*		
7	51.54-51.61	0.68	0.30	1.24	*HBEGF, SLC4A9*		
7	52.10-52.19	0.71	0.85	1.69	*PCDHB1, PCDHA13*		
7	52.92-52.97	0.63	0.44	1.65	*KIAA0141, PCDH1*		
8	0.20-0.37	0.69	0.56	3.02	*MFSD14B*		
8	39.38-39.43	0.63	0.21	1.95	*JAK2*		
8	53.22-53.27	0.64	0.32	2.64	*VPS13A*	Blood circulation	[42]
8	59.36-59.41	0.65	0.45	2.64	*FAM214B, STOML2, UNC13B*		
8	69.62-69.73	0.69	0.31	3.39	*PIWIL2, SLC39A14*	Mn^{2+} and Fe^{2+} homeostasis	
10	37.10-37.17	0.64	0.27	2.89	*MGA*		
16	50.50-50.67	0.74	0.22	3.02	*MORN1, PRKCZ, FAAP20*	Light response, DNA damage	[43, 44]
18	39.52-39.61	0.75	0.30	3.33	*CHST4*		
19	26.38-26.45	0.72	0.34	3.62	*SPAG7, PFN1, KIF1C, CAMTA2, ENO3*	Antiviral immunity, skeletal development, neurodegenerative disease, cardiac growth, muscle development and glycogen storge,	[45, 46,47,48, 49]
19	27.40-27.61	0.74	0.94	2.96	*EFNB3, DNAH2, WRAP53, TMEM88, NAA38, CYB5D1, CHD3, RNF227, KCNAB3, KDM6B*	DNA damage, heart development	[50, 51]
19	27.82-27.91	0.74	0.57	1.41	*TMEM107, BORCS6, RANGRF, SLC25A35, AURKB, CTC1, PFAS*		
19	42.56-42.63	0.74	0.31	1.28	*NAGLU, HSD17B1, ATP6V0A1*		
19	44.52-44.57	0.66	0.2	1.14	*GJC1, EFTUD2, HIGD1B*		
20	71.46-71.53	0.66	0.47	1.51	*CEP72*	Feed efficiency	
22	55.80-55.85	0.64	0.15	2.20	*TAMM41*	Heart valve development	[52]
28	44.30-44.35	0.63	0.17	2.86	*ALOX5, MARCH8*		
29	49.50-49.63	0.72	0.21	3.71	*MRPL23, PRR33, TNNT3, LSP1*	Inflammation, muscle and skeletal development	[53, 54]

Supplementary Table 5 Results from the enrichment analysis of genes under selection in South Asian indicine cattle. The GO and KEGG analyses were performed with KOBAS based on the lists of genes present in the genomic regions under selection. The *P* value was calculated using a hypergeometric distribution. False discovery rate (FDR) correction was performed to adjust for multiple testing. Pathways with an FDR-corrected *P* value of < 0.05 were considered statistically significantly enriched.

**Terms**	**ID**	**Input genes**	**No. genes**	**Corrected *P* values**	**Genes**
Glycosaminoglycan biosynthesis - keratan sulfate	bta00533	3	14	0.023099	*ST3GAL3|B4GALT2|CHST4*
Human papillomavirus infection	bta05165	10	345	0.024619	*ATP6V0B|ITGA9|EP300|TP53|GNAS|PARD6G|ITGA2B|PIK3CD|LAMB2|HES7*
Axon guidance	bta04360	7	180	0.034706	*EFNB3|SEMA3F|PARD6G|PIK3CD|SEMA4B|GNAI2|SEMA6C*
Positive regulation of transcription elongation from RNA polymerase II promoter	GO:0032968	3	10	0.021279	*CDK12|PAF1|RTF1*
Fat cell differentiation	GO:0045444	5	65	0.023099	*ALOXE3|EP300|MED1|SENP2|BSCL2*
Heterotrimeric G-protein complex	GO:0005834	4	29	0.021279	*GNAT1|GNAI2|GNG3|GNAS*
Semaphorin receptor binding	GO:0030215	3	14	0.023099	*SEMA4B|SEMA6C|SEMA3F*
Arachidonic acid metabolic process	GO:0019369	3	15	0.024324	*ALOXE3|ALOX15|ALOX12E*
mRNA 3'-UTR binding	GO:0003730	4	51	0.044347	*ZFP36|TP53|FXR2|RNF40*

Supplementary Table 6 Common candidate genomic regions identified in East Asian indicine cattle based on the CLR, F_{ST}, θ_π, and PBS analyses.

BTA	Regions (Mb)	Windows	CLR	F_{ST}	θ_π	PBS	Genes identified
1	82.80-82.91	4	125.95	-	8.44	-	*POLR2H|FAM131A|CHRD|THPO|EIF4G1|PSMD2|ECE2|CLCN2*
1	105.30-105.37	2	88.42	0.24	-	0.49	
1	136.86-136.97	4	191.78	-	8.00	-	*UBA5|NPHP3|ACAD11*
2	125.72-125.89	7	236.79	-	8.17	-	*AHDC1|WASF2*
2	125.98-126.15	6	103.67	-	7.50	-	*WDTC1|SLC9A1*
2	126.80-126.91	4	250.86	-	7.94	-	*CD52|SH3BGRL3|CRYBG2|UBXN11|CEP85|CATSPER4*
3	50.20-50.27	2	69.18	-	7.70	0.33	*CCDC18*
7	17.46-17.53	2	89.03	0.26	-	-	*LOC100337081|LOC518134*
7	43.20-43.31	4	303.34	-	8.31	-	*FGF22|FSTL3|PRSS57|BSG|HCN2|POLRMT|RNF126|PALM*
7	43.32-43.39	2	128.77	-	8.19	-	*MISP|PALM|PTBP1|PLPPR3*
7	44.52-44.65	5	232.28	-	7.94	-	*ZCCHC10|AFF4|HSPA4*
7	50.02-50.29	11	414.28	-	8.18	-	*LRRTM2|CTNNA1|SIL1*
7	50.60-50.99	18	442.38	-	8.67	-	*MZB1|PROB1|SMIM33|PAIP2|SLC23A1|SPATA24|DNAJC18|TMEM173|UBE2D2|MATR3|ECSCR|CXXC5|PSD2*
7	51.10-51.19	3	69.86	0.39	-	-	*NRG2*
7	52.64-52.81	7	270.57	-	9.15	0.45	*RELL2|HDAC3|FCHSD1|ARAP3|DIAPH1*
8	52.02-52.11	3	254.54	-	7.73	-	*PCSK5*
9	41.80-41.89	3	89.10	-	7.80	0.52	*SNX3|AFG1L*
11	61.24-61.43	8	844.43	-	8.34	-	*EHBP1*
11	61.50-61.77	12	1152.67	-	7.89	-	*OTX1|EHBP1|WDPCP*
11	73.40-73.51	4	157.79	-	7.66	0.46	*KIF3C|RAB10*
13	22.68-22.93	11	571.10	-	9.67	-	*CASC10|SKIDA1|MLLT10*
14	35.56-35.67	4	91.75	0.24	-	0.60	*TRPA1*
16	7.98-8.11	5	706.58	0.43	8.70	1.61	
16	8.16-8.67	24	1799.85	0.31	9.29	1.96	
16	8.68-8.79	4	394.45	0.24	8.04	1.33	
16	8.80-8.93	5	537.97	0.27	-	1.42	*LOC789494*
19	26.88-26.99	4	227.58	-	8.59	-	*ASGR1|DVL2|GABARAP|CTDNEP1|CLDN7|DLG4|ACADVL|PHF23|ELP5|SLC2A4*
19	28.14-28.25	4	167.38	-	7.98	0.52	*MYH10*
22	50.06-50.39	14	285.31	0.44	9.14	0.87	*GNAT1|IFRD2|SEMA3B|GNAI2|SLC38A3|SEMA3F|RBM5|MON1A|MST1R|LOC616410|CAMKV|LSMEM2|RBM6*
26	38.56-38.65	3	233.16	0.33	-	0.42	*FAM204A*
29	10.02-10.09	2	73.68	-	7.70	-	*DLG2*
29	10.38-10.53	6	236.41	-	8.19	-	*DLG2*

Supplementary Table 7 Results of f_3 statistics performed to detect admixtures among 4 banteng, 2 gaur, 15 South Asian indicine (SAI), and 15 taurine cattle. The f_3 statistic considers the population triplet (A, B, and C), where C is the test (target) population and A and B are the reference (source) populations. If the *Z* score ($Z \leq -3.0$) is significantly negative, the test population has admixture from both reference populations A and B. Indicine group in SAI cattle include three individuals from three breeds (Thawalam, Dajal, and Sahiwal).

**PopA**	**PopB**	**Pop C**	f_3	***Z* scores**	**PopA**	**PopB**	**Pop C**	f_3	***Z* scores**
**Taurine**	**Banteng**	**Taurine**			**Taurine**	**Gaur**	**Taurine**		
Angus	Banteng	Finncattle	0.090	24.405	Angus	Gaur	Finncattle	0.091	24.453
Angus	Banteng	Holstein	0.051	19.002	Angus	Gaur	Holstein	0.050	18.911
Holstein	Banteng	Finncattle	0.093	25.387	Holstein	Gaur	Finncattle	0.094	25.585
Jersey	Banteng	Finncattle	0.094	25.707	Jersey	Gaur	Finncattle	0.095	26.336
Jersey	Banteng	Angus	0.076	17.657	Jersey	Gaur	Angus	0.077	17.778
Jersey	Banteng	Holstein	0.062	22.300	Jersey	Gaur	Holstein	0.063	22.408
Simmental	Banteng	Finncattle	0.104	26.536	Simmental	Gaur	Finncattle	0.104	26.161
Simmental	Banteng	Angus	0.078	16.969	Simmental	Gaur	Angus	0.078	16.667
Simmental	Banteng	Holstein	0.071	23.600	Simmental	Gaur	Holstein	0.070	22.671
Simmental	Banteng	Jersey	0.197	28.633	Simmental	Gaur	Jersey	0.196	28.268
**PopA**	**PopB**	**Pop C**	f_3	***Z* scores**	**PopA**	**PopB**	**Pop C**	f_3	***Z* scores**
**SAI**	**Banteng**	**SAI**			**SAI**	**Gaur**	**SAI**		
Cholistani	Banteng	Dhanni	0.025	20.949	Cholistani	Gaur	Dhanni	0.025	21.095
Cholistani	Banteng	Red Sindhi	0.024	15.202	Cholistani	Gaur	Red Sindhi	0.024	15.465
Dhanni	Banteng	Red Sindhi	0.026	18.591	Dhanni	Gaur	Red Sindhi	0.026	18.745
Indicine	Banteng	Cholistani	0.103	39.780	Indicine	Gaur	Cholistani	0.103	40.191
Indicine	Banteng	Dhanni	0.029	26.028	Indicine	Gaur	Dhanni	0.029	26.152
Indicine	Banteng	Red Sindhi	0.026	19.810	Indicine	Gaur	Red Sindhi	0.027	20.267
**PopA**	**PopB**	**Pop C**	f_3	***Z* scores**	**PopA**	**PopB**	**Pop C**	f_3	***Z* scores**
**Taurine**	**SAI**	**Taurine**			**Taurine**	**SAI**	**Taurine**		
Angus	Cholistani	Holstein	0.050	18.269	Jersey	Indicine	Finncattle	0.096	25.997
Angus	Dhanni	Holstein	0.050	18.525	Jersey	Red Sindhi	Finncattle	0.096	25.931
Angus	Indicine	Holstein	0.051	18.791	Jersey	Cholistani	Finncattle	0.096	25.724
Angus	Red Sindhi	Holstein	0.050	18.660	Jersey	Dhanni	Finncattle	0.095	25.776
Angus	Indicine	Finncattle	0.092	24.173	Simmental	Cholistani	Angus	0.078	17.725
Angus	Red Sindhi	Finncattle	0.091	24.198	Simmental	Cholistani	Holstein	0.070	23.604
Angus	Cholistani	Finncattle	0.091	23.689	Simmental	Cholistani	Jersey	0.197	28.307
Angus	Dhanni	Finncattle	0.091	23.720	Simmental	Dhanni	Angus	0.079	17.696
Holstein	Indicine	Finncattle	0.095	25.168	Simmental	Dhanni	Holstein	0.071	23.878
Holstein	Red Sindhi	Finncattle	0.095	25.168	Simmental	Dhanni	Jersey	0.198	28.732
Holstein	Cholistani	Finncattle	0.095	24.933	Simmental	Indicine	Angus	0.078	17.553
Holstein	Dhanni	Finncattle	0.094	25.006	Simmental	Indicine	Holstein	0.071	24.126
Jersey	Cholistani	Angus	0.077	17.636	Simmental	Indicine	Jersey	0.196	28.406
Jersey	Cholistani	Holstein	0.062	21.072	Simmental	Red Sindhi	Angus	0.079	17.157
Jersey	Dhanni	Angus	0.077	17.646	Simmental	Red Sindhi	Holstein	0.071	24.150
Jersey	Dhanni	Holstein	0.062	22.182	Simmental	Red Sindhi	Jersey	0.197	28.683
Jersey	Indicine	Angus	0.077	17.607	Simmental	Indicine	Finncattle	0.105	27.381
Jersey	Indicine	Holstein	0.063	21.759	Simmental	Red Sindhi	Finncattle	0.106	27.449
Jersey	Red Sindhi	Angus	0.078	17.408	Simmental	Cholistani	Finncattle	0.105	27.017
Jersey	Red Sindhi	Holstein	0.063	21.727	Simmental	Dhanni	Finncattle	0.105	27.015
**PopA**	**PopB**	**Pop C**	f_3	***Z* scores**	**PopA**	**PopB**	**Pop C**	f_3	***Z* scores**
**SAI**	**Taurine**	**SAI**			**SAI**	**Taurine**	**SAI**		
Cholistani	Angus	Dhanni	0.024	10.984	Indicine	Angus	Cholistani	0.109	31.472
Cholistani	Angus	Red Sindhi	0.022	8.735	Indicine	Angus	Dhanni	0.032	15.995
Cholistani	Finncattle	Dhanni	0.024	10.967	Indicine	Angus	Red Sindhi	0.030	11.699
Cholistani	Finncattle	Red Sindhi	0.022	8.910	Indicine	Finncattle	Cholistani	0.109	31.490
Cholistani	Holstein	Dhanni	0.024	11.325	Indicine	Finncattle	Dhanni	0.032	15.798
Cholistani	Holstein	Red Sindhi	0.022	8.946	Indicine	Finncattle	Red Sindhi	0.030	11.696
Cholistani	Jersey	Dhanni	0.024	11.165	Indicine	Holstein	Cholistani	0.108	31.373
Cholistani	Jersey	Red Sindhi	0.022	8.799	Indicine	Holstein	Dhanni	0.032	15.824
Cholistani	Simmental	Dhanni	0.024	11.112	Indicine	Holstein	Red Sindhi	0.029	11.665
Cholistani	Simmental	Red Sindhi	0.022	8.857	Indicine	Jersey	Cholistani	0.109	31.431
Dhanni	Angus	Red Sindhi	0.026	10.245	Indicine	Jersey	Dhanni	0.032	16.006
Dhanni	Finncattle	Red Sindhi	0.026	10.559	Indicine	Jersey	Red Sindhi	0.030	11.777
Dhanni	Holstein	Red Sindhi	0.026	10.419	Indicine	Simmental	Cholistani	0.109	31.603
Dhanni	Jersey	Red Sindhi	0.026	10.417	Indicine	Simmental	Dhanni	0.032	15.840
Dhanni	Simmental	Red Sindhi	0.026	10.491	Indicine	Simmental	Red Sindhi	0.029	11.696

Supplementary Table 8 Results from the enrichment analysis of genes introgressed from banteng and gaur into East Asian indicine (EAT) cattle based on the *U20* statistic. The GO and KEGG analyses were performed with KOBAS v3.0 based on the lists of genes present in genomic regions introgressed from both banteng and gaur into EAI cattle. The *P* value was calculated using a hypergeometric distribution. False discovery rate (FDR) correction was performed to adjust for multiple testing. Pathways with an FDR-corrected *P* value of < 0.05 were considered statistically significantly enriched.

**Terms**	**ID**	**Input genes**	**No. of genes**	**Corrected *P* values**	**Genes**
Axon guidance	bta04360	26	180	0.01003	*EFNB2|NFATC2|SRC|EPHA7|EPHA3|BMPR1B|SEMA4A|SEMA6D|MYL9|PLXNB1|PARD6G|NRAS|DCC|SLIT3|SLIT2|ARHGEF11|CAMK2D|UNC5D|PLXNC1|SEMA3E|SEMA3D|NCK2|ROBO1|PLXNA2|GSK3B|PPP3CA*
Metabolic pathways	bta01100	122	1468	0.01003	*GSS|AGK|FHIT|PTDSS1|GNPDA2|GPAT4|NDUFB2|ISPD|MOCS1|HPSE2|UQCRB|NDUFAB1|GPAM|DCK|ACSM3|PIK3CG|SPTLC2|PCCA|B3GALT1|ENPP7|CHSY3|IMPA1|PIK3C3|PDE8B|NME4|FDPS|NME6|UCKL1|ACSL5|CHAC2|UQCRC1|GGCX|GSTZ1|UOX|GAPDH|TPK1|COX6B2|COX7B2|IPMK|PANK2|CEL|GUCY1A1|PAICS|B4GALT1|PLCB1|PLCB4|UAP1|MAN2A1|RIMKLB|ACO1|COX7A2|GALNT1|CSGALNACT1|MTHFD1L|HMOX2|ATP6V1G1|PDE6H|POMT2|PIGU|MUT|AMPD1|NAMPT|MBOAT1|PGAM2|HGSNAT|FKTN|GAD1|PKLR|AKR1B10|GBGT1|NDUFA5|ACSS2|SEPHS1|GMPPB|GALC|SLC27A5|AKR1B1|GALNT13|AK7|PPAT|PDE4A|PDE4D|GNS|ST6GAL2|GGT7|FLAD1|SGMS1|ATP5F1C|ACER2|IDH3B|LPIN3|ASAH2|AASS|CHIT1|DGKI|NT5C1B|IDNK|DGKB|EPRS|GAA|DNMT3B|PANK1|TDO2|MAT2A|ST3GAL5|CA13|PIGQ|SEPSECS|PLB1|PLA2G4A|ASAH1|MINPP1|ATP4A|CA3|CA2|PMVK|ITPA|NDUFS4|CANT1|AHCY|PDE1A|COQ3*
Oxytocin signaling pathway	bta04921	23	152	0.01100	*NFATC1|NFATC2|SRC|CDKN1A|EGFR|MAP2K5|MYL9|CALM1|RYR1|CACNB2|NRAS|PIK3CG|GUCY1A1|PPP1R12C|PLCB1|PLCB4|CAMK2D|PLA2G4A|KCNJ3|PPP1CB|KCNJ9|PRKACB|PPP3CA*
SNARE interactions in vesicular transport	bta04130	10	34	0.01217	*VAMP1|VAMP5|VAMP8|STX3|STX2|STX7|STX6|VTI1A|STX17|STX18*
Parathyroid hormone synthesis, secretion and action	bta04928	18	104	0.01231	*PLCB1|MMP16|VDR|PLCB4|NR4A2|LRP6|CREB5|PRKACB|EGFR|MMP24|MEF2D|BGLAP|SLC34A2|BRAF|CASR|PDE4A|PDE4D|CDKN1A*
Long-term depression	bta04730	13	60	0.01499	*PLCB1|IGF1|PLCB4|GRID2|RYR1|NRAS|GUCY1A1|GRIA2|PRKG1|BRAF|PRKG2|PLA2G4A|GRM1*
Salivary secretion	bta04970	16	93	0.02478	*PLCB1|CAMP|PLCB4|ADRA1D|CALM1|CATHL4|PRKG2|CATHL1|LPO|ADRB1|ATP1A4|PRKG1|ATP1A2|PRKACB|GUCY1A1|ATP2B4*
Calcium signaling pathway	bta04020	26	203	0.02787	*PTGFR|EGFR|STIM2|PTK2B|EDNRB|CYSLTR2|TRDN|CASQ1|CALM1|RYR1|PDE1A|CACNA1G|GRM5|GRM1|PLCB1|PLCB4|CAMK2D|CHRM2|TACR3|ATP2B4|ADRA1D|DRD5|CCKAR|ADRB1|PRKACB|PPP3CA*
Circadian entrainment	bta04713	16	100	0.03950	*KCNJ3|PLCB1|PLCB4|RPS6KA5|CALM1|RYR1|KCNJ9|PRKG2|CACNA1G|GUCY1A1|GRIA2|GNG5|PRKG1|PRKACB|CAMK2D|NOS1AP*
Neuroactive ligand–receptor interaction	bta04080	38	363	0.04259	*PTGFR|ADORA1|GABRR1|GABRR2|PRP2|UCN2|EDNRB|CYSLTR2|OPRL1|PRP9|CGA|RXFP4|GRIA2|GRM8|TSHB|GRM5|GABRA2|GRM1|GRID2|PRP14|CHRM2|OPRM1|CHRNA2|GABRB1|PTH2|TACR3|ADRA1D|PRP-VII|C3AR1|GLRA1|DRD5|CCKAR|ADRB1|GALR1|NPBWR2|GRIK1|HTR1F|GRIK2*
Purine metabolism	bta00230	19	135	0.04579	*NME4|PKLR|NME6|DCK|PDE1A|UOX|AK7|GUCY1A1|PAICS|ITPA|PPAT|NT5C1B|CANT1|PDE4A|PDE6H|PDE4D|AMPD1|PDE8B|FHIT*
cGMP-PKG signaling pathway	bta04022	22	169	0.04579	*NFATC1|NFATC2|ADORA1|EDNRB|MYL9|CALM1|PIK3CG|GUCY1A1|PRKG1|PRKG2|PLCB1|PLCB4|CREB5|ATP2B4|KCNMB2|PPP1CB|ADRA1D|ADRB1|ATP1A4|ATP1A2|MEF2D|PPP3CA*
Oxygen binding	GO:0019825	6	13	0.03047	*HBM|NGB|HBQ1|HBA|ALB|HBZ*
Glutamate receptor activity	GO:0008066	6	13	0.03047	*GRID2|GRIK1|GRIK2|GRM8|GRM5|GRM1*
Parallel fiber to Purkinje cell synapse	GO:0098688	6	13	0.03047	*KCNJ3|PLCB4|GRID2|CADPS2|KCNJ9|CTNNA2*
Glial cell differentiation	GO:0010001	6	14	0.03900	*PHOX2B|CRB1|MMP24|CDH2|FGF2|DNER*
Oxygen transport	GO:0015671	6	14	0.03900	*HBM|NGB|HBQ1|HBA|HBZ|HBA1*
DNA damage response, signal transduction by p53 class mediator resulting in cell cycle arrest	GO:0006977	5	9	0.04259	*PML|CDKN1A|MDM2|CRADD|SOX4*

Supplementary Table 9 Top candidate genes associated with adaptive introgression from banteng into East Asian indicine cattle. Only the genomic regions containing SNPs that share banteng alleles at a frequency of greater than 50% in East Asian indicine cattle and less than 1% in South Asian indicine cattle are shown. Adjacent intervals have been merged.

BTA	Start	End	Length	Total SNPs	*U*50	Genes
1	66690001	66800000	109999	882	508	*CSTA|CCDC58|ILDR1|CASR*
6	69300001	69440000	139999	562	448	
6	69500001	69600000	99999	334	307	*CHIC2*
6	69800001	69900000	99999	370	297	
6	70100001	70250000	149999	1112	897	*KIT*
8	11490001	11690000	199999	1180	655	*TOPORS|NDUFB6|DDX58|ACO1*
8	96290001	96400000	109999	694	303	
13	62750001	62800000	49999	71	51	*BPIFB5|BPIFB1*
13	62920001	63200000	279999	2544	1622	*C13H20orf144|E2F1|NECAB3|PXMP4|CBFA2T2|ZNF341*
13	63320001	63370000	49999	84	55	
13	63420001	63970000	549999	32996	2776	*EIF2S2|RALY |MAP1LC3A|ASIP|DYNLRB1|AHCY|ITCH|PIGU*
13	64090001	64170000	79999	237	164	*GGT7|NCOA6*
18	60240001	60340000	99999	389	326	*LOC101905616|LOC786224|LOC616720*
18	60450001	60500000	49999	112	51	*LOC618456|LOC104968479*
18	60650001	60740000	89999	599	198	*LOC112442373|LOC100139104|LOC100336448|ZNF845*
19	52090001	52150000	59999	229	144	*ENDOV|LOC509283*
20	540001	600000	59999	208	106	*SLIT3*
24	53920001	54000000	79999	416	210	*LOC100137989|LOC101904580*
24	54070001	54170000	99999	635	305	
25	70001	170000	99999	662	322	*POLR3K|SNRNP25|IL9R|MPG|NPRL3|RHBDF1*
25	190001	300000	109999	1243	574	*HBZ|HBM|HBA|HBA1|HBQ1|RGS11|FAM234A|LUC7L*
26	11020001	11090000	69999	211	107	*IFIT2|IFIT3|IFIT5|LOC100139670*

Supplementary Table 10 Top candidate genes associated with adaptive introgression from gaur into East Asian indicine cattle. Only the regions containing SNPs that share gaur alleles at a frequency of more than 50% in East Asian indicine cattle but less than 1% in South Asian indicine cattle are shown. Adjacent intervals have been merged.

BTA	Start	End	Length	Total SNPs	*U*50	Genes
1	66690001	66790000	99999	648	442	*CSTA|ILDR1|CASR*
6	69390001	69440000	49999	126	71	
6	69500001	69600000	99999	415	297	*CHIC2*
6	69820001	69890000	69999	267	127	
6	70120001	70220000	99999	477	352	*KIT*
8	11570001	11650000	79999	274	216	*DDX58|ACO1*
8	96290001	96340000	49999	69	53	
8	96340001	96400000	59999	148	126	
13	62740001	62850000	109999	525	344	*BPIFB5|CDK5RAP1|BPIFB1*
13	62940001	63200000	259999	2023	1262	*C13H20orf144|E2F1|NECAB3|PXMP4|CBFA2T2|ZNF341*
13	63400001	63520000	119999	437	343	*EIF2S2|RALY*
13	63720001	63850000	129999	575	376	*ITCH*
13	63890001	63990000	99999	436	279	*MAP1LC3A|DYNLRB1|PIGU*
13	64040001	64170000	129999	770	411	*TP53INP2|GGT7|NCOA6*
13	64200001	64250000	49999	79	56	*GSS|ACSS2*
18	60240001	60340000	99999	452	397	*LOC101905616|LOC786224|LOC616720*
18	60450001	60500000	49999	123	57	*LOC618456|LOC104968479*
18	60650001	60740000	89999	492	205	*LOC112442373|LOC100139104|LOC100336448|ZNF845*
19	52090001	52150000	59999	251	154	*ENDOV|LOC509283*
20	350001	440000	89999	445	201	
20	440001	570000	129999	424	215	*SLIT3*
21	21570001	21640000	69999	207	118	*CIB1|NGRN|SEMA4B|GDPGP1*
24	1	90000	89999	275	207	
24	53940001	54020000	79999	347	201	*LOC100137989|DYNAP*
24	54090001	54170000	79999	290	196	
25	70001	170000	99999	619	321	*POLR3K|SNRNP25|IL9R|MPG|NPRL3|RHBDF1*
25	190001	300000	109999	1058	565	*HBZ|HBM|HBA|HBA1|HBQ1|RGS11|FAM234A|LUC7L*

Supplementary References

1. Bolger, A.M., Lohse, M. & Usadel, B. Trimmomatic: a flexible trimmer for Illumina sequence data. *Bioinformatics* **30**, 2114-2120 (2014).
2. Abuín, J.M., Pichel, J.C., Pena, T.F. & Amigo, J. BigBWA: approaching the Burrows–Wheeler aligner to Big Data technologies. *Bioinformatics* **31**, 4003-4005 (2015).
3. Li, H., et al. The Sequence Alignment/Map format and SAMtools. *Bioinformatics* **25**, 2078-2079 (2009).
4. McKenna, A., et al. The Genome Analysis Toolkit: A MapReduce framework for analyzing next-generation DNA sequencing data. *Genome Res.* **20**, 1297-1303 (2010).
5. Browning, S.R. & Browning, B.L. Rapid and accurate haplotype phasing and missing-data inference for whole-genome association studies by use of localized haplotype clustering. *Am. J. Hum. Genet* **81**, 1084-1097 (2007).
6. Cingolani, P., et al. A program for annotating and predicting the effects of single nucleotide polymorphisms, SnpEff: SNPs in the genome of Drosophila melanogaster strain w^{1118}; iso-2; iso-3. *Fly* **6**, 80-92 (2012).
7. Danecek, P., et al. The variant call format and VCFtools. *Bioinformatics* **27**, 2156-2158 (2011).
8. Weir, B.S. & Cockerham, C.C. Estimating F-statistics for the analysis of population structure. *Evolution* **38**, 1358-1370 (1984).
9. Purcell, S., et al. PLINK: a tool set for whole-genome association and population-based linkage analyses. *Am. J. Hum. Genet* **81**, 559-575 (2007).
10. Patterson, N., Price, A.L. & Reich, D. Population structure and eigenanalysis. *PLoS Genet.* **2**, e190-e190 (2006).
11. Gómez-Rubio, V. ggplot2-elegant graphics for data analysis (2nd Edition). *Journal of Statistical Software, Book Reviews* **77**, 1-3 (2017).
12. Alexander, D.H., Novembre, J. & Lange, K. Fast model-based estimation of ancestry in unrelated individuals. *Genome Res.* **19**, 1655-1664 (2009).
13. Pickrell, J.K. & Pritchard, J.K. Inference of population splits and mixtures from genome-wide allele frequency data. *PLoS Genet.* **8**, e1002967-e1002967 (2012).
14. Szpiech, Z.A. & Hernandez, R.D. selscan: an efficient multithreaded program to perform EHH-based scans for positive selection. *Mol. Biol. Evol.* **31**, 2824-2827 (2014).
15. DeGiorgio, M., Huber, C.D., Hubisz, M.J., Hellmann, I. & Nielsen, R. SweepFinder2: increased sensitivity, robustness and flexibility. *Bioinformatics* **32**, 1895-1897 (2016).
16. Yi, X., et al. Sequencing of 50 human exomes reveals adaptation to high altitude. *Science* **329**, 75-78 (2010).
17. Bu, D., et al. KOBAS-i: intelligent prioritization and exploratory visualization of biological functions for gene enrichment analysis. *Nucleic Acids Res.* **49**, W317-W325 (2021).
18. Patterson, N., et al. Ancient admixture in human history. *Genetics* **192**, 1065-1093 (2012).
19. Malinsky, M., Matschiner, M. & Svardal, H. Dsuite-Fast D-statistics and related admixture evidence from VCF files. *Mol. Ecol. Resour.* **21**, 584-595 (2021).
20. Maples, B.K., Gravel, S., Kenny, E.E. & Bustamante, C.D. RFMix: a discriminative modeling approach for rapid and robust local-ancestry inference. *Am. J. Hum. Genet.* **93**, 278-288 (2013).
21. Huerta-Sánchez, E., et al. Altitude adaptation in Tibetans caused by introgression of Denisovan-like DNA. *Nature* **512**, 194-197 (2014).
22. Wu, D.-D., et al. Pervasive introgression facilitated domestication and adaptation in the Bos species complex. *Nat. Ecol. Evol.* **2**, 1139-1145 (2018).
23. Nguyen, L.-T., Schmidt, H.A., von Haeseler, A. & Minh, B.Q. IQ-TREE: a fast and effective stochastic algorithm for estimating maximum-likelihood phylogenies. *Mol. Biol. Evol.* **32**, 268-274 (2014).
24. Bouckaert, R.R. DensiTree: making sense of sets of phylogenetic trees. *Bioinformatics* **26**, 1372-1373 (2010).
25. Racimo, F., Marnetto, D. & Huerta-Sánchez, E. Signatures of archaic adaptive introgression in present-day human populations. *Mol. Biol. Evol.* **34**, 296-317 (2016).
26. Chang, T.-C., Yang, Y., Retzel, E.F. & Liu, W.-S. Male-specific region of the bovine Y chromosome is gene rich with a high transcriptomic activity in testis development. *Proc. Natl. Acad. Sci. USA* **110**, 12373-12378 (2013).
27. Kumar, S., Stecher, G. & Tamura, K. MEGA7: Molecular Evolutionary Genetics Analysis Version 7.0 for Bigger Datasets. *Mol. Biol. Evol.* **33**, 1870-1874 (2016).
28. Bouckaert, R., et al. BEAST 2.5: An advanced software platform for Bayesian evolutionary analysis. *PLoS Comp. Biol.* **15**, e1006650 (2019).
29. Bandelt, H.J., Forster, P. & Röhl, A. Median-joining networks for inferring intraspecific phylogenies.

Mol. Biol. Evol. **16**, 37-48 (1999).
30. Liu, G.E., Matukumalli, L.K., Sonstegard, T.S., Shade, L.L. & Van Tassell, C.P. Genomic divergences among cattle, dog and human estimated from large-scale alignments of genomic sequences. *BMC Genom.* **7**, 140 (2006).
31. Rambaut, A., Drummond, A.J., Xie, D., Baele, G. & Suchard, M.A. Posterior summarization in Bayesian phylogenetics using Tracer 1.7. *Syst. Biol.* **67**, 901-904 (2018).
32. Tsai, I.J., Otto, T.D. & Berriman, M. Improving draft assemblies by iterative mapping and assembly of short reads to eliminate gaps. *Genome Biol.* **11**, R41 (2010).
33. Keane, T.M., Creevey, C.J., Pentony, M.M., Naughton, T.J. & McLnerney, J.O. Assessment of methods for amino acid matrix selection and their use on empirical data shows that ad hoc assumptions for choice of matrix are not justified. *BMC Evol. Biol.* **6**, 29 (2006).
34. Stamatakis, A. RAxML-VI-HPC: maximum likelihood-based phylogenetic analyses with thousands of taxa and mixed models. *Bioinformatics* **22**, 2688-2690 (2006).
35. Achilli, A., et al. Mitochondrial genomes of extinct aurochs survive in domestic cattle. *Curr. Biol.* **18**, R157-R158 (2008).
36. Drummond, A.J., Suchard, M.A., Xie, D. & Rambaut, A. Bayesian phylogenetics with BEAUti and the BEAST 1.7. *Mol. Biol. Evol.* **29**, 1969-1973 (2012).
37. Bollongino, R., et al. Modern taurine cattle descended from small number of Near-Eastern founders. *Mol. Biol. Evol.* **29**, 2101-2104 (2012).
38. Schiffels, S. & Wang, K. MSMC and MSMC2: The Multiple Sequentially Markovian Coalescent. In: *Statistical Population Genomics* (ed Dutheil JY). Springer US (2020).
39. Chen, N., et al. Whole-genome resequencing reveals world-wide ancestry and adaptive introgression events of domesticated cattle in East Asia. *Nat. Commun.* **9**, 2337 (2018).
40. Dachs, N., et al. Quantitative trait locus for calving traits on Bos taurus autosome 18 in Holstein cattle is embedded in a complex genomic region. *J. Dairy Sci.* **106**, 1925-1941 (2023).
41. Kazantseva, A., et al. Human hair growth deficiency is linked to a genetic defect in the phospholipase gene *LIPH*. *Science* **314**, 982 (2006).
42. Ai, H., et al. Adaptation and possible ancient interspecies introgression in pigs identified by whole-genome sequencing. *Nat. Genet.* **47**, 217-225 (2015).
43. Peirson, S.N., et al. Microarray analysis and functional genomics identify novel components of melanopsin signaling. *Curr. Biol.* **17**, 1363-1372 (2007).
44. Hankins, M.W., Peirson, S.N. & Foster, R.G. Melanopsin: an exciting photopigment. *Trends Neurosci.* **31**, 27-36 (2008).
45. Ali, N.S., Sartori-Valinotti, J.C. & Bruce, A.J. Periodic fever, aphthous stomatitis, pharyngitis, and adenitis (PFAPA) syndrome. *Clin. Dermatol.* **34**, 482-486 (2016).
46. Duchesne, A., et al. Progressive ataxia of Charolais cattle highlights a role of KIF1C in sustainable myelination. *PLoS Genet.* **14**, e1007550 (2018).
47. Fougerousse, F., et al. The muscle-specific enolase is an early marker of human myogenesis. *J. Muscle Res. Cell Motil.* **22**, 535-544 (2001).
48. Lee, C.-J., Yoon, M.-J., Kim, D.H., Kim, T.U. & Kang, Y.-J. Profilin-1; a novel regulator of DNA damage response and repair machinery in keratinocytes. *Mol. Biol. Rep.* **48**, 1439-1452 (2021).
49. Miyajima, D., et al. Profilin1 regulates sternum development and endochondral bone formation. *J. Biol. Chem.* **287**, 33545-33553 (2012).
50. Mahmoudi, S., et al. Wrap53, a natural p53 antisense transcript required for p53 induction upon DNA damage. *Mol. Cell* **33**, 462-471 (2009).
51. Palpant, N.J., et al. Transmembrane protein 88: a Wnt regulatory protein that specifies cardiomyocyte development. *Development (Cambridge, England)* **140**, 3799-3808 (2013).
52. Yang, R.M., et al. TAMM41 is required for heart valve differentiation via regulation of PINK-PARK2 dependent mitophagy. *Cell Death Differ.* **26**, 2430-2446 (2019).
53. Jongstra-Bilen, J. & Jongstra, J. Leukocyte-specific protein 1 (LSP1). *Immunologic Research* **35**, 65-73 (2006).
54. Toydemir, R.M. & Bamshad, M.J. Sheldon-Hall syndrome. *Orphanet J Rare Dis.* **4**, 11-11 (2009).
