# Supplementary material for: Global genetic diversity, introgression, and evolutionary adaptation of indicine cattle revealed by whole genome sequencing
Source: Nat Commun. 2023 Nov 28;14:7803. doi: 10.1038/s41467-023-43626-z (PMC10684552; doi:10.1038/s41467-023-43626-z)
Supplement: Supplementary file 3 — Description of Additional Supplementary Files [file 41467_2023_43626_MOESM3_ESM.pdf]

Description of Additional Supplementary Files

Supplementary Data 1: Overview of the sampling information and sequencing statistics.

Supplementary Data 2: Common candidate genomic regions identified in South Asian indicine cattle based on the CLR, *iHS*, F_{ST}, and $\vartheta\pi$ analyses.

Supplementary Data 3: Common candidate genomic regions shared by South Asian indicine (SAI) and African indicine (AFI) cattle based on the *iHS* (proportion of SNPs with |*iHS*| >=2) and CLR analyses.

Supplementary Data 4: *D* statistic among indicine, taurine cattle, banteng, and gaur to select pure indicine, taurine, banteng, and gaur samples.

Supplementary Data 5: Results of the *D* statistics (swamp buffalo as outgroup) tests performed to detect admixtures among 4 banteng, 2 gaur, 15 South Asian indicine, and 15 taurine cattle.

Supplementary Data 6: Statistics on introgressed intervals from banteng into 97 East Asian and Southwest Chinese indicine cattle.

Supplementary Data 7: Statistics on introgressed intervals from gaur into 97 East Asian and Southwest Chinese indicine cattle.

Supplementary Data 8: Sampling information of taurindicine cattle breeds from North-Central China.
